# Supplementary material for: Folic acid supplementation during preconception period in sub-Saharan African countries: A systematic review and meta-analysis
Source: PLoS One. 2025 Jan 31;20(1):e0318422. doi: 10.1371/journal.pone.0318422 (PMC11785287; doi:10.1371/journal.pone.0318422)
Supplement: S2 Table — (DOCX) [file pone.0318422.s002.docx]

**Supplementary 2. Results from different sources for folic acid supplementation during the preconception period in sub-Saharan African countries: A systematic-review and meta-analysis**

| **Type of database** | **Number of articles identified** | **Search terms** | **Accessed Date and time** |
| --- | --- | --- | --- |
| **Pub med** | **774** | **((((((((((((('Folic acid'[MeSH Terms]) OR (Folic acid[Title/Abstract])) OR (folate[Title/Abstract])) OR (vitamin[Title/Abstract])) OR ('Pteroylglutamic acid'[Title/Abstract])) OR ('Folic acid supplementation'[Title/Abstract])) OR ('Folic acid administration'[Title/Abstract])) OR ('Folic acid adherence'[Title/Abstract])) OR ('Folinic acid'[Title/Abstract])) OR ('Vitamin B9'[Title/Abstract]))) AND ((((((((((('Preconception care'[Title/Abstract]) OR (preconception[Title/Abstract])) OR (pre-conception[Title/Abstract])) OR (periconception[Title/Abstract])) OR (peri-conception[Title/Abstract])) OR ('before conception'[Title/Abstract])) OR (prior-conception[Title/Abstract])) OR (pre-pregnancy[Title/Abstract])) OR ('Pregnant women'[Title/Abstract])) OR (Pregnancy[Title/Abstract])))) AND ((("Angola"[Title/Abstract] OR "Benin"[Title/Abstract] OR "Botswana"[Title/Abstract] OR "Burkina Faso"[Title/Abstract] OR "Burundi"[Title/Abstract] OR "Cameroon"[Title/Abstract] OR "Cape Verde"[Title/Abstract] OR "Central African Republic"[Title/Abstract] OR "Chad"[Title/Abstract] OR "Comoros"[Title/Abstract] OR "Congo (Brazzaville)"[Title/Abstract] OR "Congo (Democratic Republic)"[Title/Abstract] OR "Côte d'Ivoire"[Title/Abstract] OR "Djibouti"[Title/Abstract] OR "Equatorial Guinea"[Title/Abstract] OR "Eritrea"[Title/Abstract] OR "Ethiopia"[Title/Abstract] OR "Gabon"[Title/Abstract] OR "The Gambia"[Title/Abstract] OR "Ghana"[Title/Abstract] OR "Guinea"[Title/Abstract] OR "Guinea-Bissau"[Title/Abstract] OR "Kenya"[Title/Abstract] OR "Lesotho"[Title/Abstract] OR "Liberia"[Title/Abstract] OR "Madagascar"[Title/Abstract] OR "Malawi"[Title/Abstract] OR "Mali"[Title/Abstract] OR "Mauritania"[Title/Abstract] OR "Mauritius"[Title/Abstract] OR "Mozambique"[Title/Abstract] OR "Namibia"[Title/Abstract] OR "Niger"[Title/Abstract] OR "Nigeria"[Title/Abstract] OR "Réunion"[Title/Abstract] OR "Rwanda"[Title/Abstract] OR "Sao Tome and Principe"[Title/Abstract] OR "Senegal"[Title/Abstract] OR "Seychelles"[Title/Abstract] OR "Sierra Leone"[Title/Abstract] OR "Somalia"[Title/Abstract] OR "South Africa"[Title/Abstract] OR "Sudan"[Title/Abstract] OR "Swaziland"[Title/Abstract] OR "Tanzania"[Title/Abstract] OR "Togo"[Title/Abstract] OR "Uganda"[Title/Abstract] OR "Western Sahara"[Title/Abstract] OR "Zambia"[Title/Abstract] OR "Zimbabwe"[Title/Abstract]))))** | **12:15 AM on Monday, January 29, 2024** |
| **Scopus** | **61** | ( TITLE-ABS-KEY ( "Folic acid" OR folate OR vitamin OR "Pteroylglutamic acid" OR "Folic acid supplementation" OR "Folic acid administration" OR "Folic acid adherence" OR "Folinic acid" OR "Vitamin B9" ) AND TITLE-ABS-KEY ( "Preconception Care" OR preconception OR pre-conception OR preconception OR peri-conception OR " before conception" OR prior-conception OR pre-pregnancy OR pregnancy ) AND TITLE-ABS-KEY ( angola OR benin OR botswana OR burkina AND faso OR burundi OR cameroon OR "Cape Verde" OR "Central African Republic" OR chad OR comoros OR "Congo (Brazzaville)" OR "Congo (Democratic Republic)" OR "Côte d’Ivoire" OR djibouti OR "Equatorial Guinea" OR eritrea OR ethiopia OR gabon OR "The Gambia" OR ghana OR guinea OR "Guinea-Bissau" OR kenya OR lesotho OR liberia OR madagascar OR malawi OR mali OR mauritania OR mauritius OR mozambique OR namibia OR niger OR nigeria OR réunion OR rwanda OR "Sao Tome and Principe" OR senegal OR seychelles OR "Sierra Leone" OR somalia OR "South Africa" OR sudan OR swaziland OR tanzania OR togo OR uganda OR "Western Sahara" OR zambia OR zimbabwe ) ) | **12:50 AM on Monday, January 29, 2024** |
| **Embase** | **212** | ('preconception care':ab,ti,kw OR 'preconception':ab,ti,kw OR 'pre-conception':ab,ti,kw OR 'periconception':ab,ti,kw OR 'peri-conception':ab,ti,kw OR 'before conception':ab,ti,kw OR 'prior-conception':ab,ti,kw OR 'pre-pregnancy':ab,ti,kw OR 'pregnancy':ab,ti,kw) AND ((angola:ab,ti,kw OR benin:ab,ti,kw OR botswana:ab,ti,kw OR 'burkina faso':ab,ti,kw OR burundi:ab,ti,kw OR 'cabo verde':ab,ti,kw OR cameroon:ab,ti,kw OR 'central african republic':ab,ti,kw OR chad:ab,ti,kw OR comoros:ab,ti,kw OR 'congo, dem. rep.':ab,ti,kw OR 'congo, rep.':ab,ti,kw OR 'cote divoire':ab,ti,kw OR 'equatorial guinea':ab,ti,kw OR eritrea:ab,ti,kw OR eswatini:ab,ti,kw OR ethiopia:ab,ti,kw OR gabon:ab,ti,kw OR gambia,:ab,ti,kw OR ghana:ab,ti,kw OR guinea:ab,ti,kw OR 'guinea bissau':ab,ti,kw OR kenya:ab,ti,kw OR lesotho:ab,ti,kw OR liberia:ab,ti,kw OR madagascar:ab,ti,kw OR malawi:ab,ti,kw OR mali:ab,ti,kw OR mauritania:ab,ti,kw OR mauritius:ab,ti,kw OR mozambique:ab,ti,kw OR namibia:ab,ti,kw OR niger:ab,ti,kw OR nigeria:ab,ti,kw OR rwanda:ab,ti,kw OR 'sao tome':ab,ti,kw) AND principe:ab,ti,kw OR senegal:ab,ti,kw OR seychelles:ab,ti,kw OR 'sierra leone':ab,ti,kw OR somalia:ab,ti,kw OR 'south africa':ab,ti,kw OR 'south sudan':ab,ti,kw OR sudan:ab,ti,kw OR tanzania:ab,ti,kw OR togo:ab,ti,kw OR uganda:ab,ti,kw OR zambia:ab,ti,kw OR zimbabwe:ab,ti,kw) | **11:15 AM on Monday, January 29, 2024** |
| **Hinari** | **55** | (TitleCombined:("Folic acid" OR folate OR vitamin OR "Pteroylglutamic acid" OR "Folic acid supplementation" OR "Folic acid administration" OR "Folic acid adherence" OR "Folinic acid" OR "Vitamin B9")) AND (TitleCombined:("Preconception Care" OR preconception OR pre-conception OR preconception OR peri-conception OR " before conception" OR prior-conception OR pre-pregnancy OR pregnancy)) AND (TitleCombined: ( Angola OR Benin OR Botswana OR "Burkina Faso" OR Burundi OR Cameroon OR "Cape Verde" OR "Central African Republic" OR Chad OR Comoros OR "Congo (Brazzaville)" OR "Congo (Democratic Republic)" OR "Côte d’Ivoire" OR Djibouti OR "Equatorial Guinea" OR Eritrea OR Ethiopia OR Gabon OR "The Gambia" OR Ghana OR Guinea OR "Guinea-Bissau" OR Kenya OR Lesotho OR Liberia OR Madagascar OR Malawi OR Mali OR Mauritania OR Mauritius OR Mozambique OR Namibia OR Niger OR Nigeria OR Réunion OR Rwanda OR "Sao Tome And Principe" OR Senegal OR Seychelles OR "Sierra Leone" OR Somalia OR "South Africa" OR Sudan OR Swaziland OR Tanzania OR Togo OR Uganda OR "Western Sahara" OR Zambia OR Zimbabwe) |  |
| **From google and google scholar** | **155** |  |  |
| **Grey** | **2** |  |  |
| **Total** | **1259** |  |  |
