# Supplementary material for: Folic acid supplementation during preconception period in sub-Saharan African countries: A systematic review and meta-analysis
Source: PLoS One. 2025 Jan 31;20(1):e0318422. doi: 10.1371/journal.pone.0318422 (PMC11785287; doi:10.1371/journal.pone.0318422)
Supplement: S3 Table — (DOCX) [file pone.0318422.s003.docx]

Supplementary 3. Methodological quality assessment of included studies using Newcastle-Ottawa Scale (NOS) for preconceptional FA supplementation in SSA, 2024

| **No.** | **Author/year** | **Representativeness** | **Sample size** | **Non-respondents** | **Ascertainment of the exposure** | **comparability** | **Assessment of outcome** | **Statistical test** | **Quality score** |
| --- | --- | --- | --- | --- | --- | --- | --- | --- | --- |
| 1 | Akinajo et al.2019 | 1 | 0 | 1 | 2 | 1 | 1 | 1 | **7** |
| 2 | Alemajo et al. 2022 | 1 | 1 | 1 | 2 | 1 | 1 | 1 | **8** |
| 3 | Alsammani et al. 2021 | 1 | 1 | 1 | 1 | 1 | 1 | 1 | **7** |
| 4 | Amaje et al. / 2022 | 1 | 1 | 1 | 1 | 1 | 1 | 1 | **7** |
| 5 | Asresu et al. / 2019 | 1 | 1 | 1 | 2 | 1 | 1 | 1 | **8** |
| 6 | Anzaku A. / 2014 | 1 | 1 | 1 | 1 | 1 | 1 | 1 | **7** |
| 7 | Ayele A. et al. / 2022 | 1 | 1 | 1 | 2 | 1 | 1 | 1 | **8** |
| 8 | Dessie et al. / 2017 | 1 | 1 | 1 | 1 | 1 | 1 | 1 | **7** |
| 9 | Ekem et al. / 2018 | 1 | 1 | 1 | 1 | 1 | 1 | 1 | **7** |
| 10 | Fekene et al. / 2020 | 1 | 1 | 1 | 1 | 1 | 1 | 1 | **7** |
| 11 | Fetena N.et al. / 2023 | 1 | 1 | 1 | 1 | 1 | 1 | 1 | **7** |
| 12 | Gamshe E and DDB /2021 | 1 | 1 | 1 | 2 | 1 | 1 | 1 | **8** |
| 13 | Gedefaw et al. / 2018 | 1 | 1 | 1 | 1 | 1 | 1 | 1 | **7** |
| 14 | Gelgalu et al. / 2021 | 1 | 1 | 1 | 1 | 1 | 1 | 1 | **7** |
| 15 | Habte et al. / 2040 | 1 | 1 | 1 | 1 | 1 | 1 | 1 | **7** |
| 16 | Hassan et al. 2024 | 1 | 1 | 1 | 1 | 1 | 1 | 1 | **7** |
| 17 | Joyce C. et al. 2018 | 1 | 1 | 1 | 1 | 1 | 1 | 1 | **7** |
| 18 | Mohammed B.et al. 2019 | 1 | 1 | 1 | 1 | 1 | 1 | 1 | **7** |
| 19 | Mukhalisi A et al. 2022 | 1 | 1 | 1 | 2 | 1 | 1 | 1 | **8** |
| 20 | Ubong Akpan Okon et al.2020 | 1 | 1 | 1 | 1 | 1 | 1 | 1 | **7** |
| 21 | Olowokere, A.E et. al 2015 | 1 | 1 | 1 | 2 | 0 | 1 | 1 | **7** |
| 22 | Setegn Alie M et al. 2022 | 1 | 1 | 1 | 1 | 1 | 1 | 1 | **7** |
| 23 | Asumadu et al. / 2020 | 1 | 1 | 1 | 0 | 0 | 1 | 1 | **5** |
| 24 | Beyuo T et al. / 2021 | 1 | 1 | 1 | 0 | 0 | 1 | 1 | **5** |
| 25 | Nwaolisa H. et al 2021 | 1 | 1 | 0 | 0 | 0 | 1 | 1 | **4** |
| 26 | Boakye Y. et al. / 2028 | 1 | 0 | 1 | 1 | 0 | 1 | 1 | **5** |
| 27 | Adebo et al. / 2035 | 1 | 0 | 1 | 1 | 1 | 1 | 1 | **6** |
| 28 | Girma et al. / 2023 | 1 | 1 | 1 | 0 | 1 | 1 | 1 | **6** |
| 29 | Lawal1 T. /2014 | 1 | 1 | 1 | 0 | 1 | 1 | 1 | **6** |
