# Supplementary material for: Folic acid supplementation during preconception period in sub-Saharan African countries: A systematic review and meta-analysis
Source: PLoS One. 2025 Jan 31;20(1):e0318422. doi: 10.1371/journal.pone.0318422 (PMC11785287; doi:10.1371/journal.pone.0318422)
Supplement: S1 Appendix — (DOCX) [file pone.0318422.s004.docx]

S4 Table: Reasons for excluded primary studies

| N^o^ | Author, year | | | Title | | URL | | Main reason for  exclusion |  |
| --- | --- | --- | --- | --- | --- | --- | --- | --- | --- |
|  | | Zotor, F.B., Ellahi, B., Amuna, P. (2015) | Applying the food multimix concept for sustainable and nutritious diets | | <https://www.scopus.com/inward/record.uri?eid=2-s2.0-84948387849&doi=10.1017%2fS0029665115002372&partnerID=40&md5=a4d847093cda5a9c07f1da3800d59375> | | excluded by title and abstract | | |
|  | | Ziari et al. (1996) | Serum vitamin A, vitamin E, and beta-carotene levels in preeclamptic women in northern nigeria. | | <https://pubmed.ncbi.nlm.nih.gov/8863947/> | | excluded by title and abstract | | |
|  | | Zerfu TA, Umeta M, Baye K (2016) | Dietary diversity during pregnancy is associated with reduced risk of maternal anemia, preterm delivery, and low birth weight in a prospective cohort study in rural Ethiopia | | <https://pmc.ncbi.nlm.nih.gov/articles/PMC10576272/> | | excluded by title and abstract | | |
|  | | Zelka MA, Yalew AW, Debelew GT (2023) | The effects of adherence to recommended antenatal services on adverse pregnancy outcomes in Northwest Ethiopia: multilevel and propensity score matching (PSM) modeling. | | <https://pubmed.ncbi.nlm.nih.gov/37434914/> | | excluded by title and abstract | | |
|  | | ka MA, Yalew AW, Debelew GT (2022) | The effects of completion of continuum of care in maternal health services on adverse birth outcomes in Northwestern Ethiopia: a prospective follow-up study. | | <https://pubmed.ncbi.nlm.nih.gov/36209163> | | excluded by title and abstract | | |
|  | | Zagré et al (2007) | Prenatal multiple micronutrient supplementation has greater impact on birthweight than supplementation with iron and folic acid: a cluster-randomized, double-blind, controlled programmatic study in rural Niger. | | <https://pubmed.ncbi.nlm.nih.gov/17974365/> | | excluded by title and abstract | | |
|  | | Zack RM et al (2014) | Risk Factors for Preterm Birth among HIV-Infected Tanzanian Women: A Prospective Study. | | <https://pubmed.ncbi.nlm.nih.gov/25328529/> | | excluded by title and abstract | | |
|  | | Young N, et al (2018), | Integrated point-of-care testing (POCT) of HIV, syphilis, malaria and anaemia in antenatal clinics in western Kenya: A longitudinal implementation study. | | <https://pubmed.ncbi.nlm.nih.gov/30028852/> | | excluded by title and abstract | | |
|  | | Young, N. et al. (2022) | Cost-effectiveness of antenatal multiple micronutrients and balanced energy protein supplementation compared to iron and folic acid supplementation in India, Pakistan, Mali, and Tanzania: A dynamic microsimulation study | | <https://journals.plos.org/plosmedicine/article>? | | excluded by title and abstract | | |
|  | | Young MW et al. (2000) | The effectiveness of weekly iron supplementation in pregnant women of rural northern Malawi | | https://pubmed.ncbi.nlm.nih.gov/10842553/ | | excluded by title and abstract | | |
|  | | Yoseph, S. et al. (2021) | Prevalence of early postnatal-care service utilization and its associated factors among mothers in Hawassa Zuria district, Sidama regional state, Ethiopia: A cross-sectional study | | <https://pubmed.ncbi.nlm.nih.gov/33564651/> | | excluded by title and abstract | | |
|  | | Yisak H et al. (2020) | Prevalence and Associated Factors of Clinical Vitamin A Deficiency Among Pre-School Children 1-5 Years of Age in Rural Kebeles in Farta District, South Gondar Zone, Ethiopia: A Mixed Methods Study. | | <https://pubmed.ncbi.nlm.nih.gov/33116564/> | | excluded by title and abstract | | |
|  | | Yawson AE et al. (2017) | The lancet series nutritional interventions in Ghana: a determinants analysis approach to inform nutrition strategic planning. | | <https://pubmed.ncbi.nlm.nih.gov/32153809/> | | excluded by title and abstract | | |
|  | | Yatich NJ et al. (2010) | Malaria, intestinal helminths and other risk factors for stillbirth in Ghana. | | <https://pubmed.ncbi.nlm.nih.gov/20379355/> | | excluded by title and abstract | | |
|  | | Yakah W et al. (2019) | Serum Vitamin D is Differentially Associated with Socioemotional Adjustment in Early School-Aged Ugandan Children According to Perinatal HIV Status and In Utero/Peripartum Antiretroviral Exposure History. | | <https://pubmed.ncbi.nlm.nih.gov/31336843/> | | excluded by title and abstract | | |
|  | | Wuehler, S.E., Hess, S.Y., Brown, K.H. (2011) | Accelerating improvements in nutritional and health status of young children in the Sahel region of Sub-Saharan Africa: Review of international guidelines on infant and young child feeding and nutrition | | <https://www.scopus.com/inward/record.uri?eid=2-s2.0-79952713218&doi=10.1111%2fj.1740-8709.2010.00306.x&partnerID=40&md5=a503b7a18feae6393078a3fe5e3fb87c> | | excluded by title and abstract | | |
|  | | Workicho A et al. (2019) | Adolescent pregnancy and linear growth of infants: a birth cohort study in rural Ethiopia. | | <https://pubmed.ncbi.nlm.nih.gov/30940147/> | | excluded by title and abstract | | |
|  | | Wondmikun Y (2005) | Lipid-soluble antioxidants status and some of its socio-economic determinants among pregnant Ethiopians at the third trimester. | | <https://pubmed.ncbi.nlm.nih.gov/16236187/> | | excluded by title and abstract | | |
|  | | Wiysonge CS et al. (2005) | Vitamin A supplementation for reducing the risk of mother-to-child transmission of HIV infection. | | <https://pubmed.ncbi.nlm.nih.gov/16235332/> | | excluded by title and abstract | | |
|  | | Wiysonge CS et al. (2017) | Vitamin A supplements for reducing mother-to-child HIV transmission. | | <https://pubmed.ncbi.nlm.nih.gov/28880995/> | | excluded by title and abstract | | |
|  | | Wirth JP et al. (2022) | Risk factors of anaemia and iron deficiency in Somali children and women: Findings from the 2019 Somalia Micronutrient Survey. | | <https://pubmed.ncbi.nlm.nih.gov/34405549/> | | excluded by title and abstract | | |
|  | | Wirth JP et al. (2016) | Anemia, Micronutrient Deficiencies, and Malaria in Children and Women in Sierra Leone Prior to the Ebola Outbreak - Findings of a Cross-Sectional Study. | | <https://pubmed.ncbi.nlm.nih.gov/27163254/> | | excluded by title and abstract | | |
|  | | Winje BA et al. (2018) | Does early Vitamin B12 supplementation improve neurodevelopment and cognitive function in childhood and into school age: A study protocol for extended follow-ups from randomised controlled trials in India and Tanzania | | <https://www.bmj.com/content/383/bmj-2022-071725> | | excluded by title and abstract | | |
|  | | Williams IO, Essien EU, Eka OU (2011) | Socioeconomic factors and vitamin a status of pregnant women in Calabar urban, southeastern Nigeria. | | <https://pubmed.ncbi.nlm.nih.gov/19937371/> | | excluded by title and abstract | | |
|  | | Williams IO, Eka OU, Essien EU (2008) | Vitamin A status of pregnant women in Calabar metropolis, Nigeria. | | <https://pubmed.ncbi.nlm.nih.gov/18819622/> | | excluded by title and abstract | | |
|  | | Wessells KR (2017) | Micronutrient Status among Pregnant Women in Zinder, Niger and Risk Factors Associated with Deficiency. | | <https://pubmed.ncbi.nlm.nih.gov/28445440/> | | excluded by title and abstract | | |
|  | | Wemakor A, Ziyaaba A, Yiripuo F (2022) | Risk factors of anaemia among postpartum women in Bolgatanga Municipality, Ghana. | | <https://pubmed.ncbi.nlm.nih.gov/35751125/> | | excluded by title and abstract | | |
|  | | Welderufael AL et al (2019) | Nutritional status among women whose pregnancy outcome was afflicted with neural tube defects in Tigray region of Ethiopia | | <https://pubmed.ncbi.nlm.nih.gov/30651190/> | | excluded by title and abstract | | |
|  | | Weldegebriel SG et al (2023) | Maternal dietary pattern and its association with birthweight in Northern Ethiopia: A hospital-based cross-sectional study. | | <https://pubmed.ncbi.nlm.nih.gov/37457182/> | | excluded by title and abstract | | |
|  | | Wegmüller R, et al 2020 | Anemia, micronutrient deficiencies, malaria, hemoglobinopathies and malnutrition in young children and non-pregnant women in Ghana: Findings from a national survey. | | <https://pubmed.ncbi.nlm.nih.gov/31999737/> | | excluded by title and abstract | | |
|  | | Wang et al (2023) | he Effects of Prenatal and Postnatal Maternal High-Dose Vitamin B-12 Supplementation on Human Milk Vitamin B-12 in Tanzania | | <https://www.embase.com/search/results?subaction=viewrecord&id=L2026059745&from=export%20%20%20%20%20U2%20%20-%20L2026059745> | | excluded by title and abstract | | |
|  | | Wang D et al (2022) | ffects of prenatal and postnatal maternal multiple micronutrient supplementation on child growth and morbidity in Tanzania: a double-blind, randomized-controlled trial. | | <https://pubmed.ncbi.nlm.nih.gov/34151973/> | | excluded by title and abstract | | |
|  | | Wallace et al (2008) | Homocysteine concentration, related B vitamins, and betaine in pregnant women recruited to the Seychelles Child Development Study | | <https://www.embase.com/search/results?subaction=viewrecord&id=L351226076&from=export%20%20%20%20%20U2%20%20-%20L351226076> | | excluded by title and abstract | | |
|  | | Visser Me et al (2017) | Micronutrient supplementation in adults with HIV infection. | | <https://pubmed.ncbi.nlm.nih.gov/28518221/> | | excluded by title and abstract | | |
|  | | Villard L, Bates CJ (1986) | Dark adaptation in pregnant and lactating Gambian women: feasibility of measurement and relation to vitamin A status. | | <https://pubmed.ncbi.nlm.nih.gov/3771288/> | | excluded by title and abstract | | |
|  | | Villamor E et al (2005) | Vitamin supplementation of HIV-infected women improves postnatal child growth. | | <https://pubmed.ncbi.nlm.nih.gov/15817867/> | | excluded by title and abstract | | |
|  | | Villamor E et al. (2002) | Effect of multivitamin and vitamin A supplements on weight gain during pregnancy among HIV-1-infected women | | <https://pubmed.ncbi.nlm.nih.gov/12399282/> | | excluded by title and abstract | | |
|  | | Villamor, E., Kapiga, S.H., Fawzi, W.W. (2006) | Vitamin A serostatus and heterosexual transmission of HIV: Case-control study in Tanzania and review of the evidence | | <https://www.embase.com/search/results?subaction=viewrecord&id=L44367104&from=export%20%20%20%20%20U2%20%20-%20L44367104> | | excluded by title and abstract | | |
|  | | Verhoeff FH et al (1999) | Malaria in pregnancy and its consequences for the infant in rural Malawi. | | <https://pubmed.ncbi.nlm.nih.gov/10715686/> | | excluded by title and abstract | | |
|  | | Venkatesh PA et al. (2005) | Predictors of incident tuberculosis among HIV-1-infected women in Tanzania | | <https://pubmed.ncbi.nlm.nih.gov/16229221/> | | excluded by title and abstract | | |
|  | | Velaphi et al ()2019 | Maternal and neonatal vitamin D status at birth in black South Africans | | <https://www.embase.com/search/results?subaction=viewrecord&id=L629655112&from=export%20%20%20%20%20U2%20%20-%20L629655112> | | excluded by title and abstract | | |
|  | | Van Stuijvenberg M et al. (2013) | Implications of frequent liver consumption among pregnant women from a low socio-economic South African community | | https://www.embase.com/search/results?subaction=viewrecord&id=L71179622&from=export U2 - L71179622 | | excluded by title and abstract | | |
|  | | Vanslambrouck et al (2021) | Effect of balanced energy-protein supplementation during pregnancy and lactation on birth outcomes and infant growth in rural Burkina Faso: Study protocol for a randomised controlled trial | | <https://www.scopus.com/inward/record.uri?eid=2-s2.0-85103248431&doi=10.1136%2fbmjopen-2020-038393&partnerID=40&md5=1929d9bafb6333cd1144f6740929f2cc> | | excluded by title and abstract | | |
|  | | Vanié SC et al (2022) | Nutritional and Obstetric Determinant of Iron Deficiency Anemia among Pregnant Women Attending Antenatal Care Services in Public Health Hospitals in Abidjan (Côte d'Ivoire): A Cross-Sectional Study. | | <https://pubmed.ncbi.nlm.nih.gov/34672848/> | | excluded by title and abstract | | |
|  | | van Eijsden M, van der Wal MF, Bonsel GJ (2006) | Folic acid knowledge and use in a multi-ethnic pregnancy cohort: the role of language proficiency. | | <https://pubmed.ncbi.nlm.nih.gov/17081188/> | | excluded by title and abstract | | |
|  | | van Eijk AM et al (2008) | Plasma folate level and high-dose folate supplementation predict sulfadoxine-pyrimethamine treatment failure in pregnant women in Western kenya who have uncomplicated malaria. | | <https://pubmed.ncbi.nlm.nih.gov/18831691/> | | excluded by title and abstract | | |
|  | | Vanderjagt DJ, et al (2009) | Subclinical vitamin B12 deficiency in pregnant women attending an antenatal clinic in Nigeria. | | <https://pubmed.ncbi.nlm.nih.gov/19835494/> | | excluded by title and abstract | | |
|  | | Vanderjagt DJ, (2011) | Assessment of the vitamin B12 status of pregnant women in Nigeria using plasma holotranscobalamin. | | <https://pubmed.ncbi.nlm.nih.gov/21789284/> | | excluded by title and abstract | | |
|  | | Vanderjagt DJ et al (2004) | High-density lipoprotein and homocysteine levels correlate inversely in preeclamptic women in northern Nigeria. | | <https://pubmed.ncbi.nlm.nih.gov/15144334/> | | excluded by title and abstract | | |
|  | | Vanderjagt DJ et al (2007) | Nutritional factors associated with anaemia in pregnant women in northern Nigeria. | | <https://pubmed.ncbi.nlm.nih.gov/17615906/> | | excluded by title and abstract | | |
|  | | Van De Perre, P. (1999) | Transmission of human immunodeficiency virus type 1 through breastfeeding: How can it be prevented? | | <https://www.scopus.com/inward/record.uri?eid=2-s2.0-0032953964&doi=10.1086%2f314793&partnerID=40&md5=21f8616319cf2183fe6f2681eda6add1> | | excluded by title and abstract | | |
|  | | van den Broek NR(2006) | Randomised trial of vitamin A supplementation in pregnant women in rural Malawi found to be anaemic on screening by HemoCue. | | <https://pubmed.ncbi.nlm.nih.gov/16579803/> | | excluded by title and abstract | | |
|  | | van den Broek NR, Letsky EA (2000) | Etiology of anemia in pregnancy in south Malawi. | | <https://pubmed.ncbi.nlm.nih.gov/10871590/> | | excluded by title and abstract | | |
|  | | van den Broek N, Kulier R, Gülmezoglu AM, Villar J (2010) | Vitamin A supplementation during pregnancy. | | <https://pubmed.ncbi.nlm.nih.gov/21069669/> | | excluded by title and abstract | | |
|  | | van den Broek N (2010) | Vitamin A supplementation during pregnancy for maternal and newborn outcomes. | | <https://pubmed.ncbi.nlm.nih.gov/21069707/> | | excluded by title and abstract | | |
|  | | Van DE, Kulier R, Gülmezoglu AM, Villar J (2002) | Vitamin A supplementation during pregnancy. | | <https://pubmed.ncbi.nlm.nih.gov/12519564/> | | excluded by title and abstract | | |
|  | | Valadez JJ (2020) | Is development aid to strengthen health systems during protracted conflict a useful investment? The case of South Sudan, 2011-2015. | | <https://pubmed.ncbi.nlm.nih.gov/32377402/> | | excluded by title and abstract | | |
|  | | Urassa, D.P., Nystrom, L., Carlsted, A. (2011) | ffectiveness of routine antihelminthic treatment on anaemia in pregnancy in Rufiji District, Tanzania: a cluster randomised controlled trial. | | <https://www.embase.com/search/results?subaction=viewrecord&id=L366354971&from=export%20%20%20%20%20U2%20%20-%20L366354971> | | excluded by title and abstract | | |
|  | | Ukoha, 2022 | Current state of preconception care in sub-Saharan Africa: A systematic scoping review | |  | | excluded by title and abstract | | |
|  | | Ukoha WC, Mtshali NG (2022) | Preconception care practices among primary health care nurses working in public health facilities in KwaZulu-Natal. | | <https://pubmed.ncbi.nlm.nih.gov/36161863/> | | excluded by title and abstract | | |
|  | | Ugwu RO, Eneh AU, Oruamabo RS (2007) | Neural tube defects in a university teaching hospital in southern Nigeria: trends and outcome. | | <https://pubmed.ncbi.nlm.nih.gov/18080598/> | | excluded by title and abstract | | |
|  | | Ugwa E, Gwarzo M, Ashimi A (2015) | Oxidative stress and antioxidant status of pregnant rural women in north-west Nigeria: prospective cohort study. | | <https://pubmed.ncbi.nlm.nih.gov/24824107/> | | excluded by title and abstract | | |
|  | | Ugwa EA, Iwasam EA, Nwali MI (2013) | Low Serum Vitamin C Status Among Pregnant Women Attending Antenatal Care at General Hospital Dawakin Kudu, Northwest Nigeria. | | <https://pubmed.ncbi.nlm.nih.gov/27014432/> | | excluded by title and abstract | | |
|  | | Ugwa EA (2015) | Vitamins A and E Deficiencies among Pregnant Women Attending Antenatal Care at General Hospital Dawakin Kudu, North-West Nigeria | | <https://pubmed.ncbi.nlm.nih.gov/26288709/> | | excluded by title and abstract | | |
|  | | Ubbink et al (1999) | Folate status, homocysteine metabolism, and methylene tetrahydrofolate reductase genotype in rural South African blacks with a history of pregnancy complicated by neural tube defects | | <https://www.embase.com/search/results?subaction=viewrecord&id=L29072287&from=export%20%20%20%20%20U2%20%20-%20L29072287> | | excluded by title and abstract | | |
|  | | Tveden-Nyborg (2012) | Maternal vitamin C deficiency during pregnancy persistently impairs hippocampal neurogenesis in offspring of guinea pigs. | | <https://pubmed.ncbi.nlm.nih.gov/23119033/> | | excluded by title and abstract | | |
|  | | Turner et al (20160 | Serum vitamin D status and bacterial vaginosis prevalence and incidence in Zimbabwean women | | <https://www.embase.com/search/results?subaction=viewrecord&id=L609943477&from=export%20%20%20%20%20U2%20%20-%20L609943477> | | excluded by title and abstract | | |
|  | | Tukeman GL, Wei H, Finnell RH, Cabrera RM (2023) | Dolutegravir induced neural tube defects in mice are folate responsive. | | <https://pubmed.ncbi.nlm.nih.gov/37382903/> | | excluded by title and abstract | | |
|  | | Tuha A, Gurbie Y, Hailu HG (2019 ) | Evaluation of Knowledge and Practice of Pharmacy Professionals regarding the Risk of Medication Use during Pregnancy in Dessie Town, Northeast Ethiopia: A Cross-Sectional Study. | | <https://pubmed.ncbi.nlm.nih.gov/31428474/> | | excluded by title and abstract | | |
|  | | Tshibumbu DD, Blitz J (2016) | Modifiable antenatal risk factors for stillbirth amongst pregnant women in the Omusati region, Namibia. | | <https://pubmed.ncbi.nlm.nih.gov/27247156/> | | excluded by title and abstract | | |
|  | | Tsehay B (2019) | Determinants and seasonality of major structural birth defects among newborns delivered at primary and referral hospital of East and West Gojjam zones, Northwest Ethiopia 2017-2018: case-control study. | | <https://pubmed.ncbi.nlm.nih.gov/31399144/> | | excluded by title and abstract | | |
|  | | Tricarico JM, Kebreab E, Wattiaux MA (2020) | MILK Symposium review: Sustainability of dairy production and consumption in low-income countries with emphasis on productivity and environmental impact. | | <https://pubmed.ncbi.nlm.nih.gov/33076189/> | | excluded by title and abstract | | |
|  | | Torlesse, H., Hodges, M. (2001) | Albendazole therapy and reduced decline in haemoglobin concentration during pregnancy (Sierra Leone) | | <https://www.embase.com/search/results?subaction=viewrecord&id=L34947387&from=export%20%20%20%20%20U2%20%20-%20L34947387> | | excluded by title and abstract | | |
|  | | Torlesse H, Hodges M (2000) | Anthelminthic treatment and haemoglobin concentrations during pregnancy | | <https://pubmed.ncbi.nlm.nih.gov/11355560/> | | excluded by title and abstract | | |
|  | | Tompolski C, Tynecki J (1971) | Effect of folic acid deficiency on pregnancy in guinea pigs | | <https://pubmed.ncbi.nlm.nih.gov/5581641/> | | excluded by title and abstract | | |
|  | | Tomkins, A. (2001) | Nutrition and maternal morbidity and mortality | | <https://www.embase.com/search/results?subaction=viewrecord&id=L32586477&from=export%20%20%20%20%20U2%20%20-%20L32586477> | | excluded by title and abstract | | |
|  | | Toko et al (2015) | Vitamin D Deficiency Increases the Risk of Stunting and Other Adverse Pregnancy Outcomes: Results from a Perinatal Cohort Study in Kenya | | <https://go.exlibris.link/zWv1NcJ2> | | excluded by title and abstract | | |
|  | | Toko EN (2016) | Maternal Vitamin D Status and Adverse Birth Outcomes in Children from Rural Western Kenya. | | <https://pubmed.ncbi.nlm.nih.gov/27941597/> | | excluded by title and abstract | | |
|  | | Tita AT et al (2005) | Evidence-based reproductive health care in Cameroon: population-based study of awareness, use and barriers. | | <https://pubmed.ncbi.nlm.nih.gov/16462981/> | | excluded by title and abstract | | |
|  | | Tindall AM et al (2020) | Vitamin D status, nutrition and growth in HIV-infected mothers and HIV-exposed infants and children in Botswana. | | <https://pubmed.ncbi.nlm.nih.gov/32790765/> | | excluded by title and abstract | | |
|  | | Tinago et al (2017) | Individual and structural environmental influences on utilization of iron and folic acid supplementation among pregnant women in Harare, Zimbabwe | | <https://www.embase.com/search/results?subaction=viewrecord&id=L611598102&from=export%20%20%20%20%20U2%20%20-%20L611598102> | | excluded by title and abstract | | |
|  | | Tikuye HH, Gebremedhin S, Mesfin A, Whiting S (2019) | Prevalence and Factors Associated with Undernutrition among Exclusively Breastfeeding Women in Arba Minch Zuria District, Southern Ethiopia: A Cross-sectional Community-Based Study. | | <https://pubmed.ncbi.nlm.nih.gov/30700959/> | | excluded by title and abstract | | |
|  | | Thwing JI, et al (2011) | Success of Senegal's first nationwide distribution of long-lasting insecticide-treated nets to children under five - contribution toward universal coverage. | | <https://pubmed.ncbi.nlm.nih.gov/21489278/> | | excluded by title and abstract | | |
|  | | Thomson J (1997) | Anaemia in pregnant women in eastern Caprivi, Namibia. | | <https://pubmed.ncbi.nlm.nih.gov/9472280/> | | excluded by title and abstract | | |
|  | | Thaler et al (2006) | Effects of riboflavin given to pregnant women on the incidence of malaria: Results of a prospective randomized double blind study | | <https://www.scopus.com/inward/record.uri?eid=2-s2.0-33646471396&doi=10.1055%2fs-2006-924038&partnerID=40&md5=de3debdbe9537b8ec895adef93fb3edf> | | excluded by title and abstract | | |
|  | | Tesfay N, et al (2023) | Birth prevalence and risk factors of neural tube defects in Ethiopia: a systematic review and meta-analysis. | | <https://pubmed.ncbi.nlm.nih.gov/37940152/> | | excluded by title and abstract | | |
|  | | Tesfa E et al., 2023 | Determinants of pre-eclampsia among pregnant women attending antenatal care and delivery services at Bahir Dar public hospitals, northwest Ethiopia: A case-control study | | <https://pubmed.ncbi.nlm.nih.gov/37519426/> | | excluded by title and abstract | | |
|  | | Tefera, et al (2022) | Red blood cell folate level and associated factors of folate insufficiency among pregnant women attending antenatal care during their first trimester of pregnancy in Addis Ababa, Ethiopia | | 10.1177/20503121221118987 | | excluded by title and abstract | | |
|  | | Taye M et al (2018) | Factors associated with congenital anomalies in Addis Ababa and the Amhara Region, Ethiopia: a case-control study. | | <https://pubmed.ncbi.nlm.nih.gov/29699508/> | | excluded by title and abstract | | |
|  | | Taye M et al (2019) | Congenital anomalies prevalence in Addis Ababa and the Amhara region, Ethiopia: a descriptive cross-sectional study. | | https://pubmed.ncbi.nlm.nih.gov/31296186/ | | excluded by title and abstract | | |
|  | | Tatala et al(2002) | Effect of micronutrient fortified beverage on nutritional anaemia during pregnancy. | | <https://www.embase.com/search/results?subaction=viewrecord&id=L36494538&from=export%20%20%20%20%20U2%20%20-%20L36494538> | | excluded by title and abstract | | |
|  | | Tanumihardjo et al (2015) | Hypercarotenodermia in Zambia: Which children turned orange during mango season? | | <https://www.embase.com/search/results?subaction=viewrecord&id=L605893662&from=export%20%20%20%20%20U2%20%20-%20L605893662> | | excluded by title and abstract | | |
|  | | Tamirat KS et al (2022) | Geographical variations and determinants of iron and folic acid supplementation during pregnancy in Ethiopia: analysis of 2019 mini demographic and health survey. | | <https://pubmed.ncbi.nlm.nih.gov/35168542/> | | excluded by title and abstract | | |
|  | | Taha, T.E. (2011) | Mother-to-child transmission of HIV-1 in sub-Saharan Africa: Past, present and future challenges | | <https://www.scopus.com/inward/record.uri?eid=2-s2.0-79956219438&doi=10.1016%2fj.lfs.2010.09.031&partnerID=40&md5=ffb5838309087dc216c604cdae64c47e> | | excluded by title and abstract | | |
|  | | adesse T, Abebe M, Molla W, Ahmed Mahamed A, Mebratu A, (2023) | Magnitude and associated factors of low birth weight among term newborns delivered in Addis Ababa public hospitals, Ethiopia, 2021 | | <https://pubmed.ncbi.nlm.nih.gov/36037087/> | | excluded by title and abstract | | |
|  | | Tadesse L, Tafesse F, Hamamy H, (2014) | Communities and community genetics in Ethiopia | | <https://pubmed.ncbi.nlm.nih.gov/25404975/> | | excluded by title and abstract | | |
|  | | adesse AW, Kassa AM, Aychiluhm SB, (2020) | Determinants of Neural Tube Defects among Newborns in Amhara Region, Ethiopia: A Case-Control Study | | <https://pubmed.ncbi.nlm.nih.gov/33193764/> | | excluded by title and abstract | | |
|  | | Tabatabaei et al., (2014) | High vitamin D status before conception, but not during pregnancy, is inversely associated with maternal gestational diabetes mellitus in guinea pigs | | <https://pubmed.ncbi.nlm.nih.gov/25342700/> | | excluded by title and abstract | | |
|  | | abatabaei et al., (2014) | Dietary vitamin D during pregnancy has dose-dependent effects on long bone density and architecture in guinea pig offspring but not the sows | | <https://pubmed.ncbi.nlm.nih.gov/25320192/> | | excluded by title and abstract | | |
|  | | Swareldhab ESE et al (2021) | Assessment of Micronutrient Situation among Reproductive-Age Women (15-49) and Under-Five Children in Sudan | | <https://pubmed.ncbi.nlm.nih.gov/34444943/> | | excluded by title and abstract | | |
|  | | Svigel SS et al (2021) | Low prevalence of highly sulfadoxine-resistant dihydropteroate synthase alleles in Plasmodium falciparum isolates in Benin | | https://pubmed.ncbi.nlm.nih.gov/33546703/ | | excluded by title and abstract | | |
|  | | Sudfeld CR et al (2022) | Vitamin D3 supplementation during pregnancy and lactation for women living with HIV in Tanzania: A randomized controlled trial | | <https://www.embase.com/search/results?subaction=viewrecord&id=L2017880009&from=export> | | excluded by title and abstract | | |
|  | | Sudfeld CR et al (2017) | Effect of maternal vitamin D3 supplementation on maternal health, birth outcomes, and infant growth among HIV-infected Tanzanian pregnant women: Study protocol for a randomized controlled trial | | <https://www.embase.com/search/results?subaction=viewrecord&id=L618102106&from=export> | | excluded by title and abstract | | |
|  | | Sudfeld CR et al (2022) | Evaluation of multiple micronutrient supplementation and medium-quantity lipid-based nutrient supplementation in pregnancy on child development in rural Niger: A secondary analysis of a cluster randomized controlled trial | | <https://pubmed.ncbi.nlm.nih.gov/35500028/> | | excluded by title and abstract | | |
|  | | türchler D et al (1983) | The influence of schistosomiasis on the serum concentrations of retinol and retinol binding protein of a rural population in Liberia. | | <https://pubmed.ncbi.nlm.nih.gov/6138976/> | | excluded by title and abstract | | |
|  | | Strain JJ et al (2020) | Nutrition and neurodevelopment: the search for candidate nutrients in the Seychelles Child Development Nutrition Study. | | <https://pubmed.ncbi.nlm.nih.gov/33741113/> | | excluded by title and abstract | | |
|  | | Stojanov S (1963) | EFFECT OF VITAMIN C ON PREGNANCY. | | <https://pubmed.ncbi.nlm.nih.gov/1409720> | | excluded by title and abstract | | |
|  | | Stewart et al. (2017) | The impact of maternal diet fortification with lipid-based nutrient supplements on postpartum depression in rural Malawi: a randomized-controlled trial. | | <https://pubmed.ncbi.nlm.nih.gov/27060705/> | | excluded by title and abstract | | |
|  | | Stewart et al. (2020) | Lipid-Based Nutrient Supplementation Reduces Child Anemia and Increases Micronutrient Status in Madagascar | | <https://pubmed.ncbi.nlm.nih.gov/32006028/> | | excluded by title and abstract | | |
|  | | Stewart et al. (2015) | Maternal Cortisol and Stress are Associated with Birth Outcomes, but are Not Affected by Lipid-Based Nutrient Supplements | | <https://pubmed.ncbi.nlm.nih.gov/2669464> | | excluded by title and abstract | | |
|  | | Stewart et al., 2019 | Effects of lipid-based nutrient supplements and infant and young child feeding counseling with or without improved water, sanitation, and hygiene (WASH) on anemia and micronutrient status: results from 2 cluster-randomized trials in Kenya and Bangladesh | | <https://pubmed.ncbi.nlm.nih.gov/30624600/> | | excluded by title and abstract | | |
|  | | Stein et al., 1986 | Pooled pasteurized breast milk and untreated own mother's milk in the feeding of very low birth weight babies: a randomized controlled trial | | <https://pubmed.ncbi.nlm.nih.gov/3514832/> | | excluded by title and abstract | | |
|  | | Stevens & Metz, 1964 | The absorption of folic acid in megaloblastic anaemia associated with pregnancy | | <https://pubmed.ncbi.nlm.nih.gov/14217009/> | | excluded by title and abstract | | |
|  | | Stein et al., 1986 | Pooled pasteurized breast milk and untreated own mother's milk in the feeding of very low birth weight babies: a randomized controlled trial | | <https://pubmed.ncbi.nlm.nih.gov/3514832/> | | excluded by title and abstract | | |
|  | | Ssentongo et al., 2022 | Birth prevalence of neural tube defects in eastern Africa: a systematic review and meta-analysis | | <https://pubmed.ncbi.nlm.nih.gov/35650541/> | | excluded by title and abstract | | |
|  | | Sridaran et al., (2010) | Anti-folate drug resistance in Africa: meta-analysis of reported dihydrofolate reductase (dhfr) and dihydropteroate synthase (dhps) mutant genotype frequencies in African Plasmodium falciparum parasite populations | | <https://pubmed.ncbi.nlm.nih.gov/20799995/> | | excluded by title and abstract | | |
|  | | Sonuga AA, Sonuga OO, (2020) | Hypovitaminosis D Is Associated with Some Metabolic Indices in Gestational Diabetes Mellitus | | <https://pubmed.ncbi.nlm.nih.gov/32884931/> | | excluded by title and abstract | | |
|  | | Some ER, Meda N, (2014) | Does the national program of prevention of mother to child transmission of HIV (PMTCT) reach its target in Ouagadougou, Burkina Faso? | | <https://www.scopus.com/inward/record.uri?eid=2-s2.0-84921664203&doi=10.4314%2fahs.v14i4.17&partnerID=40&md5=2fd4fa0f7965824808e8f60d606f88bf> | | excluded by title and abstract | | |
|  | | Sodde et al., 2023 | Magnitude and predictors of anemia among preschool children (36-59 months) in Atingo town, Jimma, Ethiopia. | | https://pubmed.ncbi.nlm.nih.gov/37334043/ | | excluded by title and abstract | | |
|  | | Smith et al., 2007 | Multivitamin supplementation in HIV-positive pregnant women: impact on depression and quality of life in a resource-poor setting. | | https://pubmed.ncbi.nlm.nih.gov/17461847/ | | excluded by title and abstract | | |
|  | |  |  | |  | | excluded by title and abstract | | |
|  | | Smith et al., (2007) | Malaria prevention during pregnancy: Assessing the disease burden one year after implementing a program of intermittent preventive treatment in Koupéla District, Burkina Faso | | <https://www.scopus.com/inward/record.uri?eid=2-s2.0-33748740753&doi=10.4269%2fajtmh.2006.75.205&partnerID=40&md5=6b11606bf42dae7fd9c0fa4b94d4f0b> | | excluded by title and abstract | | |
|  | | Sletner et al., (2010) | Levels of 25-OH-vitamin D in early pregnancy in women from five ethnic groups with and without gestational diabetes | | <https://www.embase.com/search/results?subaction=viewrecord&id=L70262395&from=export> | | excluded by title and abstract | | |
|  | | Singh et al., 2015 | Foetal congenital anomalies: An experience from a tertiary health institution in north-west Nigeria (2011-2013) | | <https://pubmed.ncbi.nlm.nih.gov/26739205/> | | excluded by title and abstract | | |
|  | | Silesh et al., 2021 | Prevalence and Trends of Congenital Anomalies Among Neonates at Jimma Medical Center, Jimma, Ethiopia: A Three-Year Retrospective Study | | <https://pubmed.ncbi.nlm.nih.gov/33628075/> | | excluded by title and abstract | | |
|  | | Siekmans et al., 2018 | Barriers and enablers for iron folic acid (IFA) supplementation in pregnant women | | <https://www.embase.com/search/results?subaction=viewrecord&id=L619934184&from=export> | | excluded by title and abstract | | |
|  | | Siegrist et al., 1992 | Schistosoma haematobium infection in pregnancy | | <https://pubmed.ncbi.nlm.nih.gov/1356302/> | | excluded by title and abstract | | |
|  | | Siekmans et al., 2011 (updated 2018) | Micronutrient supplementation in pregnant women with HIV infection | | <https://doi.org/10.1111/mcn.12493> | | excluded by title and abstract | | |
|  | | Shulman CE, 1999 | Malaria in pregnancy: its relevance to safe-motherhood programmes | | <https://pubmed.ncbi.nlm.nih.gov/10715689/> | | excluded by title and abstract | | |
|  | | Shapiro J et al., 1965 | Folate and Vitamin B-12 Deficiency Associated with Lactation | | <https://pubmed.ncbi.nlm.nih.gov/14317444/> | | excluded by title and abstract | | |
|  | | Sendeku FW et al., 2020 | Adherence to iron-folic acid supplementation among pregnant women in Ethiopia: a systematic review and meta-analysis | | <https://pubmed.ncbi.nlm.nih.gov/32131751/> | | excluded by title and abstract | | |
|  | | Semba et al., 1994 | Maternal Vitamin A Deficiency and Mother-to-Child Transmission of HIV-1 | | <https://pubmed.ncbi.nlm.nih.gov/7911919/> | | excluded by title and abstract | | |
|  | | Semba et al., 1995 | Infant Mortality and Maternal Vitamin A Deficiency During HIV Infection | | <https://pubmed.ncbi.nlm.nih.gov/8645848/> | | excluded by title and abstract | | |
|  | | Semba et al., 1998 | Maternal Vitamin A Deficiency and Infant Mortality in HIV-Negative Women | | <https://pubmed.ncbi.nlm.nih.gov/9718911/> | | excluded by title and abstract | | |
|  | | Semba RD (1997) | Maternal vitamin A deficiency and child growth failure during human immunodeficiency virus infection. | | <https://pubmed.ncbi.nlm.nih.gov/9117453/> | | excluded by title and abstract | | |
|  | | Semba RD et al (2000) | Plasma and breast milk vitamin A as indicators of vitamin A status in pregnant women. | | <https://pubmed.ncbi.nlm.nih.gov/11214351/> | | excluded by title and abstract | | |
|  | | Semba RD et al (2001) | Impact of vitamin A supplementation on anaemia and plasma erythropoietin concentrations in pregnant women: a controlled clinical trial. | | <https://pubmed.ncbi.nlm.nih.gov/11488938/> | | excluded by title and abstract | | |
|  | | Sellen, D. (2018) | Maternal health services utilization prior to enrich program interventions in Asia and Africa | | <https://www.embase.com/search/results?subaction=viewrecord&id=L624607159&from=export%20%20%20%20%20U2%20%20-%20L624607159> | | excluded by title and abstract | | |
|  | | Seifu CN, (2018) | Association of husbands' education status with unintended pregnancy in their wives in southern Ethiopia: A cross-sectional study. | | <https://pubmed.ncbi.nlm.nih.gov/32645075/> | | excluded by title and abstract | | |
|  | | Seck, B.C., Jackson, R.T. (2009) | Providing iron/folic acid tablets free of charge improves compliance in pregnant women in Senegal | | https://www.embase.com/search/results?subaction=viewrecord&id=L50415717&from=export U2 - L50415717 | | excluded by title and abstract | | |
|  | | Schoeman, et al (2018) | Serum retinol in newborns and mothers in a Northern Cape area with a high liver consumption | | <https://www.embase.com/search/results?subaction=viewrecord&id=L71007479&from=export%20%20%20%20%20U2%20%20-%20L71007479> | | excluded by title and abstract | | |
|  | | Schjoldager JG et al (2015) | Erratum to: Maternal vitamin C deficiency during pregnancy results in transient fetal and placental growth retardation in guinea pigs. | | <https://pubmed.ncbi.nlm.nih.gov/25930964/> | | excluded by title and abstract | | |
|  | | Schjoldager et al (2013) | Prolonged maternal vitamin C deficiency overrides preferential fetal ascorbate transport but does not influence perinatal survival in guinea pigs | | <https://go.exlibris.link/Z3zTc80t> | | excluded by title and abstract | | |
|  | | Sartorius et al (2013) | Maternal anaemia and duration of zidovudine in antiretroviral regimens for preventing mother-to-child transmission: A randomized trial in three African countries | | <https://www.scopus.com/inward/record.uri?eid=2-s2.0-84887027905&doi=10.1186%2f1471-2334-13-522&partnerID=40&md5=e1982e62664eb3939c2be1c43a0017a6> | | excluded by title and abstract | | |
|  | | Saronga NJ et al (2019) | mHealth interventions targeting pregnancy intakes in low and lower-middle income countries: Systematic review. | | <https://pubmed.ncbi.nlm.nih.gov/30609297/> | | excluded by title and abstract | | |
|  | | Saronga et al (2020) | Nutrition services offered to pregnant women attending antenatal clinics in Dar es Salaam, Tanzania: A qualitative study | | https://www.embase.com/search/results?subaction=viewrecord&id=L632196665&from=export U2 - L632196665 | | excluded by title and abstract | | |
|  | | Saronga et al (2022) | Contents of nutrition care services among pregnant women attending antenatal clinic: An Exit interview | | https://www.embase.com/search/results?subaction=viewrecord&id=L635021484&from=export U2 - L635021484 | | excluded by title and abstract | | |
|  | | Sardasht et al (2020) | Preconception Care Utilization and Its Predictors in Women Referring to Teaching Hospitals in Mashhad: Preconception care utilization and Its Predictors | |  | | excluded by title and abstract | | |
|  | | Santos et al (2017) | Infant hydrocephalus in sub-Saharan Africa: The reality on the Tanzanian side of the lake | | https://www.embase.com/search/results?subaction=viewrecord&id=L619095809&from=export U2 - L619095809 | | excluded by title and abstract | | |
|  | | Sanghvi et al (2022) | Gaps in the implementation and uptake of maternal nutrition interventions in antenatal care services in Bangladesh, Burkina Faso, Ethiopia and India | | <https://www.scopus.com/inward/record.uri?eid=2-s2.0-85119681143&doi=10.1111%2fmcn.13293&partnerID=40&md5=91d465af3dd1bfa9b9d5bd462de9e980> | | excluded by title and abstract | | |
|  | | Sanghvi TG et al (2023) | Comprehensive Approach for Improving Adherence to Prenatal Iron and Folic Acid Supplements Based on Intervention Studies in Bangladesh, Burkina Faso, Ethiopia, and India. | | <https://pubmed.ncbi.nlm.nih.gov/37309106/> | | excluded by title and abstract | | |
|  | | Sanchez PA et al (1997) | Calcium and vitamin D status of pregnant teenagers in Maiduguri, Nigeria. | | <https://pubmed.ncbi.nlm.nih.gov/9433060/> | | excluded by title and abstract | | |
|  | | Samuel S et al (2020) | Socio-economic and dietary diversity characteristics are associated with anemia among pregnant women attending antenatal care services in public health centers of Kembata Tembaro Zone, Southern Ethiopia. | | <https://pubmed.ncbi.nlm.nih.gov/32328264/> | | excluded by title and abstract | | |
|  | | Salenius M et al (2022) | Association between prenatal provision of lipid-based nutrient supplements and caesarean delivery: Findings from a randomised controlled trial in Malawi. | | <https://pubmed.ncbi.nlm.nih.gov/35909334/> | | excluded by title and abstract | | |
|  | | Sako et al., 2016 | Cholera in pregnant women: the 2012 epidemic at the reference center at the Donka National Hospital in Conakry | | <https://pubmed.ncbi.nlm.nih.gov/2741297> | | excluded by title and abstract | | |
|  | | Sablah et al., 2012 | Thriving public-private partnership to fortify cooking oil in the West African Economic and Monetary Union (UEMOA) to control vitamin A deficiency: Faire Tache d'Huile en Afrique de l'Ouest | | https://pubmed.ncbi.nlm.nih.gov/23444712/ | | excluded by title and abstract | | |
|  | | Saaka et al., 2009 | Effect of joint iron and zinc supplementation on malarial infection and anaemia | | <https://pubmed.ncbi.nlm.nih.gov/20000066/> | | excluded by title and abstract | | |
|  | | Saaka et al., 2012 | Combined iron and zinc supplementation improves haematologic status of pregnant women in Upper West Region of Ghana | | <https://pubmed.ncbi.nlm.nih.gov/2366184> | | excluded by title and abstract | | |
|  | | Ryder RW, Behets F, 1994 | Reasons for the wide variation in reported rates of mother-to-child transmission of HIV-1 | | <https://pubmed.ncbi.nlm.nih.gov/781882> | | excluded by title and abstract | | |
|  | | Rutherford ME, Dockerty JD, Jasseh M, et al., 2009 | Investigating the relationship between child mortality and common preventive interventions in The Gambia | | <https://pubmed.ncbi.nlm.nih.gov/1917101> | | excluded by title and abstract | | |
|  | | ummens K, Van Herck E, van Bree R, et al., 2000 | Dietary calcium and phosphate restriction in guinea-pigs during pregnancy: fetal mineralization induces maternal hypocalcaemia despite increased 1 alpha,25-dihydroxycholecalciferol concentrations. | | <https://pubmed.ncbi.nlm.nih.gov/11103220> | | excluded by title and abstract | | |
|  | | Rummens K, van Bree R, Van Herck E, et al., 2002 | Vitamin D deficiency in guinea pigs: exacerbation of bone phenotype during pregnancy and disturbed fetal mineralization, with recovery by 1,25(OH)2D3 infusion or dietary calcium-phosphate supplementation. | | <https://pubmed.ncbi.nlm.nih.gov/12196905/> | | excluded by title and abstract | | |
|  | | uhotina et al., 2016 | An innovative approach to rural antenatal care: A report of a mobile clinic network in northern Tanzania | | <https://www.embase.com/search/results?subaction=viewrecord&id=L614044834&from=export> | | excluded by title and abstract | | |
|  | | Rougemont et al., 1977 | Malaria and anemia of pregnancy in an African savanna zone. Epidemiological, hematological, biological and immunological study of 2 villages of the Bamako region, Republic of Mali | | <https://pubmed.ncbi.nlm.nih.gov/580910/> | | excluded by title and abstract | | |
|  | | Rouamba et al., 2018 | Safety Profile of Drug Use During Pregnancy at Peripheral Health Centres in Burkina Faso: A Prospective Observational Cohort Study | | <https://www.scopus.com/inward/record.uri?eid=2-s2.0-85052751938&doi=10.1007%2fs40801-018-0141-1&partnerID=40&md5=2ea995f751fd7bbcf498a6d451bef19a> | | excluded by title and abstract | | |
|  | | Ross, 2002 | Recommendations for vitamin A supplementation. | | <https://pubmed.ncbi.nlm.nih.gov/12221268/> | | excluded by title and abstract | | |
|  | | Ronsmans et al., 2009 | Multiple micronutrient supplementation during pregnancy in low-income countries: a meta-analysis of effects on stillbirths and on early and late neonatal mortality. | | <https://pubmed.ncbi.nlm.nih.gov/20120796/> | | excluded by title and abstract | | |
|  | | Rolfe, 1994 | Beri-beri: "Endemic amongst urban Gambians". | | <https://pubmed.ncbi.nlm.nih.gov/12318772/> | | excluded by title and abstract | | |
|  | | Rohner et al., 2014 | Prevalence and public health relevance of micronutrient deficiencies and undernutrition in pre-school children and women of reproductive age in Côte d'Ivoire, West Africa. | | <https://pubmed.ncbi.nlm.nih.gov/24171836/> | | excluded by title and abstract | | |
|  | | Roble AK, et al (2023) | Determinants of Adverse Birth Outcomes in Public Hospitals of the Somali Region, Eastern Ethiopia: A Multicenter Unmatched Case-Control Study | | <https://pubmed.ncbi.nlm.nih.gov/37641685/> | | excluded by title and abstract | | |
|  | | Roberfroid, et al (2012) | Impact of prenatal multiple micronutrients on survival and growth during infancy: A randomized controlled trial | | <https://www.scopus.com/inward/record.uri?eid=2-s2.0-84859067815&doi=10.3945%2fajcn.111.029033&partnerID=40&md5=ff092719355c47416a0d19517d29361c> | | excluded by title and abstract | | |
|  | | Roberfroid, et al (2009) | Effects of maternal multiple micronutrient supplementation on fetal growth: A double-blind randomized controlled trial in rural Burkina Faso | | <https://www.scopus.com/inward/record.uri?eid=2-s2.0-55849136873&doi=10.3945%2fajcn.2008.26296&partnerID=40&md5=74f7c08a17c73c74a9e3e4> | | excluded by title and abstract | | |
|  | | Roberfroid, D. et al., 2010 | Effect of maternal multiple micronutrient supplements on cord blood hormones: A randomized controlled trial | | <https://www.scopus.com/inward/record.uri?eid=2-s2.0-77953047805&doi=10.3945%2fajcn.2009.28855&partnerID=40&md5=0104fa09ba8a3447038d3289205eaf11> | | excluded by title and abstract | | |
|  | | Roberfroid, D. et al., 2012 | Prenatal micronutrient supplements cumulatively increase fetal growth. | | https://pubmed.ncbi.nlm.nih.gov/22298571/ | | excluded by title and abstract | | |
|  | | Roberfroid, D. et al., 2011 | Randomized controlled trial of 2 prenatal iron supplements: Is there a dose-response relation with maternal hemoglobin? | | <https://www.scopus.com/inward/record.uri?eid=2-s2.0-79955422387&doi=10.3945%2fajcn.110.006239&partnerID=40&md5=ccb785ff931cacf6e671d5345330796b> | | excluded by title and abstract | | |
|  | | obb, L. et al., 2021 | Diet quality and associations with choline intake in pregnant women in Bloemfontein, South Africa | | https://www.embase.com/search/results?subaction=viewrecord&id=L2015774995&from=export | | excluded by title and abstract | | |
|  | | ighetti AA et al., 2012 | Etiology of anemia among infants, school-aged children, and young non-pregnant women in different settings of South-Central Cote d'Ivoire | | <https://pubmed.ncbi.nlm.nih.gov/22848097/> | | excluded by title and abstract | | |
|  | | Righetti AA et al., 2012 | Interactions and potential implications of Plasmodium falciparum-hookworm coinfection in different age groups in south-central Côte d'Ivoire | | <https://pubmed.ncbi.nlm.nih.gov/2313369> | | excluded by title and abstract | | |
|  | | Reinhardt WO, 1964 | Some factors influencing the thoracic-duct output of lymphocytes | | <https://pubmed.ncbi.nlm.nih.gov/14120530/> | | excluded by title and abstract | | |
|  | | Regan M et al., 2023 | Antenatal depression and adverse birth outcomes among pregnant women living with HIV in Dar es Salaam, Tanzania | | <https://pubmed.ncbi.nlm.nih.gov/37437720/> | | excluded by title and abstract | | |
|  | | Reddy VA et al., 1987 | Riboflavin, folate and vitamin C status of Gambian women during pregnancy: a comparison between urban and rural communities | | <https://pubmed.ncbi.nlm.nih.gov/3503405/> | | excluded by title and abstract | | |
|  | | Rebnord T et al., 2017 | Time trends in management of HIV-positive pregnant women in Northern Tanzania: A registry-based study | | https://pubmed.ncbi.nlm.nih.gov/28957345/ | | excluded by title and abstract | | |
|  | | Rayis DA et al., 2023 | Early pregnancy serum levels of folate and vitamin B12 in overweight and obese women in Khartoum, Sudan | | <https://www.embase.com/search/results?subaction=viewrecord&id=L639232615&from=export> | | excluded by title and abstract | | |
|  | | Ravenhall M et al., 2016 | Characterizing the impact of sustained sulfadoxine/pyrimethamine use upon the Plasmodium falciparum population in Malawi | | <https://pubmed.ncbi.nlm.nih.gov/27899115/> | | excluded by title and abstract | | |
|  | | Rebnord T et al., 2017 | Time trends in management of HIV-positive pregnant women in Northern Tanzania: A registry-based study | | <https://pubmed.ncbi.nlm.nih.gov/28957345/> | | excluded by title and abstract | | |
|  | | Prentice, A., 2013 | Nutritional rickets around the world | | https://www.embase.com/search/results?subaction=viewrecord&id=L52370110&from=export | | excluded by title and abstract | | |
|  | | Prado EL et al., 2016 | Effects of maternal and child lipid-based nutrient supplements on infant development: a randomized trial in Malawi | | https://pubmed.ncbi.nlm.nih.gov/673894010/ | | excluded by title and abstract | | |
|  | | Prado EL et al., 2018 | Associations of maternal nutrition during pregnancy and post-partum with maternal cognition and caregiving | | https://pubmed.ncbi.nlm.nih.gov/29098783/ | | excluded by title and abstract | | |
|  | | Prado EL et al., 2016 | Effects of pre- and post-natal lipid-based nutrient supplements on infant development in a randomized trial in Ghana | | <https://pubmed.ncbi.nlm.nih.gov/27391572/> | | excluded by title and abstract | | |
|  | | Prado EL et al., 2023 | Prenatal and postnatal small-quantity lipid-based nutrient supplements and children's social-emotional difficulties at ages 9-11 y in Ghana: follow-up of a randomized controlled trial | | <https://pubmed.ncbi.nlm.nih.gov/37257564/> | | excluded by title and abstract | | |
|  | | Powis K et al., 2014 | Vitamin D insufficiency in HIV-infected pregnant women receiving antiretroviral therapy is not associated with morbidity, mortality or growth impairment in their uninfected infants in Botswana | | <https://pubmed.ncbi.nlm.nih.gov/25037041/> | | excluded by title and abstract | | |
|  | | Pitchik H et al., 2017 | Prenatal nutrition, stimulation, and exposure to punishment are associated with early child motor, cognitive, and socioemotional development in Dar es Salaam, Tanzania | | <https://www.embase.com/search/results?subaction=viewrecord&id=L619278098&from=export> | | excluded by title and abstract | | |
|  | | Pham K et al., 2016 | The use of a lot quality assurance sampling methodology to assess and manage primary health interventions in conflict-affected West Darfur, Sudan | | <https://pubmed.ncbi.nlm.nih.gov/27757070/> | | excluded by title and abstract | | |
|  | | Petry N et al., 2021 | Risk factors for anaemia among Ghanaian women and children vary by population group and climate zone | | <https://pubmed.ncbi.nlm.nih.gov/32945623/> | | excluded by title and abstract | | |
|  | | Petry N et al., 2019 | Micronutrient Deficiencies, Nutritional Status and the Determinants of Anemia in Children 0-59 Months of Age and Non-Pregnant Women of Reproductive Age in The Gambia | | <https://pubmed.ncbi.nlm.nih.gov/31547543/> | | excluded by title and abstract | | |
|  | | Perumal N et al., 2022 | School readiness among children born to women living with HIV in Dar es Salaam, Tanzania: a cohort study protocol | | <https://pubmed.ncbi.nlm.nih.gov/36645758/> | | excluded by title and abstract | | |
|  | | Perry H et al., 1996 | Dietary habits, pregnancy weight gain and birthweights in a highland population of Kenya | | <https://pubmed.ncbi.nlm.nih.gov/8918001/> | | excluded by title and abstract | | |
|  | | Pereira GFM et al., 2021 | Dolutegravir and pregnancy outcomes in women on antiretroviral therapy in Brazil: a retrospective national cohort study | | <https://pubmed.ncbi.nlm.nih.gov/33387477/> | | excluded by title and abstract | | |
|  | | Peña-Rosas JP et al., 2019 | Fortification of rice with vitamins and minerals for addressing micronutrient malnutrition | | <https://pubmed.ncbi.nlm.nih.gov/31684687/> | | excluded by title and abstract | | |
|  | | Paulo HA et al., 2022 | Role of dietary quality and diversity on overweight and obesity among women of reproductive age in Tanzania | | https://journals.plos.org/plosone/article?id=10.1371/journal.pone.0266344 | | excluded by title and abstract | | |
|  | | Pate SK et al., 1996 | Tissue vitamin C levels of guinea pig offspring are influenced by maternal vitamin C intake during pregnancy | | https://go.exlibris.link/T2bF0MkQ | | excluded by title and abstract | | |
|  | | Pasricha SR et al., 2017 | How can recent insights into iron metabolism and infection help us overcome the global burden of anemia? | | <https://www.embase.com/search/results?subaction=viewrecord&id=L617420772&from=export> | | excluded by title and abstract | | |
|  | | Parkkali S et al., 2013 | Comparison of routine prenatal iron prophylaxis and screening and treatment for anaemia: pregnancy results and preliminary birth results from a pragmatic randomised controlled trial (PROFEG) in Maputo, Mozambique | | <https://pubmed.ncbi.nlm.nih.gov/23396557/> | | excluded by title and abstract | | |
|  | | Palawaththa S et al., 2022 | Effect of maternal dietary niacin intake on congenital anomalies: a systematic review and meta-analysis | | <https://www.embase.com/search/results?subaction=viewrecord&id=L2014145042&from=export> | | excluded by title and abstract | | |
|  | | Padaro E et al., 2019 | Intrauterine Growth Retardation Complicated by Biermer's Disease: An Observation in Togo | | <https://pubmed.ncbi.nlm.nih.gov/31198611/> | | excluded by title and abstract | | |
|  | | Owusu-Agyei S et al., 2013 | Impact of vitamin A with zinc supplementation on malaria morbidity in Ghana | | <https://pubmed.ncbi.nlm.nih.gov/24330422/> | | excluded by title and abstract | | |
|  | | Owie E, Afolabi BB, 2018 | Vitamin D deficiency in pregnant women and newborns in Lagos, Nigeria | | <https://pubmed.ncbi.nlm.nih.gov/29433370/> | | excluded by title and abstract | | |
|  | | Oumer M et al., 2021 | Birth prevalence of neural tube defects and associated risk factors in Africa: a systematic review and meta-analysis | | <https://pubmed.ncbi.nlm.nih.gov/33882899/> | | excluded by title and abstract | | |
|  | | Ouma P et al., 2006 | A randomized controlled trial of folate supplementation when treating malaria in pregnancy with sulfadoxine-pyrimethamine | | <https://pubmed.ncbi.nlm.nih.gov/17053829/> | | excluded by title and abstract | | |
|  | | Ouedraogo CT et al., 2019 | The mixed effects of a package of multilevel interventions on the health and care of pregnant women in Zinder, Niger | | <https://pubmed.ncbi.nlm.nih.gov/31908852/> | | excluded by title and abstract | | |
|  | | Ouédraogo S et al., 2012 | Maternal anemia at first antenatal visit: prevalence and risk factors in a malaria-endemic area in Benin | | <https://pubmed.ncbi.nlm.nih.gov/22826498/> | | excluded by title and abstract | | |
|  | | Ouédraogo CT et al., 2021 | Out-of-pocket costs and time spent attending antenatal care services: a case study of pregnant women in selected rural communities in Zinder, Niger | | <https://pubmed.ncbi.nlm.nih.gov/33419448/> | | excluded by title and abstract | | |
|  | | Otoluwa A et al., 2013 | The effect of moringa oleifera leaf extracts supplementation to the pregnant women in preventing maternal DNA damage | | <https://www.embase.com/search/results?subaction=viewrecord&id=L71181400&from=export> | | excluded by title and abstract | | |
|  | | Osunkalu VO et al., 2020 | Methylene tetrahydrofolate reductase and methionine synthase gene polymorphisms as genetic determinants of pre-eclampsia | | <https://pubmed.ncbi.nlm.nih.gov/32120336/> | | excluded by title and abstract | | |
|  | | Osunkalu VO et al., 2019 | Methylenetetrahydrofolate Reductase Enzyme Level and Antioxidant Activity in Women with Gestational Hypertension and Pre-eclampsia in Lagos, Nigeria | | <https://pubmed.ncbi.nlm.nih.gov/313917> | | excluded by title and abstract | | |
|  | | Osunkalu VO et al., 2021 | Epigenetic Modification in Methylene Tetrahydrofolate Reductase (MTHFR) Gene of Women with Pre-eclampsia | | <https://pubmed.ncbi.nlm.nih.gov/33814799/> | | excluded by title and abstract | | |
|  | | Osungbade K et al., 2008 | Content of antenatal care services in secondary health care facilities in Nigeria: implication for quality of maternal health care | | <https://pubmed.ncbi.nlm.nih.gov/18621778/> | | excluded by title and abstract | | |
|  | | Osifo BO, 1970 | The effect of folic acid and iron in the prevention of nutritional anaemias in pregnancy in Nigeria | | <https://pubmed.ncbi.nlm.nih.gov/5470772/> | | excluded by title and abstract | | |
|  | | Osaikhuwuomwan JA et al., 2011 | Plasma vitamin C levels and risk of preterm prelabour rupture of membranes | | <https://pubmed.ncbi.nlm.nih.gov/5470772/> | | excluded by title and abstract | | |
|  | | Omotayo MO et al., 2017 | A Simplified Regimen Compared with WHO Guidelines Decreases Antenatal Calcium Supplement Intake for Prevention of Preeclampsia in a Cluster-Randomized Noninferiority Trial in Rural Kenya | | <https://pubmed.ncbi.nlm.nih.gov/28878035/> | | excluded by title and abstract | | |
|  | | Omotayo MO et al., 2015 | Feasibility of integrating calcium and iron-folate supplementation to prevent preeclampsia and anemia in pregnancy in primary healthcare facilities in Kenya | | <https://pubmed.ncbi.nlm.nih.gov/26751372/> | | excluded by title and abstract | | |
|  | | Omotayo MO et al., 2018 | Feasibility of integrating calcium and iron-folate supplementation to prevent preeclampsia and anemia in pregnancy in primary healthcare facilities in Kenya | | <https://pubmed.ncbi.nlm.nih.gov/29493897/> | | excluded by title and abstract | | |
|  | | Omar AI et al., 2022 | Maternal Risk Factors Associated with Preterm Births among Pregnant Women in Mogadishu, Somalia | | <https://www.embase.com/search/results?subaction=viewrecord&id=L2019777895&from=export> | | excluded by title and abstract | | |
|  | | Olsson P, 1982 | Big venture to improve children's health | |  | | excluded by title and abstract | | |
|  | | Olusanya BO, Slusher TM, 2015 | World Journal of Pediatrics | |  | | excluded by title and abstract | | |
|  | | Okronipa H et al., 2018 | Maternal supplementation with small-quantity lipid-based nutrient supplements during pregnancy and lactation does not reduce depressive symptoms at 6 months postpartum in Ghanaian women: a randomized controlled trial | |  | | excluded by title and abstract | | |
|  | | Okpara SE et al., 2023 | Seasonal trend in the occurrence of myelomeningocele in Nigeria: a hypothesis of climate-induced oxidative stress | |  | | excluded by title and abstract | | |
|  | | Okala SG et al., 2019 | Impact of nutritional supplementation during pregnancy on antibody responses to diphtheria-tetanus-pertussis vaccination in infants: A randomised trial in The Gambia | |  | | excluded by title and abstract | | |
|  | | Okafor LA et al., 1985 | Bone Marrow Status of Anaemic Pregnant Women on Supplemental Iron and Folic Acid in a Nigerian Community | | <https://www.scopus.com/inward/record.uri?eid=2-s2.0-0021927459&doi=10.1177%2f000331978503600804&partnerID=40&md5=f89282355e5eaad6c3cb843325fbcf08> | | excluded by title and abstract | | |
|  | | Olsson P, 1982 | Big venture to improve children's health | |  | | excluded by title and abstract | | |
|  | | Olusanya BO, Slusher TM, 2015 | Infants at risk of significant hyperbilirubinemia in poorly-resourced countries: evidence from a scoping review | | <https://www.scopus.com/inward/record.uri?eid=2-s2.0-84943641023&doi=10.1007%2fs12519-015-0037-z&partnerID=40&md5=9c9a7224481e6470252492d6f943beef> | | excluded by title and abstract | | |
|  | | Oghale OP, Chris-Ozoko Nee Ebite LE, 2013 | Asyndromic bilateral transverse facial cleft | | <https://pubmed.ncbi.nlm.nih.gov/673893963/> | | excluded by title and abstract | | |
|  | | Ocansey ME et al., 2019 | The effects of supplementing maternal and infant diets with lipid-based nutrient supplements on physical activity and sedentary behaviour at preschool age in Ghana | | <https://pubmed.ncbi.nlm.nih.gov/31524123/> | | excluded by title and abstract | | |
|  | | Ocansey ME et al., 2019 | Prenatal and postnatal lipid-based nutrient supplementation and cognitive, social-emotional, and motor function in preschool-aged children in Ghana: a follow-up of a randomized controlled trial | | <https://pubmed.ncbi.nlm.nih.gov/30721937/> | | excluded by title and abstract | | |
|  | | O'Brien ME et al., 2005 | Anemia is an independent predictor of mortality and immunologic progression of disease among women with HIV in Tanzania | | <https://www.embase.com/search/results?subaction=viewrecord&id=L41395722&from=export> | | excluded by title and abstract | | |
|  | | Obi IF et al., 2022 | Descriptive epidemiology of external structural birth defects in Enugu State, Nigeria | | https://pubmed.ncbi.nlm.nih.gov/37575624/ | | excluded by title and abstract | | |
|  | | Obai G et al., 2016 | Prevalence of anaemia and associated risk factors among pregnant women attending antenatal care in Gulu and Hoima Regional Hospitals in Uganda: A cross sectional study | | https://www.embase.com/search/results?subaction=viewrecord&id=L609914309&from=export | | excluded by title and abstract | | |
|  | | Oaks BM et al., 2017 | Effects of a lipid-based nutrient supplement during pregnancy and lactation on maternal plasma fatty acid status and lipid profile: Results of two randomized controlled trials | | <https://pubmed.ncbi.nlm.nih.gov/28237085/> | | excluded by title and abstract | | |
|  | | Oaks BM et al., 2016 | Late-Pregnancy Salivary Cortisol Concentrations of Ghanaian Women Participating in a Randomized Controlled Trial of Prenatal Lipid-Based Nutrient Supplements | | <https://pubmed.ncbi.nlm.nih.gov/26764321/> | | excluded by title and abstract | | |
|  | | Oaks BM et al., 2020 | Impact of a nutritional supplement during gestation and early childhood on child salivary cortisol, hair cortisol, and telomere length at 4-6 years of age: a follow-up of a randomized controlled trial | | <https://pubmed.ncbi.nlm.nih.gov/32063089/> | | excluded by title and abstract | | |
|  | | Nylander PP, Adekunle AO, 1990 | Antenatal care in developing countries | | <https://pubmed.ncbi.nlm.nih.gov/2205429/> | | excluded by title and abstract | | |
|  | | Nwaru BI et al., 2015 | Adherence in a pragmatic randomized controlled trial on prophylactic iron supplementation during pregnancy in Maputo, Mozambique | | <https://pubmed.ncbi.nlm.nih.gov/24999785/> | | excluded by title and abstract | | |
|  | | Nwaru BI et al., 2015 | A pragmatic randomised controlled trial on routine iron prophylaxis during pregnancy in Maputo, Mozambique (PROFEG): rationale, design, and success | | <https://pubmed.ncbi.nlm.nih.gov/23020829/> | | excluded by title and abstract | | |
|  | | Nwagha UI et al., 2012 | Parity related changes in obesity and some antioxidant vitamins in non-pregnant women of South-Eastern Nigeria | | <https://pubmed.ncbi.nlm.nih.gov/23238183/> | | excluded by title and abstract | | |
|  | | Nwagha UI et al., 2011 | Asymptomatic malaria parasitemia does not induce additional oxidative stress in pregnant women of South East Nigeria | | <https://pubmed.ncbi.nlm.nih.gov/21771460/> | | excluded by title and abstract | | |
|  | | Norkus EP, Rosso P, 1981 | Effects of maternal intake of ascorbic acid on the postnatal metabolism of this vitamin in the guinea pig | | <https://pubmed.ncbi.nlm.nih.gov/7218035/> | | excluded by title and abstract | | |
|  | | Norkus EP, Rosso P, 1975 | Changes in ascorbic acid metabolism of the offspring following high maternal intake of this vitamin in the pregnant guinea pig | | <https://pubmed.ncbi.nlm.nih.gov/1060409/> | | excluded by title and abstract | | |
|  | | Noor RA et al., 2017 | Large-scale wheat flour folic acid fortification program increases plasma folate levels among women of reproductive age in urban Tanzania | | <https://pubmed.ncbi.nlm.nih.gov/28797054/> | | excluded by title and abstract | | |
|  | | Noor RA et al., 2020 | Prenatal zinc and Vitamin A reduce the benefit of iron on maternal hematologic and micronutrient status at delivery in Tanzania | | <https://academic.oup.com/jn/article/150/2/240/5588644> | | excluded by title and abstract | | |
|  | | Nojilana B et al., 2007 | Estimating the burden of disease attributable to iron deficiency anaemia in South Africa in 2000 | | <https://pubmed.ncbi.nlm.nih.gov/17952232/> | | excluded by title and abstract | | |
|  | | Nkabane-Nkholongo E et al., 2023 | Change in Sexual and Reproductive Health Knowledge among Young Women Using the Conversational Agent "Nthabi" in Lesotho: A Clinical Trial | | <https://pubmed.ncbi.nlm.nih.gov/38234736/> | | excluded by title and abstract | | |
|  | | Njiru H et al., 2022 | Effectiveness of public health education on the uptake of iron and folic acid supplements among pregnant women: a stepped wedge cluster randomised trial | | <https://pubmed.ncbi.nlm.nih.gov/15228241/> | | excluded by title and abstract | | |
|  | | Njagi JK et al., 2003 | Prevention of anaemia in pregnancy using insecticide-treated bednets and sulfadoxine-pyrimethamine in a highly malarious area of Kenya: a randomized controlled trial | | <https://pubmed.ncbi.nlm.nih.gov/15228241/> | | excluded by title and abstract | | |
|  | | Nilsen RM et al., 2019 | Preconception Folic Acid Supplement Use in Immigrant Women (1999-2016) | | <https://pubmed.ncbi.nlm.nih.gov/31569600/> | | excluded by title and abstract | | |
|  | | Ngounda J et al., 2021 | Socio-economic status and dietary intake of micronutrients in pregnant women in the urban Free State, South Africa | | <https://www.embase.com/search/results?subaction=viewrecord&id=L636086709&from=export> | | excluded by title and abstract | | |
|  | | Newman PM et al., 2009 | Placental malaria among HIV-infected and uninfected women receiving anti-folates in a high transmission area of Uganda | | <https://pubmed.ncbi.nlm.nih.gov/19912657/> | | excluded by title and abstract | | |
|  | | Neumann CG, Harrison GG, 1994 | Onset and evolution of stunting in infants and children. Examples from the Human Nutrition Collaborative Research Support Program. Kenya and Egypt studies | | <https://pubmed.ncbi.nlm.nih.gov/8005095/> | | excluded by title and abstract | | |
|  | | Ndyomugyenyi R et al., 2000 | Chloroquine prophylaxis, iron/folic-acid supplementation or case management of malaria attacks in primigravidae in western Uganda: Effects on congenital malaria and infant haemoglobin concentrations | | <https://www.embase.com/search/results?subaction=viewrecord&id=L32102486&from=export> | | excluded by title and abstract | | |
|  | | Nash CM et al., 2007 | Effects of maternal administration of vitamins C and E on ethanol neurobehavioral teratogenicity in the guinea pig | | <https://pubmed.ncbi.nlm.nih.gov/17980996/> | | excluded by title and abstract | | |
|  | | Napon C et al., 2012 | Polyneuropathies of pregnancy and the postpartum period in Burkina Faso | |  | | excluded by title and abstract | | |
|  | | Napier C et al., 2019 | Nutritional status and dietary diversity of pregnant women in rural KwaZulu-Natal, South Africa | | <https://pubmed.ncbi.nlm.nih.gov/31934418/> | | excluded by title and abstract | | |
|  | | Nankumbi J et al., 2022 | Predictors of vitamin A rich food consumption among women living in households growing orange-fleshed sweetpotatoes in selected regions in Uganda | | <https://pubmed.ncbi.nlm.nih.gov/36699868/> | | excluded by title and abstract | | |
|  | | Nankabirwa V et al., 2011 | Malaria parasitaemia among infants and its association with breastfeeding peer counselling and vitamin A supplementation: a secondary analysis of a cluster randomized trial | | <https://pubmed.ncbi.nlm.nih.gov/21760916/> | | excluded by title and abstract | | |
|  | | Nandi BK et al., 1977 | Effects of high intake of vitamin C by the guinea pigs in pregnancy and lactation on the tissue levels of the vitamin in their offspring | | <https://pubmed.ncbi.nlm.nih.gov/881299/> | | excluded by title and abstract | | |
|  | | Nandi BK et al., 1977 | Iron and hemoglobin status of the offspring of guinea pigs whose mother received large doses of vitamin C during pregnancy and lactation | | <https://pubmed.ncbi.nlm.nih.gov/914460/> | | excluded by title and abstract | | |
|  | | Naidoo Y et al., 2019 | Polymorphisms within vitamin D binding protein gene within a Preeclamptic South African population | | <https://pubmed.ncbi.nlm.nih.gov/31559882/> | | excluded by title and abstract | | |
|  | | Nahlen BL et al., 1989 | Lack of efficacy of pyrimethamine prophylaxis in pregnant Nigerian women | | <https://pubmed.ncbi.nlm.nih.gov/2571759/> | | excluded by title and abstract | | |
|  | | Musa IR et al., 2018 | Thyroid function and 25 (OH) vitamin D level among Sudanese women in early pregnancy | | <https://www.embase.com/search/results?subaction=viewrecord&id=L621378806&from=export> | | excluded by title and abstract | | |
|  | | Munyogwa MJ et al., 2021 | Effect of nutritional education intervention to reduce anaemia during pregnancy in Dodoma City, Tanzania: protocol for a cluster randomized controlled trial | | <https://pubmed.ncbi.nlm.nih.gov/34222670/> | | excluded by title and abstract | | |
|  | | Mulugeta SS, 2022 | Geographical disparities and determinants of adherence to iron folate supplementation among pregnant women in Ethiopia: spatial and multilevel analysis of the Ethiopian Mini Demographic and Health Survey of 2019 | | <https://pubmed.ncbi.nlm.nih.gov/36691126/> | | excluded by title and abstract | | |
|  | | Mulu GB et al., 2021 | Factors Associated With Neural Tube Defects Among Newborns Delivered at Debre Berhan Specialized Hospital, North Eastern Ethiopia, 2021. Case-Control Study | | <https://pubmed.ncbi.nlm.nih.gov/35295317/> | | excluded by title and abstract | | |
|  | | Mulu A et al., 2011 | Vitamin A deficiency during pregnancy of HIV infected and non-infected women in tropical settings of Northwest Ethiopia | | <https://pubmed.ncbi.nlm.nih.gov/21762514/> | | excluded by title and abstract | | |
|  | | Mulokozi G et al., 2003 | Plasma Levels of Retinol, Carotenoids, and Tocopherols in Relation to Dietary Pattern among Pregnant Tanzanian Women | | <https://www.embase.com/search/results?subaction=viewrecord&id=L37337481&from=export> | | excluded by title and abstract | | |
|  | | Mulhern MS et al., 2012 | Vitamin D status is a predictor of telomere length during pregnancy | | <https://www.embase.com/search/results?subaction=viewrecord&id=L71002379&from=export> | | excluded by title and abstract | | |
|  | | Mueller I et al., 2008 | A review of the current state of malaria among pregnant women in Papua New Guinea | | <https://pubmed.ncbi.nlm.nih.gov/19999304/> | | excluded by title and abstract | | |
|  | | Mubyazi GM, Bloch P, 2014 | Psychosocial, behavioural and health system barriers to delivery and uptake of intermittent preventive treatment of malaria in pregnancy in Tanzania - viewpoints of service providers in Mkuranga and Mufindi districts | | <https://www.embase.com/search/results?subaction=viewrecord&id=L373963084&from=export> | | excluded by title and abstract | | |
|  | | Msyamboza K et al., 2010 | Trends in pregnancy outcomes in Malawian adolescents receiving antimalarial and hematinic supplements | | <https://pubmed.ncbi.nlm.nih.gov/20528200/> | | excluded by title and abstract | | |
|  | | Mshanga N et al., 2019 | Food-basket intervention to reduce micronutrient deficiencies among Maasai-pregnant women in Tanzania: a quasi-experimental study | | <https://pubmed.ncbi.nlm.nih.gov/31131491/> | | excluded by title and abstract | | |
|  | | Moya-Alvarez V et al., 2021 | Vitamin C levels in a Central-African mother-infant cohort: Does hypovitaminosis C increase the risk of enteric infections? | | https://pubmed.ncbi.nlm.nih.gov/34137176/ | | excluded by title and abstract | | |
|  | | Moumouni A-EK et al., 2021 | PREVENTION OF SPINA BIFIDA: KNOWLEDGE, ATTITUDES AND PRACTICES OF MIDWIVES AND STATE AUXILIARY MIDWIVES IN THE HEALTH DISTRICT OF KOZAH (TOGO) | | https://www.embase.com/search/results?subaction=viewrecord&id=L2016850643&from=export | | excluded by title and abstract | | |
|  | | Motadi SA et al., 2020 | Assessment of Nutritional Status and Dietary Intake of Pregnant Women in Rural Area of Vhembe District, Limpopo Province | | https://www.embase.com/search/results?subaction=viewrecord&id=L629973245&from=export | | excluded by title and abstract | | |
|  | | Moreno Oliveras L et al., 2020 | Infant hydrocephalus in sub-Saharan Africa: Impact of perioperative care in the Zanzibar archipelago | | https://www.embase.com/search/results?subaction=viewrecord&id=L2005189676&from=export | | excluded by title and abstract | | |
|  | | Moore SE et al., 2012 | A randomized trial to investigate the effects of pre-natal and infant nutritional supplementation on infant immune development in rural Gambia: the ENID trial: Early Nutrition and Immune Development | | https://pubmed.ncbi.nlm.nih.gov/23057665/ | | excluded by title and abstract | | |
|  | | Moore PS et al., 1993 | Role of nutritional status and weight loss in HIV seroconversion among Rwandan women | | https://pubmed.ncbi.nlm.nih.gov/8496790/ | | excluded by title and abstract | | |
|  | | Monebenimp F et al., 2012 | Competence of health care providers on care of newborns at birth in a level-1 health facility in Yaoundé, Cameroon | | https://pubmed.ncbi.nlm.nih.gov/22593781/ | | excluded by title and abstract | | |
|  | | Mohan H et al., 2023 | Folate deficiency increases the incidence of dolutegravir-associated foetal defects in a mouse pregnancy model | | https://pubmed.ncbi.nlm.nih.gov/37586112/ | | excluded by title and abstract | | |
|  | | Mohammed BS, Helegbe GK, 2020 | Routine haematinics and multivitamins: Adherence and its association with haemoglobin level among pregnant women in an urban lower-middle-income country, Ghana | | https://pubmed.ncbi.nlm.nih.gov/31977143/ | | excluded by title and abstract | | |
|  | | Moya-Alvarez V et al., 2021 | Vitamin C levels in a Central-African mother-infant cohort: Does hypovitaminosis C increase the risk of enteric infections? | | https://pubmed.ncbi.nlm.nih.gov/34137176/ | | excluded by title and abstract | | |
|  | | Moges T et al., 2017 | Maternal vitamin D deficiency: A Culprit for Hypocalcaemia Induced Myocardial Failure in a Four-Month Old Infant: A Case Report From Tikur Anbessa Specialized Hospital, Ethiopia | | <https://pubmed.ncbi.nlm.nih.gov/29217929/> | | excluded by title and abstract | | |
|  | | Mkhize PZ et al., 2019 | Adherence to iron prophylactic therapy during pregnancy in an urban regional hospital in South Africa | | <https://www.embase.com/search/results?subaction=viewrecord&id=L2002767256&from=export> | | excluded by title and abstract | | |
|  | | Mitiku K et al., 2023 | Gross motor developmental delay and associated factors among under-five children attending public health facilities of Dessie city, Ethiopia | | <https://pubmed.ncbi.nlm.nih.gov/38110857/> | | excluded by title and abstract | | |
|  | | Mireku MO et al., 2018 | Consequences of prenatal geophagy for maternal prenatal health, risk of childhood geophagy and child psychomotor development | | <https://pubmed.ncbi.nlm.nih.gov/26055847/> | | excluded by title and abstract | | |
|  | | Mireku MO et al., 2015 | Prenatal Hemoglobin Levels and Early Cognitive and Motor Functions of One-Year-Old Children | | <https://pubmed.ncbi.nlm.nih.gov/26055847/> | | excluded by title and abstract | | |
|  | | Mireku MO et al., 2016 | Prenatal Iron Deficiency, Neonatal Ferritin, and Infant Cognitive Function | | <https://pubmed.ncbi.nlm.nih.gov/27940685/> | | excluded by title and abstract | | |
|  | | Millogo O et al., 2019 | Geographical variation in the association of child, maternal and household health interventions with under-five mortality in Burkina Faso | | <https://www.scopus.com/inward/record.uri?eid=2-s2.0-85069267562&doi=10.1371%2fjournal.pone.0218163&partnerID=40&md5=aa8458317f3bc66c20f7ba43e89254fa> | | excluded by title and abstract | | |
|  | | Miaffo C et al., 2004 | Malaria and anemia prevention in pregnant women of rural Burkina Faso | | <https://www.scopus.com/inward/record.uri?eid=2-s2.0-12944295047&doi=10.1186%2f1471-2393-4-18&partnerID=40&md5=412d9e1f83c9573370e23effd029b2c5> | | excluded by title and abstract | | |
|  | | Mgamb E et al., 2017 | Folate deficiency and utilization of folic acid fortified flour among pregnant women attending antenatal clinic at Pumwani Maternity Hospital, Kenya, 2015 | | <https://pubmed.ncbi.nlm.nih.gov/30197735/> | | excluded by title and abstract | | |
|  | | Mezzano J et al., 2022 | Effects of Iron and Vitamin A Levels on Pregnant Women and Birth Outcomes: Complex Relationships Untangled Using a Birth Cohort Study in Uganda | | <https://www.embase.com/search/results?subaction=viewrecord&id=L637411918&from=export> | | excluded by title and abstract | | |
|  | | Metz J et al., 1960 | The absorption of vitamin B12 in megaloblastic anaemia associated with pregnancy | | <https://www.embase.com/search/results?subaction=viewrecord&id=L281058516&from=export> | | excluded by title and abstract | | |
|  | | Metin Gulmezoglu A et al., 1997 | Antioxidants in the treatment of severe pre-eclampsia: An explanatory randomised controlled trial | | <https://www.embase.com/search/results?subaction=viewrecord&id=L27278352&from=export> | | excluded by title and abstract | | |
|  | | Menalu MM et al., 2021 | Assessment of Prevalence and Factors Associated with Malnutrition Among Under-Five Children in Debre Berhan Town, Ethiopia | | <https://pubmed.ncbi.nlm.nih.gov/33976568/> | | excluded by title and abstract | | |
|  | | Mekonen HK et al., 2021 | A silent epidemic of major congenital malformations in Tigray, northern Ethiopia: hospital-based study | | <https://pubmed.ncbi.nlm.nih.gov/34702882/> | | excluded by title and abstract | | |
|  | | Mehta U et al., 2018 | Assessing the value of western cape provincial government health administrative data and electronic pharmacy records in ascertaining medicine use during pregnancy | | <https://www.embase.com/search/results?subaction=viewrecord&id=L621927410&from=export> | | excluded by title and abstract | | |
|  | | Mehta S et al., 2010 | Lipid-soluble vitamins A, D, and E in HIV-infected pregnant women in Tanzania | | <https://www.embase.com/search/results?subaction=viewrecord&id=L50935713&from=export> | | excluded by title and abstract | | |
|  | | Mehta S et al., 2011 | Vitamin D status and its association with morbidity including wasting and opportunistic illnesses in HIV-infected women in Tanzania | | <https://pubmed.ncbi.nlm.nih.gov/21916603/> | | excluded by title and abstract | | |
|  | | Mehta S et al., 2009 | Perinatal outcomes, including mother-to-child transmission of HIV, and child mortality and their association with maternal vitamin D status in Tanzania | | <https://pubmed.ncbi.nlm.nih.gov/19673647/> | | excluded by title and abstract | | |
|  | | Mehta S et al., 2010 | Vitamin D status of HIV-infected women and its association with HIV disease progression, anemia, and mortality | | <https://pubmed.ncbi.nlm.nih.gov/20098738/> | | excluded by title and abstract | | |
|  | | Mchenga M et al., 2023 | Can women's reports in client exit interviews be used to measure and track progress of antenatal care services quality? Evidence from a facility assessment census in Malawi | | <https://pubmed.ncbi.nlm.nih.gov/37523376/> | | excluded by title and abstract | | |
|  | | McDonald C et al., 2012 | Morbidity and undernutrition are associated with impaired neurodevelopment among HIV-exposed infants in Tanzania | | https://www.embase.com/search/results?subaction=viewrecord&id=L70854316&from=export | | excluded by title and abstract | | |
|  | | Mbaye M et al., 2009 | Pregnancies associated with valvular prosthesis at Dakar Teaching Hospital: Prognosis, epidemiological, clinical and therapeutical aspects | | <https://www.embase.com/search/results?subaction=viewrecord&id=L50315137&from=export> | | excluded by title and abstract | | |
|  | | May PA et al., 2014 | Dietary intake, nutrition, and fetal alcohol spectrum disorders in the Western Cape Province of South Africa | | <https://www.embase.com/search/results?subaction=viewrecord&id=L372661391&from=export> | | excluded by title and abstract | | |
|  | | May PA et al., 2016 | Maternal nutritional status as a contributing factor for the risk of fetal alcohol spectrum disorders | | <https://pubmed.ncbi.nlm.nih.gov/26656914/> | | excluded by title and abstract | | |
|  | | Mave V et al., 2012 | Vitamin D deficiency is common among HIV-infected breastfeeding mothers in Pune, India, but is not associated with mother-to-child HIV transmission | | <https://pubmed.ncbi.nlm.nih.gov/23134628/> | | excluded by title and abstract | | |
|  | | Mason JB et al., 2014 | The first 500 days of life: policies to support maternal nutrition | | <https://pubmed.ncbi.nlm.nih.gov/24909407/> | | excluded by title and abstract | | |
|  | | Mason JB et al., 2012 | Opportunities for improving maternal nutrition and birth outcomes: synthesis of country experiences | | <https://pubmed.ncbi.nlm.nih.gov/22913110/> | | excluded by title and abstract | | |
|  | | Mason E et al., 2014 | Preconception care: advancing from ‘important to do and can be done’ to ‘is being done and is making a difference’ | | <https://pubmed.ncbi.nlm.nih.gov/22913110/> | | excluded by title and abstract | | |
|  | | Mashuda F et al., 2014 | Pattern and factors associated with congenital anomalies among young infants admitted at Bugando medical centre, Mwanza, Tanzania | | <https://www.embase.com/search/results?subaction=viewrecord&id=L604689839&from=export> | | excluded by title and abstract | | |
|  | | Martin SL et al., 2018 | Integrating Calcium Supplementation into Facility-Based Antenatal Care Services in Western Kenya: A Qualitative Process Evaluation to Identify Implementation Barriers and Facilitators | | <https://pubmed.ncbi.nlm.nih.gov/30402593/> | | excluded by title and abstract | | |
|  | | Martin SL et al., 2017 | Translating formative research findings into a behaviour change strategy to promote antenatal calcium and iron and folic acid supplementation in western Kenya | | <https://pubmed.ncbi.nlm.nih.gov/26898417/> | | excluded by title and abstract | | |
|  | | Martin SL et al., 2017 | Adherence-Specific Social Support Enhances Adherence to Calcium Supplementation Regimens among Pregnant Women | | <https://pubmed.ncbi.nlm.nih.gov/28250195/> | | excluded by title and abstract | | |
|  | | Martin SL et al., 2017 | Adherence partners are an acceptable behaviour change strategy to support calcium and iron-folic acid supplementation among pregnant women in Ethiopia and Kenya | | <https://pubmed.ncbi.nlm.nih.gov/27507135/> | | excluded by title and abstract | | |
|  | | Martinez L et al., 2022 | Vitamin D Concentrations in Infancy and the Risk of Tuberculosis Disease in Childhood: A Prospective Birth Cohort in Cape Town, South Africa | | <https://pubmed.ncbi.nlm.nih.gov/34436538/> | | excluded by title and abstract | | |
|  | | Mamme NY et al., 2023 | Serum folate deficiency and associated factors among pregnant women in Haramaya District, Eastern Ethiopia: a community-based study | | <https://pubmed.ncbi.nlm.nih.gov/37156586/> | | excluded by title and abstract | | |
|  | | Makola D et al., 2003 | A micronutrient-fortified beverage prevents iron deficiency, reduces anemia and improves the hemoglobin concentration of pregnant Tanzanian women | | <https://pubmed.ncbi.nlm.nih.gov/12730420/> | | excluded by title and abstract | | |
|  | | Makaula P et al., 2019 | Strengthening primary health care at district-level in Malawi - determining the coverage, costs and benefits of community-directed interventions | | <https://pubmed.ncbi.nlm.nih.gov/31331346/> | | excluded by title and abstract | | |
|  | | Mahmoud SZ et al., 2019 | Serum level of 25-hydroxyvitamin D and obesity among early pregnant women | | <https://pubmed.ncbi.nlm.nih.gov/31487758/> | | excluded by title and abstract | | |
|  | | Lweno ON et al., 2020 | Vitamin b12 is low in milk of early postpartum women in urban Tanzania, and was not significantly increased by high dose supplementation | | <https://www.embase.com/search/results?subaction=viewrecord&id=L2004143281&from=export> | | excluded by title and abstract | | |
|  | | Luwangula AK et al., 2022 | Improving Iron and Folic Acid Supplementation Among Pregnant Women: An Implementation Science Approach in East-Central Uganda | | <https://pubmed.ncbi.nlm.nih.gov/36951283/> | | excluded by title and abstract | | |
|  | | Luo C, 2000 | Strategies for prevention of mother-to-child transmission of HIV | | <https://pubmed.ncbi.nlm.nih.gov/11424244/> | | excluded by title and abstract | | |
|  | | Lukmanji Z et al., 2013 | Dietary patterns, nutrient intake, and sociodemographic characteristics in HIV-infected Tanzanian pregnant women | | <https://pubmed.ncbi.nlm.nih.gov/23282190/> | | excluded by title and abstract | | |
|  | | Liu E et al., 2022 | Multivitamin Supplementation Is Associated with Greater Adequacy of Gestational Weight Gain among Pregnant Women in Tanzania | | <https://pubmed.ncbi.nlm.nih.gov/34964890/> | | excluded by title and abstract | | |
|  | | Lindsay KL et al., 2012 | Maternal nutrition among women from Sub-Saharan Africa, with a focus on Nigeria, and potential implications for pregnancy outcomes among immigrant populations in developed countries | | <https://pubmed.ncbi.nlm.nih.gov/22594552/> | | excluded by title and abstract | | |
|  | | Lilungulu A et al., 2015 | Spectrum of maternal and perinatal outcomes among parturient women with preceding short inter-pregnancy interval at Bugando Medical Centre, Tanzania | | <https://pubmed.ncbi.nlm.nih.gov/27057319/> | | excluded by title and abstract | | |
|  | | Liljestrand J et al., 1986 | Anaemia of pregnancy in Mozambique | | <https://pubmed.ncbi.nlm.nih.gov/3787685/> | | excluded by title and abstract | | |
|  | | Lietz G et al., 2001 | Comparison of the effects of supplemental red palm oil and sunflower oil on maternal vitamin A status | | <https://www.embase.com/search/results?subaction=viewrecord&id=L32880261&from=export> | | excluded by title and abstract | | |
|  | | le Roux K et al., 2015 | The Role of Community Health Workers in the Re-Engineering of Primary Health Care in Rural Eastern Cape | | <https://pubmed.ncbi.nlm.nih.gov/26279948/> | | excluded by title and abstract | | |
|  | | LEONARD PJ, 1964 | VITAMIN E DEFICIENCY IN UGANDA AFRICAN SUBJECTS | | <https://pubmed.ncbi.nlm.nih.gov/14217010/> | | excluded by title and abstract | | |
|  | | Leidinger A et al., 2018 | Treating Pediatric Hydrocephalus at the Neurosurgery Education and Development Institute: The Reality in the Zanzibar Archipelago, Tanzania | | <https://www.embase.com/search/results?subaction=viewrecord&id=L2000933846&from=export> | | excluded by title and abstract | | |
|  | | Le Francois P et al., 1980 | Vitamin A status of populations in three West African countries | | <https://www.scopus.com/inward/record.uri?eid=2-s2.0-0019288652&partnerID=40&md5=60568665035aca29bce7da9ebbca68eb> | | excluded by title and abstract | | |
|  | | Lee AC et al., 2022 | Enhancing Nutrition and Antenatal Infection Treatment (ENAT) study: protocol of a pragmatic clinical effectiveness study to improve birth outcomes in Ethiopia | | <https://pubmed.ncbi.nlm.nih.gov/36053580/> | | excluded by title and abstract | | |
|  | | Lawrence M et al., 1987 | Energy requirements of pregnancy in The Gambia | | <https://pubmed.ncbi.nlm.nih.gov/2889977/> | | excluded by title and abstract | | |
|  | | Lauer JM et al., 2023 | Assessing environmental enteric dysfunction via multiplex assay and its relation to growth and development among HIV-exposed uninfected Tanzanian infants | | <https://www.embase.com/search/results?subaction=viewrecord&id=L2023301412&from=export> | | excluded by title and abstract | | |
|  | | Lauer JM et al., 2023 | Effects of Maternal Vitamin D Supplementation on Maternal and Infant Biomarkers of Environmental Enteric Dysfunction: A Secondary Analysis of a Randomized Controlled Trial | | <https://www.embase.com/search/results?subaction=viewrecord&id=L2026041771&from=export> | | excluded by title and abstract | | |
|  | | Latham MC et al., 2003 | Efficacy trials of a micronutrient dietary supplement in schoolchildren and pregnant women in Tanzania | | <https://www.embase.com/search/results?subaction=viewrecord&id=L38187935&from=export> | | excluded by title and abstract | | |
|  | | Lassi ZS et al., 2020 | Effects of preconception care and periconception interventions on maternal nutritional status and birth outcomes in low-and middle-income countries: a systematic review | | <https://pubmed.ncbi.nlm.nih.gov/674776564/> | | excluded by title and abstract | | |
|  | | Lankoandé M et al., 2020 | Snakebite Envenomation during a Third Trimester of Pregnancy: A Case Report | | <https://www.scopus.com/inward/record.uri?eid=2-s2.0-85122154296&doi=10.1097%2fFM9.0000000000000053&partnerID=40&md5=1fff201d16b2d9eff05abb8481093bfa> | | excluded by title and abstract | | |
|  | | Lammers CR et al., 2017 | Understanding women’s awareness and access to preconception health care in a rural population: a cross sectional study | |  | | excluded by title and abstract | | |
|  | | Laird E, et al 2012 | Vitamin D status in pregnancy: Data from the seychelles child development nutrition study | | https://www.embase.com/search/results?subaction=viewrecord&id=L71002312&from=export U2 - L71002312 | | excluded by title and abstract | | |
|  | | Laird E et al., 2017 | Maternal vitamin D status and the relationship with neonatal anthropometric and childhood neurodevelopmental outcomes: Results from the Seychelles child development nutrition study | | <https://www.embase.com/search/results?subaction=viewrecord&id=L619253268&from=export> | | excluded by title and abstract | | |
|  | | Lagerkvist CJ et al., 2020 | Predictors of Intention to Integrate Biofortified Orange-fleshed Sweetpotato in Child Feeding: A Field Information Experiment in Rural Kenya | | <https://pubmed.ncbi.nlm.nih.gov/32406767/> | | excluded by title and abstract | | |
|  | | Kusin JA et al., 1985 | Vitamin A status of pregnant and lactating women as assessed by serum levels in Machakos area, Kenya | | <https://pubmed.ncbi.nlm.nih.gov/4076026/> | | excluded by title and abstract | | |
|  | | Kupka R et al., 2008 | Randomized, double-blind, placebo-controlled trial of selenium supplements among HIV-infected pregnant women in Tanzania: Effects on maternal and child outcomes | | <https://www.embase.com/search/results?subaction=viewrecord&id=L351872645&from=export> | | excluded by title and abstract | | |
|  | | Kung'u JK et al., 2018 | Integrating nutrition into health systems at community level: Impact evaluation of the community-based maternal and neonatal health and nutrition projects in Ethiopia, Kenya, and Senegal | | <https://www.embase.com/search/results?subaction=viewrecord&id=L620982207&from=export> | | excluded by title and abstract | | |
|  | | Kung'u JK et al., 2018 | Design and implementation of a health systems strengthening approach to improve health and nutrition of pregnant women and newborns in Ethiopia, Kenya, Niger, and Senegal | | <https://pubmed.ncbi.nlm.nih.gov/29493901/> | | excluded by title and abstract | | |
|  | | Kumwenda N et al., 2002 | Antenatal vitamin A supplementation increases birth weight and decreases anemia among infants born to human immunodeficiency virus-infected women in Malawi | | <https://pubmed.ncbi.nlm.nih.gov/12173139/> | | excluded by title and abstract | | |
|  | | Kumordzie SM et al., 2019 | Maternal-Infant Supplementation with Small-Quantity Lipid-Based Nutrient Supplements Does Not Affect Child Blood Pressure at 4-6 Y in Ghana: Follow-up of a Randomized Trial | | <https://pubmed.ncbi.nlm.nih.gov/30753625/> | | excluded by title and abstract | | |
|  | | Kumordzie SM et al., 2019 | Maternal and Infant Lipid-Based Nutritional Supplementation Increases Height of Ghanaian Children at 4-6 Years Only if the Mother Was Not Overweight Before Conception | | <https://pubmed.ncbi.nlm.nih.gov/31034033/> | | excluded by title and abstract | | |
|  | | Kulkarni MA et al., 2010 | Contribution of integrated campaign distribution of long-lasting insecticidal nets to coverage of target groups and total populations in malaria-endemic areas in Madagascar | | <https://pubmed.ncbi.nlm.nih.gov/20207867/> | | excluded by title and abstract | | |
|  | | Kucha W et al., 2022 | Folate, Vitamin B12, and Homocysteine Levels in Women With Neural Tube Defect-Affected Pregnancy in Addis Ababa, Ethiopia | | <https://pubmed.ncbi.nlm.nih.gov/35464038/> | | excluded by title and abstract | | |
|  | | Kæstel P et al., 2012 | Serum retinol is associated with stage of pregnancy and the acute phase response in pregnant women in Guinea-Bissau | | <https://pubmed.ncbi.nlm.nih.gov/22437561/> | | excluded by title and abstract | | |
|  | | Krige SM et al., 2018 | Dietary intake and beliefs of pregnant women with gestational diabetes in Cape Town, South Africa | | <https://www.embase.com/search/results?subaction=viewrecord&id=L623642126&from=export> | | excluded by title and abstract | | |
|  | | Kpoda DS et al., 2022 | Nutritional, Microbiological, and Toxicological Quality Assessment of Foods Sold in Urban and Suburban Markets in Burkina Faso | | <https://pubmed.ncbi.nlm.nih.gov/35917509/> | | excluded by title and abstract | | |
|  | | Kourtis AP et al., 2023 | Dolutegravir and pregnancy outcomes including neural tube defects in the USA during 2008-20: a national cohort study | | <https://pubmed.ncbi.nlm.nih.gov/37506721/> | | excluded by title and abstract | | |
|  | | Koricho Z et al., 2020 | Dietary vitamin A intakes among pregnant women attending antenatal care in health facilities in Dessie Town, North East Ethiopia | | <https://pubmed.ncbi.nlm.nih.gov/32424967/> | | excluded by title and abstract | | |
|  | | Koné S et al., 2020 | Study protocol of a cluster randomized controlled trial of strategies to increase antenatal iron and folic acid supplementation and malaria prophylaxis in rural south-central Côte d'Ivoire | | <https://pubmed.ncbi.nlm.nih.gov/33109138/> | | excluded by title and abstract | | |
|  | | Koné S et al., 2023 | Improving coverage of antenatal iron and folic acid supplementation and malaria prophylaxis through targeted information and home deliveries in Côte d'Ivoire: a cluster randomised controlled trial | | <https://pubmed.ncbi.nlm.nih.gov/33109138/> | | excluded by title and abstract | | |
|  | | Koné S et al., 2018 | Pregnancy-related morbidity and risk factors for fatal foetal outcomes in the Taabo health and demographic surveillance system, Côte d'Ivoire | | <https://pubmed.ncbi.nlm.nih.gov/29879939/> | | excluded by title and abstract | | |
|  | | Komolafe EO et al., 2018 | The pattern, peculiarities, and management challenges of spina bifida in a teaching hospital in Southwest Nigeria | | <https://pubmed.ncbi.nlm.nih.gov/29026989/> | | excluded by title and abstract | | |
|  | | Klevor MK et al., 2016 | A mixed method study exploring adherence to and acceptability of small quantity lipid-based nutrient supplements (SQ-LNS) among pregnant and lactating women in Ghana and Malawi | | <https://pubmed.ncbi.nlm.nih.gov/27577112/> | | excluded by title and abstract | | |
|  | | Kisinza WN et al., 2008 | Community directed interventions for malaria, tuberculosis and vitamin A in onchocerciasis endemic districts of Tanzania | | <https://pubmed.ncbi.nlm.nih.gov/19402585/> | | excluded by title and abstract | | |
|  | | Kishimba RS et al., 2015 | Factors associated with major structural birth defects among newborns delivered at Muhimbili National Hospital and Municipal Hospitals in Dar Es Salaam, Tanzania 2011 - 2012 | | <https://pubmed.ncbi.nlm.nih.gov/26525082/> | | excluded by title and abstract | | |
|  | | Kirkwood BR et al., 2010 | Effect of vitamin A supplementation in women of reproductive age on maternal survival in Ghana (ObaapaVitA): a cluster-randomised, placebo-controlled trial | | <https://pubmed.ncbi.nlm.nih.gov/20435345/> | | excluded by title and abstract | | |
|  | | Kirby M et al., 2022 | Biomarkers of Environmental Enteric Dysfunction in Pregnancy and Adverse Birth Outcomes: An Observational Study Among Women Living With HIV in Tanzania | | <https://www.embase.com/search/results?subaction=viewrecord&id=L2023724822&from=export> | | excluded by title and abstract | | |
|  | | Kiondo P et al., 2011 | Plasma vitamin C concentration in pregnant women with pre-eclampsia in Mulago hospital, Kampala, Uganda | | <https://pubmed.ncbi.nlm.nih.gov/22649436/> | | excluded by title and abstract | | |
|  | | Kiondo P et al., 2014 | The effects of vitamin C supplementation on pre-eclampsia in Mulago Hospital, Kampala, Uganda: A randomized placebo controlled clinical trial | | <https://www.embase.com/search/results?subaction=viewrecord&id=L600014114&from=export> | | excluded by title and abstract | | |
|  | | Kim SS et al., 2023 | Intensified Nutrition Interventions in Antenatal Care Services Increased Consumption of Iron and Folic Acid Supplements and Early Breastfeeding Practices in Burkina Faso: Results of a Cluster-Randomized Program Evaluation | | <https://pubmed.ncbi.nlm.nih.gov/37336320/> | | excluded by title and abstract | | |
|  | | Kidane R et al., 2022 | Dietary Patterns and Hypertensive Disorders Among Pregnant Women Attending Antenatal and Delivery Care in Public Hospitals of Jimma Zone, Southwest Ethiopia | | <https://pubmed.ncbi.nlm.nih.gov/35937311/> | | excluded by title and abstract | | |
|  | | Khavari N et al., 2014 | Maternal multivitamin supplementation reduces the risk of diarrhoea among HIV-exposed children through age 5 years | | <https://www.embase.com/search/results?subaction=viewrecord&id=L603241676&from=export> | | excluded by title and abstract | | |
|  | | Khanal LD, 2020 | Knowledge and utilization of preconception care among women in selected community of Kathmandu | | <https://pubmed.ncbi.nlm.nih.gov/676013154/> | | excluded by title and abstract | | |
|  | | Keverenge-Ettyang GA et al., 2006 | Maternal nutritional status in pastoral versus farming communities of West Pokot, Kenya: differences in iron and vitamin A status and body composition | | <https://pubmed.ncbi.nlm.nih.gov/17542113/> | | excluded by title and abstract | | |
|  | | Kesa H et al., 2010 | The nutritional status of pregnant women in the Vaal region, South Africa: Is there a need for intervention? | | <https://www.embase.com/search/results?subaction=viewrecord&id=L71007558&from=export> | | excluded by title and abstract | | |
|  | | Kenyon M et al., 1983 | Weight changes during pregnancy and birthweights on Rusinga Island | | <https://pubmed.ncbi.nlm.nih.gov/12267055/> | | excluded by title and abstract | | |
|  | | Kennedy C et al., 2001 | Randomized controlled trial assessing the effect of vitamin A supplementation on maternal morbidity during pregnancy and postpartum among HIV-infected women | | <https://www.embase.com/search/results?subaction=viewrecord&id=L30447804&from=export> | | excluded by title and abstract | | |
|  | | Kennedy C et al., 2001 | Factors Associated with Singleton Preterm Birth in Shire Suhul General Hospital, Northern Ethiopia, 2018 | | <https://pubmed.ncbi.nlm.nih.gov/31205788/> | | excluded by title and abstract | | |
|  | | Kisinza WN et al., 2008 | Community directed interventions for malaria, tuberculosis and vitamin A in onchocerciasis endemic districts of Tanzania | | <https://pubmed.ncbi.nlm.nih.gov/19402585/> | | excluded by title and abstract | | |
|  | | Kishimba RS et al., 2015 | Factors associated with major structural birth defects among newborns delivered at Muhimbili National Hospital and Municipal Hospitals in Dar Es Salaam, Tanzania 2011 - 2012 | | <https://pubmed.ncbi.nlm.nih.gov/26525082/> | | excluded by title and abstract | | |
|  | | Kirkwood BR et al., 2010 | Effect of vitamin A supplementation in women of reproductive age on maternal survival in Ghana (ObaapaVitA): a cluster-randomised, placebo-controlled trial | | <https://pubmed.ncbi.nlm.nih.gov/20435345/> | | excluded by title and abstract | | |
|  | | Kirby M et al., 2022 | Biomarkers of Environmental Enteric Dysfunction in Pregnancy and Adverse Birth Outcomes: An Observational Study Among Women Living With HIV in Tanzania | | <https://www.embase.com/search/results?subaction=viewrecord&id=L2023724822&from=export> | | excluded by title and abstract | | |
|  | | Kawai K et al., 2010 | Sex differences in the effects of maternal vitamin supplements on mortality and morbidity among children born to HIV-infected women in Tanzania | | <https://www.embase.com/search/results?subaction=viewrecord&id=L50822827&from=export> | | excluded by title and abstract | | |
|  | | Kawai K et al., 2010 | A randomized trial to determine the optimal dosage of multivitamin supplements to reduce adverse pregnancy outcomes among HIV-infected women in Tanzania | | <https://www.embase.com/search/results?subaction=viewrecord&id=L358210666&from=export> | | excluded by title and abstract | | |
|  | | Kassahun E et al., 2023 | Level and Predictors of Minimum Dietary Diversity Among Pregnant Women in Eastern Ethiopia: Evidence From Facility-Based Cross-sectional Survey | | <https://pubmed.ncbi.nlm.nih.gov/37114982/> | | excluded by title and abstract | | |
|  | | Kassa A et al., 2018 | Knowledge of preconception care among healthcare providers working in public health institutions in Hawassa, Ethiopia | | <https://pubmed.ncbi.nlm.nih.gov/676013154/> | | excluded by title and abstract | | |
|  | | KARLIN R, 1962 | [On the distribution of vitamin B-6 during the intrauterine life of the guinea pig in the maternal and fetal organs] | | <https://pubmed.ncbi.nlm.nih.gov/14453981/> | | excluded by title and abstract | | |
|  | | KARLIN R, 1957 | [Effects of oral or parenteral administration of vitamin B12 on distribution and storage during the intra-uterine life of guinea pig] | | <https://pubmed.ncbi.nlm.nih.gov/13472325/> | | excluded by title and abstract | | |
|  | | Karasov WH et al., 1991 | Dietary regulation of intestinal ascorbate uptake in guinea pigs | | <https://pubmed.ncbi.nlm.nih.gov/1987799/> | | excluded by title and abstract | | |
|  | | Kanu FA et al., 2023 | Association between Hemoglobin and Elevation among School-aged Children: A Verification of Proposed Adjustments | | <https://pubmed.ncbi.nlm.nih.gov/37059418/> | | excluded by title and abstract | | |
|  | | Kancherla V et al., 2021 | Projected impact of mandatory food fortification with folic acid on neurosurgical capacity needed for treating spina bifida in Ethiopia | | <https://pubmed.ncbi.nlm.nih.gov/33319513/> | | excluded by title and abstract | | |
|  | | Kancherla V et al., 2021 | Reducing the burden of anemia and neural tube defects in low-and middle-income countries: An analysis to identify countries with an immediate potential to benefit from large-scale mandatory fortification of wheat flour and rice | | <https://www.scopus.com/inward/record.uri?eid=2-s2.0-85100177545&doi=10.3390%2fnu13010244&partnerID=40&md5=770bb3b4a1fb6d6365ac97db12802d76> | | excluded by title and abstract | | |
|  | | Kamng'ona AW et al., 2020 | Provision of Lipid-Based Nutrient Supplements to Mothers During Pregnancy and 6 Months Postpartum and to Their Infants from 6 to 18 Months Promotes Infant Gut Microbiota Diversity at 18 Months of Age but Not Microbiota Maturation in a Rural Malawian Setting: Secondary Outcomes of a Randomized Trial | | <https://pubmed.ncbi.nlm.nih.gov/31909811/> | | excluded by title and abstract | | |
|  | | Kamau MW et al., 2020 | Effect of a community-based approach of iron and folic acid supplementation on compliance by pregnant women in Kiambu County, Kenya: A quasi-experimental study | | <https://pubmed.ncbi.nlm.nih.gov/31923240/> | | excluded by title and abstract | | |
|  | | Kamau MW, 2020 | Time for change is now: Experiences of participants in a community-based approach for iron and folic acid supplementation in a rural county in Kenya, a qualitative study | | <https://pubmed.ncbi.nlm.nih.gov/31945073/> | | excluded by title and abstract | | |
|  | | Kamau MW et al., 2020 | Effect of community-based health education on knowledge and attitude towards iron and folic acid supplementation among pregnant women in Kiambu County, Kenya: A quasi-experimental study | | <https://pubmed.ncbi.nlm.nih.gov/31765422/> | | excluded by title and abstract | | |
|  | | Kamau M et al., 2019 | Diet quality of pregnant women attending an antenatal clinic in Nakuru, Kenya | | <https://pubmed.ncbi.nlm.nih.gov/31765422/> | | excluded by title and abstract | | |
|  | | Kamau-Mbuthia E, Elmadfa I, 2007 | Positive impact on vitamin D related lifestyle of medical advice in pregnant Somali-born women and new mothers: a mixed method study in Swedish primary care | | <https://pubmed.ncbi.nlm.nih.gov/17726309/> | | excluded by title and abstract | | |
|  | | Kalliokoski P et al., 2021 | Long-term adherence and effects on grip strength and upper leg performance of prescribed supplemental vitamin D in pregnant and recently pregnant women of Somali and Swedish birth with 25-hydroxyvitamin D deficiency: a before-and-after treatment study | | <https://pubmed.ncbi.nlm.nih.gov/33546641/> | | excluded by title and abstract | | |
|  | | Kalliokoski P et al., 2016 | Physical performance and 25-hydroxyvitamin D: A cross-sectional study of pregnant Swedish and Somali immigrant women and new mothers | | <https://pubmed.ncbi.nlm.nih.gov/27846821/> | | excluded by title and abstract | | |
|  | | Kalinjuma AV et al., 2019 | Vitamin D Concentration during Early Pregnancy and Adverse Outcomes among HIV-Negative Women in Dar-es-Salaam, Tanzania: A Case-Control Study | | <https://www.embase.com/search/results?subaction=viewrecord&id=L52924085&from=export> | | excluded by title and abstract | | |
|  | | Justine C, 2022 | Prevalence, patterns and associated factors for neural tube defects among young infants admitted at Bugando Medical Centre in North-western Tanzania | | <https://www.embase.com/search/results?subaction=viewrecord&id=L639442003&from=export> | | excluded by title and abstract | | |
|  | | Jugha VT et al., 2020 | Dietary Diversity and Its Contribution in the Etiology of Maternal Anemia in Conflict Hit Mount Cameroon Area: A Cross-Sectional Study | | https://pubmed.ncbi.nlm.nih.gov/33614694/ | | excluded by title and abstract | | |
|  | | Jourabchi Z et al., 2019 | Public finance policy strategies to increase access to preconception care | | <https://www.embase.com/search/results?subaction=viewrecord&id=L52924085&from=export> | | excluded by title and abstract | | |
|  | | Johnson W et al., 2017 | Maternal micronutrient status as a risk factor for perinatal mortality in a Tanzanian randomized controlled trial | | <https://www.embase.com/search/results?subaction=viewrecord&id=L642045204&from=export> | | excluded by title and abstract | | |
|  | | Johnson J, 2022 | Vitamin C status, serum cholesterol levels and bile composition in the pregnant guinea-pig | | <https://pubmed.ncbi.nlm.nih.gov/7370221/> | | excluded by title and abstract | | |
|  | | Kamng'ona AW et al., 2020 | Provision of Lipid-Based Nutrient Supplements to Mothers During Pregnancy and 6 Months Postpartum and to Their Infants from 6 to 18 Months Promotes Infant Gut Microbiota Diversity at 18 Months of Age but Not Microbiota Maturation in a Rural Malawian Setting: Secondary Outcomes of a Randomized Trial | |  | | excluded by title and abstract | | |
|  | | Kamau MW et al., 2020 | Effect of a community-based approach of iron and folic acid supplementation on compliance by pregnant women in Kiambu County, Kenya: A quasi-experimental study | | <https://pubmed.ncbi.nlm.nih.gov/31923240/> | | excluded by title and abstract | | |
|  | | Kamau MW, 2020 | Time for change is now: Experiences of participants in a community-based approach for iron and folic acid supplementation in a rural county in Kenya, a qualitative study | | <https://pubmed.ncbi.nlm.nih.gov/31945073/> | | excluded by title and abstract | | |
|  | | Kamau MW et al., 2020 | Effect of community-based health education on knowledge and attitude towards iron and folic acid supplementation among pregnant women in Kiambu County, Kenya: A quasi-experimental study | | <https://pubmed.ncbi.nlm.nih.gov/31765422/> | | excluded by title and abstract | | |
|  | | Kamau M et al., 2019 | Diet quality of pregnant women attending an antenatal clinic in Nakuru, Kenya | | <https://pubmed.ncbi.nlm.nih.gov/31765422/> | | excluded by title and abstract | | |
|  | | Kamau-Mbuthia E, Elmadfa I, 2007 | Positive impact on vitamin D related lifestyle of medical advice in pregnant Somali-born women and new mothers: a mixed method study in Swedish primary care | | <https://pubmed.ncbi.nlm.nih.gov/17726309/> | | excluded by title and abstract | | |
|  | | Jemal S, Fentahun E, Oumer M, Muche A, 2021 | Predictors of congenital anomalies among newborns in Arsi zone public hospitals, Southeast Ethiopia: a case-control study | | <https://pubmed.ncbi.nlm.nih.gov/34193221/> | | excluded by title and abstract | | |
|  | | James PT et al., 2019 | A novel nutritional supplement to reduce plasma homocysteine in nonpregnant women: A randomised controlled trial in The Gambia | | <https://pubmed.ncbi.nlm.nih.gov/31408467/> | | excluded by title and abstract | | |
|  | | Jackson RT, Latham MC, 1982 | Anemia of pregnancy in Liberia, West Africa: a therapeutic trial | | <https://pubmed.ncbi.nlm.nih.gov/7072624/> | | excluded by title and abstract | | |
|  | | Isanaka S et al., 2021 | Immunogenicity of an oral rotavirus vaccine administered with prenatal nutritional support in Niger: A cluster randomized clinical trial | | <https://pubmed.ncbi.nlm.nih.gov/34375336/> | | excluded by title and abstract | | |
|  | | Ingram CF, Fleming AF, Patel M, Galpin JS, 1999 | Pregnancy- and lactation-related folate deficiency in South Africa - A case for folate food fortification | | <https://www.embase.com/search/results?subaction=viewrecord&id=L30058186&from=export> | | excluded by title and abstract | | |
|  | | Ikpen MA et al., 2012 | Determination of antioxidant status of pre-eclamptic and normotensive sub-rural Nigerian pregnant women at the Irrua Specialist Teaching Hospital, Irrua, Edo State | | <https://pubmed.ncbi.nlm.nih.gov/22551196/> | | excluded by title and abstract | | |
|  | | Ibrahim SNY, Mohammed KY, 2019 | Prevalence of vitamin D deficiency among pregnant Sudanese women | | <https://www.embase.com/search/results?subaction=viewrecord&id=L628473841&from=export> | | excluded by title and abstract | | |
|  | | Ibadin OM, Osubor CC, Onoberhie PA, 2009 | Alpha-tocopherol levels in milk of exclusively breast-feeding mothers in Benin City, Nigeria | | <https://www.scopus.com/inward/record.uri?eid=2-s2.0-77956401707&partnerID=40&md5=dccb73c2351261393e1554bc72029265> | | excluded by title and abstract | | |
|  | | Jemal S, Fentahun E, Oumer M, Muche A, 2021 | Predictors of congenital anomalies among newborns in Arsi zone public hospitals, Southeast Ethiopia: a case-control study | | <https://pubmed.ncbi.nlm.nih.gov/34193221/> | | excluded by title and abstract | | |
|  | | James PT et al., 2019 | A novel nutritional supplement to reduce plasma homocysteine in nonpregnant women: A randomised controlled trial in The Gambia | | <https://pubmed.ncbi.nlm.nih.gov/31408467/> | | excluded by title and abstract | | |
|  | | Hurley LS, Volkert NE, Eichner JT, 1965 | Pantothenic Acid Deficiency in Pregnant and Non-Pregnant Guinea Pigs, with Special Reference to Effects on the Fetus | | <https://pubmed.ncbi.nlm.nih.gov/14302122/> | | excluded by title and abstract | | |
|  | | Hunter-Adams J, Rother H-A, 2016 | Pregnant in a foreign city: A qualitative analysis of diet and nutrition for cross-border migrant women in Cape Town, South Africa | | <https://www.embase.com/search/results?subaction=viewrecord&id=L610387711&from=export> | | excluded by title and abstract | | |
|  | | Hull J.C., Bloch E.M., Ingram C., Crookes R., Mack N., Courtney L., Murphy E.L., 2017 | Understanding iron deficiency anemia among HIV positive and negative pregnant women in South Africa: A first step toward prevention of peripartum blood transfusion | | <https://www.embase.com/search/results?subaction=viewrecord&id=L618448218&from=export> | | excluded by title and abstract | | |
|  | | Huddle JM, Gibson RS, Cullinan TR, 1999 | The impact of malarial infection and diet on the anaemia status of rural pregnant Malawian women | | <https://pubmed.ncbi.nlm.nih.gov/28217860/> | | excluded by title and abstract | | |
|  | | Horjus P, Aguayo VM, Roley JA, Pene MC, Meershoek SP, 2005 | School-based iron and folic acid supplementation for adolescent girls: findings from Manica Province, Mozambique | | <https://pubmed.ncbi.nlm.nih.gov/16222919/> | | excluded by title and abstract | | |
|  | | Hong BV, Zheng JJ, Romo EZ, Agus JK, Tang X, Arnold CD, Adu-Afarwuah S, Lartey A, Okronipa H, Dewey KG, Zivkovic AM, 2023 | Seasonal Factors Are Associated with Activities of Enzymes Involved in High-Density Lipoprotein Metabolism among Pregnant Females in Ghana | | <https://pubmed.ncbi.nlm.nih.gov/38130330/> | | excluded by title and abstract | | |
|  | | Hjertholm KG, Iversen PO, Holmboe-Ottesen G, Mdala I, Munthali A, Maleta K, Shi Z, Ferguson E, Kamudoni P, 2018 | The Association of Feeding Practices and Sociodemographic Factors on Underweight and Wasting in Children in Ethiopia: A Secondary Analysis of Four Health Surveys from 2000 to 2016 | | <https://pubmed.ncbi.nlm.nih.gov/28217860/> | | excluded by title and abstract | | |
|  | | Hiruy AF, Xiong Q, Jin Q, Zhao J, Lin X, He S, Abebe A, Zuo X, Ying C, 2021 | Dietary 'fibre' and pregnancy toxaemia | | <https://pubmed.ncbi.nlm.nih.gov/34450644/> | | excluded by title and abstract | | |
|  | | Hipsley EH, 1953 | Maternal dietary intake during pregnancy and its association to birth size in rural Malawi: A cross-sectional study | | <https://pubmed.ncbi.nlm.nih.gov/28217860/> | | excluded by title and abstract | | |
|  | | Hinderaker SG et al., 2002 | Anemia in pregnancy in rural Tanzania: Associations with micronutrient status and infections | | <https://www.embase.com/search/results?subaction=viewrecord&id=L34223929&from=export> | | excluded by title and abstract | | |
|  | | Hidiroglou N et al., 2003 | Influence of sources of dietary vitamin E on the maternal transfer of alpha-tocopherol to fetal and neonatal guinea pigs as determined by a stable isotopic technique | | <https://pubmed.ncbi.nlm.nih.gov/12654163/> | | excluded by title and abstract | | |
|  | | Hewitt AJ et al., 2011 | Chronic ethanol exposure and folic acid supplementation: fetal growth and folate status in the maternal and fetal guinea pig | | <https://pubmed.ncbi.nlm.nih.gov/21315145/> | | excluded by title and abstract | | |
|  | | Hess SY et al., 2018 | Using formative research to promote antenatal care attendance and iron folic acid supplementation in Zinder, Niger | | <https://pubmed.ncbi.nlm.nih.gov/28924978/> | | excluded by title and abstract | | |
|  | | Herman WH, Janz NK, Becker MP, Charron-Prochownik D, 1999 | Diabetes and pregnancy: preconception care, pregnancy outcomes, resource utilization and costs | | <https://www.embase.com/search/results?subaction=viewrecord&id=L34223929&from=export> | | excluded by title and abstract | | |
|  | | Hercberg S et al., 1987 | Nutritional anaemia in pregnant Beninese women: consequences on the haematological profile of the newborn | | <https://pubmed.ncbi.nlm.nih.gov/3552028/> | | excluded by title and abstract | | |
|  | | Hemminki E et al., 2016 | Is selective prenatal iron prophylaxis better than routine prophylaxis: final results of a trial (PROFEG) in Maputo, Mozambique | | <https://pubmed.ncbi.nlm.nih.gov/27297013/> | | excluded by title and abstract | | |
|  | | Hasken JM et al., 2023 | Maternal dietary intake among alcohol-exposed pregnancies is linked to early infant physical outcomes in South Africa | | <https://pubmed.ncbi.nlm.nih.gov/37678653/> | | excluded by title and abstract | | |
|  | | Hasken J et al., 2013 | Nutrition and body mass index examined as contributing factors for the risk of fetal alcohol spectrum disorders | | <https://www.embase.com/search/results?subaction=viewrecord&id=L71068472&from=export> | | excluded by title and abstract | | |
|  | | Haskell MJ et al., 2021 | Small-Quantity Lipid-Based Nutrient Supplements Do Not Affect Plasma or Milk Retinol Concentrations Among Malawian Mothers, or Plasma Retinol Concentrations among Young Malawian or Ghanaian Children in Two Randomized Trials | | <https://pubmed.ncbi.nlm.nih.gov/33561214/> | | excluded by title and abstract | | |
|  | | Haskell MJ et al., 2022 | Provision of Small-Quantity Lipid-Based Nutrient Supplements Increases Plasma Selenium Concentration in Pregnant Women in Malawi: A Secondary Outcome of a Randomized Controlled Trial | | <https://pubmed.ncbi.nlm.nih.gov/35317414/> | | excluded by title and abstract | | |
|  | | Harrison GG, 1992 | Nutrition and child feeding patterns in the Sudan | | <https://pubmed.ncbi.nlm.nih.gov/12319275/> | | excluded by title and abstract | | |
|  | | Harjunmaa U et al., 2016 | Nutrient supplementation may adversely affect maternal oral health--a randomised controlled trial in rural Malawi | | <https://pubmed.ncbi.nlm.nih.gov/26194850/> | | excluded by title and abstract | | |
|  | | Harika R et al., 2017 | Micronutrient Status and Dietary Intake of Iron, Vitamin A, Iodine, Folate and Zinc in Women of Reproductive Age and Pregnant Women in Ethiopia, Kenya, Nigeria and South Africa: A Systematic Review of Data from 2005 to 2015 | | <https://pubmed.ncbi.nlm.nih.gov/28981457/> | | excluded by title and abstract | | |
|  | | Hanson C et al., 2018 | Status of Retinoids and Carotenoids and Associations with Clinical Outcomes in Maternal-Infant Pairs in Nigeria | | <https://pubmed.ncbi.nlm.nih.gov/30213044/> | | excluded by title and abstract | | |
|  | | Hansen SN et al., 2016 | Maternal vitamin C deficiency does not reduce hippocampal volume and β-tubulin III intensity in prenatal Guinea pigs | | <https://pubmed.ncbi.nlm.nih.gov/27333961/> | | excluded by title and abstract | | |
|  | | Hansen SN et al., 2018 | Early Life Vitamin C Deficiency Does Not Alter Morphology of Hippocampal CA1 Pyramidal Neurons or Markers of Synaptic Plasticity in a Guinea Pig Model | | <https://pubmed.ncbi.nlm.nih.gov/29890692/> | | excluded by title and abstract | | |
|  | | Hanley-Cook GT et al., 2020 | Minimum Dietary Diversity for Women of Reproductive Age (MDD-W) Data Collection: Validity of the List-Based and Open Recall Methods as Compared to Weighed Food Record | | <https://pubmed.ncbi.nlm.nih.gov/32659995/> | | excluded by title and abstract | | |
|  | | Hanley-Cook G et al., 2022 | Fortified Balanced Energy-Protein Supplementation, Maternal Anemia, and Gestational Weight Gain: A Randomized Controlled Efficacy Trial among Pregnant Women in Rural Burkina Faso | | <https://pubmed.ncbi.nlm.nih.gov/35906874/> | | excluded by title and abstract | | |
|  | | Hanley-Cook GT et al., 2022 | Seasonality and Day-to-Day Variability of Dietary Diversity: Longitudinal Study of Pregnant Women Enrolled in a Randomized Controlled Efficacy Trial in Rural Burkina Faso | | <https://pubmed.ncbi.nlm.nih.gov/35524695/> | | excluded by title and abstract | | |
|  | | Hailu S et al., 2019 | Low immunization coverage in Wonago district, southern Ethiopia: A community-based cross-sectional study | | <https://pubmed.ncbi.nlm.nih.gov/31339939/> | | excluded by title and abstract | | |
|  | | Hailu BA et al., 2021 | Subnational mapping for targeting anaemia prevention in women of reproductive age in Ethiopia: A coverage-equity paradox | | <https://pubmed.ncbi.nlm.nih.gov/34624171/> | | excluded by title and abstract | | |
|  | | Haile DT et al., 2022 | Vitamin D deficiency and associated factors among antenatal care attending pregnant women in Sodo town, South Ethiopia: A facility-based cross-sectional study | | <https://pubmed.ncbi.nlm.nih.gov/36584237/> | | excluded by title and abstract | | |
|  | | Habibzadeh N et al., 1986 | The effects of maternal folic acid and vitamin C nutrition in early pregnancy on reproductive performance in the guinea-pig | | <https://pubmed.ncbi.nlm.nih.gov/3663576/> | | excluded by title and abstract | | |
|  | | Guyon AB et al., 2009 | Implementing an integrated nutrition package at large scale in Madagascar: the Essential Nutrition Actions framework | | <https://pubmed.ncbi.nlm.nih.gov/19927603/> | | excluded by title and abstract | | |
|  | | Gunaratna NS et al., 2015 | Multivitamin and iron supplementation to prevent periconceptional anemia in rural Tanzanian women: a randomized, controlled trial | | <https://pubmed.ncbi.nlm.nih.gov/25905863/> | | excluded by title and abstract | | |
|  | | Gray RH et al., 2001 | Randomized trial of presumptive sexually transmitted disease therapy during pregnancy in Rakai, Uganda | | <https://www.embase.com/search/results?subaction=viewrecord&id=L33097389&from=export> | | excluded by title and abstract | | |
|  | | Grant FK et al., 2022 | Association between infection and nutritional status among infants in a cohort study of vitamin A in western Kenya | | <https://pubmed.ncbi.nlm.nih.gov/36211493/> | | excluded by title and abstract | | |
|  | | Granot M et al., 1996 | Pregnancy and delivery practices and beliefs of Ethiopian immigrant women in Israel | | <https://pubmed.ncbi.nlm.nih.gov/8693724/> | | excluded by title and abstract | | |
|  | | Gomo E et al., 2004 | Maternal fecal microbiome predicts gestational age, birth weight and neonatal growth in rural Zimbabwe | | <https://www.embase.com/search/results?subaction=viewrecord&id=L2013033832&from=export> | | excluded by title and abstract | | |
|  | | Gomo E et al., 2003 | Reference values and predictors of white blood cell subset counts: A cross-sectional study among HIV seronegative pregnant women in Zimbabwe | | <https://www.embase.com/search/results?subaction=viewrecord&id=L36332053&from=export> | | excluded by title and abstract | | |
|  | | Godha D et al., 2022 | The Association between Iron and Folic Acid Supplementation and Malaria Prophylaxis and Linear Growth among Children and Neonatal Mortality in Sub-Saharan Africa—A Pooled Analysis | | <https://www.scopus.com/inward/record.uri?eid=2-s2.0-85141607259&doi=10.3390%2fnu14214496&partnerID=40&md5=4f484963e19b6359a617bebd5f3050d3> | | excluded by title and abstract | | |
|  | | Gies S et al., 2021 | Effects of weekly iron and folic acid supplements on malaria risk in nulliparous women in Burkina Faso: A periconceptional, double-blind, randomized controlled noninferiority trial | | <https://www.scopus.com/inward/record.uri?eid=2-s2.0-85055131291&doi=10.1093%2finfdis%2fjiy257&partnerID=40&md5=5ab48dec3957fd94fa8a9e4500a4c3e1> | | excluded by title and abstract | | |
|  | | Hanley-Cook GT et al., 2022 | Seasonality and Day-to-Day Variability of Dietary Diversity: Longitudinal Study of Pregnant Women Enrolled in a Randomized Controlled Efficacy Trial in Rural Burkina Faso | | <https://pubmed.ncbi.nlm.nih.gov/35524695/> | | excluded by title and abstract | | |
|  | | Hailu S et al., 2019 | Low immunization coverage in Wonago district, southern Ethiopia: A community-based cross-sectional study | | <https://pubmed.ncbi.nlm.nih.gov/31339939/> | | excluded by title and abstract | | |
|  | | Hailu BA et al., 2021 | Subnational mapping for targeting anaemia prevention in women of reproductive age in Ethiopia: A coverage-equity paradox | | <https://pubmed.ncbi.nlm.nih.gov/34624171/> | | excluded by title and abstract | | |
|  | | Haile DT et al., 2022 | Vitamin D deficiency and associated factors among antenatal care attending pregnant women in Sodo town, South Ethiopia: A facility-based cross-sectional study | | <https://pubmed.ncbi.nlm.nih.gov/36584237/> | | excluded by title and abstract | | |
|  | | Habibzadeh N et al., 1986 | The effects of maternal folic acid and vitamin C nutrition in early pregnancy on reproductive performance in the guinea-pig | | <https://pubmed.ncbi.nlm.nih.gov/3663576/> | | excluded by title and abstract | | |
|  | | Gunaratna NS et al., 2015 | Multivitamin and iron supplementation to prevent periconceptional anemia in rural Tanzanian women: a randomized, controlled trial | | <https://pubmed.ncbi.nlm.nih.gov/25905863/> | | excluded by title and abstract | | |
|  | | Gregory EF et al., 2021 | Preconception and interconception pediatric primary care utilization of pregnant and parenting teens | | <https://pubmed.ncbi.nlm.nih.gov/676013146/> | | excluded by title and abstract | | |
|  | | Gibson RS et al., 2008 | Zinc, gravida, infection, and iron, but not vitamin B-12 or folate status, predict hemoglobin during pregnancy in Southern Ethiopia | | <https://pubmed.ncbi.nlm.nih.gov/18287370/> | | excluded by title and abstract | | |
|  | | Geze Tenaw S, Girma Fage S, Assefa N, Kenay Tura A, 2021 | Determinants of maternal near-miss in private hospitals in eastern Ethiopia: A nested case-control study | | <https://pubmed.ncbi.nlm.nih.gov/34844476/> | | excluded by title and abstract | | |
|  | | Geta TG, Gebremedhin S, Omigbodun AO, 2022 | Prevalence and predictors of anemia among pregnant women in Ethiopia: Systematic review and meta-analysis | | <https://pubmed.ncbi.nlm.nih.gov/35895619/> | | excluded by title and abstract | | |
|  | | Gernand AD et al., 2019 | Concurrent Micronutrient Deficiencies Are Low and Micronutrient Status Is Not Related to Common Health Indicators in Ghanaian Women Expecting to Become Pregnant | | <https://pubmed.ncbi.nlm.nih.gov/31187085/> | | excluded by title and abstract | | |
|  | | Gelagay AA et al., 2023 | Inadequate receipt of ANC components and associated factors among pregnant women in Northwest Ethiopia, 2020-2021: a community-based cross-sectional study | | <https://pubmed.ncbi.nlm.nih.gov/37143136/> | | excluded by title and abstract | | |
|  | | Geda NR et al., 2021 | Inequalities in adherence to the continuum of maternal and child health service utilization in Ethiopia: multilevel analysis | | <https://pubmed.ncbi.nlm.nih.gov/34717779/> | | excluded by title and abstract | | |
|  | | Gebreselassie SG, Gase FE, Deressa MU, 2013 | Prevalence and correlates of prenatal vitamin A deficiency in rural Sidama, Southern Ethiopia | | <https://pubmed.ncbi.nlm.nih.gov/23930336/> | | excluded by title and abstract | | |
|  | | Gebremedhin T et al., 2019 | Process evaluation of the community-based newborn care program implementation in Geze Gofa district, south Ethiopia: a case study evaluation design | | <https://pubmed.ncbi.nlm.nih.gov/31829193/> | | excluded by title and abstract | | |
|  | | Gebremedhin T et al., 2021 | Micronutrient intake status and associated factors among children aged 6-23 months in the emerging regions of Ethiopia: A multilevel analysis of the 2016 Ethiopia demographic and health survey | | <https://pubmed.ncbi.nlm.nih.gov/34679088/> | | excluded by title and abstract | | |
|  | | Gebremedhin S, Enquselassie F, Umeta M, 2012 | Independent and joint effects of prenatal Zinc and Vitamin A Deficiencies on birthweight in rural Sidama, Southern Ethiopia: prospective cohort study | | <https://pubmed.ncbi.nlm.nih.gov/23272058/> | | excluded by title and abstract | | |
|  | | Gebremedhin S, Enquselassie F, Umeta M, 2014 | Prevalence and correlates of maternal anemia in rural Sidama, Southern Ethiopia | | <https://pubmed.ncbi.nlm.nih.gov/24796168/> | | excluded by title and abstract | | |
|  | | Gebre-Medhin M, Vahlquist A, 1984 | Vitamin A nutrition in the human foetus. A comparison of Sweden and Ethiopia | | <https://pubmed.ncbi.nlm.nih.gov/6540032/> | | excluded by title and abstract | | |
|  | | Gebre-Medhin M et al., 1976 | Rarity of anaemia of pregnancy in Ethiopia | | <https://pubmed.ncbi.nlm.nih.gov/1273511/> | | excluded by title and abstract | | |
|  | | Gbadegesin A et al., 2017 | Maternal serum vitamin D levels and pregnancy outcomes: from Lagos, Nigeria | | <https://pubmed.ncbi.nlm.nih.gov/27760474/> | | excluded by title and abstract | | |
|  | | Gaffer AA et al., 2019 | Vitamin D status in Sudanese pregnant women: a cross-sectional study | | <https://pubmed.ncbi.nlm.nih.gov/31271422/> | | excluded by title and abstract | | |
|  | | Frumence G et al., 2019 | Factors affecting early identification of pregnant women by community health workers in Morogoro, Tanzania | | <https://pubmed.ncbi.nlm.nih.gov/31286930/> | | excluded by title and abstract | | |
|  | | Friis H et al., 2003 | Iron, haptoglobin phenotype, and HIV-1 viral load: A cross-sectional study among pregnant Zimbabwean women | | <https://www.embase.com/search/results?subaction=viewrecord&id=L36547244&from=export> | | excluded by title and abstract | | |
|  | | Friis H et al., 2004 | Effect of multimicronutrient supplementation on gestational length and birth size: A randomized, placebo-controlled, double-blind effectiveness trial in Zimbabwe | | <https://www.embase.com/search/results?subaction=viewrecord&id=L41115572&from=export> | | excluded by title and abstract | | |
|  | | Friis H et al., 2001 | HIV and other predictors of serum β-carotene and retinol in pregnancy: A cross-sectional study in Zimbabwe | | <https://www.embase.com/search/results?subaction=viewrecord&id=L32496945&from=export> | | excluded by title and abstract | | |
|  | | Fondjo LA et al., 2021 | High prevalence of vitamin D deficiency among Ethiopian women immigrants to Israel: exacerbation during pregnancy and lactation | | <https://pubmed.ncbi.nlm.nih.gov/33902494/> | | excluded by title and abstract | | |
|  | | Fogelman Y et al., 1995 | High prevalence of vitamin D deficiency among Ethiopian women immigrants to Israel: exacerbation during pregnancy and lactation | | <https://pubmed.ncbi.nlm.nih.gov/7721559/> | | excluded by title and abstract | | |
|  | | Fleming AF, Hendrickse JP, Allan NC, 1968 | The prevention of megaloblastic anaemia in pregnancy in Nigeria | | <https://pubmed.ncbi.nlm.nih.gov/5653282/> | | excluded by title and abstract | | |
|  | | Fleming AF et al., 1984 | Anaemia in young primigravidae in the guinea savanna of Nigeria: sickle-cell trait gives partial protection against malaria | | <https://pubmed.ncbi.nlm.nih.gov/6383238/> | | excluded by title and abstract | | |
|  | | Fleming AF et al., 1986 | The prevention of anaemia in pregnancy in primigravidae in the guinea savanna of Nigeria | | <https://pubmed.ncbi.nlm.nih.gov/3530158/> | | excluded by title and abstract | | |
|  | | Fleming AF, Elliott BA, 1964 | SERUM ENZYME TESTS FOR MEGALOBLASTIC ERYTHROPOIESIS IN ANAEMIA IN PREGNANCY | | <https://pubmed.ncbi.nlm.nih.gov/14198725/> | | excluded by title and abstract | | |
|  | | Fleming AF, Allan NC, 1969 | Severe haemolytic anaemia in pregnancy in Nigerians treated with prednisolone | | <https://pubmed.ncbi.nlm.nih.gov/5354835/> | | excluded by title and abstract | | |
|  | | Fleming AF, 1989 | The aetiology of severe anaemia in pregnancy in Ndola, Zambia | | <https://www.embase.com/search/results?subaction=viewrecord&id=L19097429&from=export> | | excluded by title and abstract | | |
|  | | Fleming AF, 1982 | Iron deficiency in the tropics | | <https://pubmed.ncbi.nlm.nih.gov/7042157/> | | excluded by title and abstract | | |
|  | | Flax VL, Bose S, Escobar-DeMarco J, Frongillo EA, 2023 | Changing maternal, infant and young child nutrition practices through social and behaviour change interventions implemented at scale: Lessons learned from Alive & Thrive | | <https://pubmed.ncbi.nlm.nih.gov/37735818/> | | excluded by title and abstract | | |
|  | | Fite MB et al., 2023 | Factors associated with undernutrition among pregnant women in Haramaya district, Eastern Ethiopia: A community-based study | | <https://pubmed.ncbi.nlm.nih.gov/36893154/> | | excluded by title and abstract | | |
|  | | Fite MB et al., 2022 | Prevalence, predictors of low birth weight and its association with maternal iron status using serum ferritin concentration in rural Eastern Ethiopia: a prospective cohort study | | <https://pubmed.ncbi.nlm.nih.gov/35883202/> | | excluded by title and abstract | | |
|  | | Fischl et al 2010 | Impact of a preconception counseling program for teens with type 1 diabetes (READY-Girls) on patient-provider interaction, resource utilization, and cost | |  | | excluded by title and abstract | | |
|  | | Finkelstein JL et al., 2014 | Anemia, vitamin B12, and folate statuses during pregnancy, and their association with obstetric outcomes among HIV-infected Ugandan women receiving ART | | <https://www.embase.com/search/results?subaction=viewrecord&id=L71421259&from=export> | | excluded by title and abstract | | |
|  | | Finkelstein JL et al., 2012 | Predictors of anaemia and iron deficiency in HIV-infected pregnant women in Tanzania: a potential role for vitamin D and parasitic infections | | <https://pubmed.ncbi.nlm.gov/22014374/> | | excluded by title and abstract | | |
|  | | Finkelstein JL et al., 2012 | Maternal vitamin D status and child morbidity, anemia, and growth in human immunodeficiency virus-exposed children in Tanzania | | <https://pubmed.ncbi.nlm.gov/22252204/> | | excluded by title and abstract | | |
|  | | Finkelstein JL et al., 2020 | Anemia and Micronutrient Status during Pregnancy, and Their Associations with Obstetric and Infant Outcomes among HIV-Infected Ugandan Women Receiving Antiretroviral Therapy | | <https://www.embase.com/search/results?subaction=viewrecord&id=L632247693&from=export> | | excluded by title and abstract | | |
|  | | Finkelstein JL et al., 2024 | Vitamin B12 supplementation during pregnancy for maternal and child health outcomes | | <https://pubmed.ncbi.nlm.gov/38189492/> | | excluded by title and abstract | | |
|  | | Finch SL et al., 2010 | Postnatal vitamin D supplementation following maternal dietary vitamin D deficiency does not affect bone mass in weanling guinea pigs | | <https://pubmed.ncbi.nlm.gov/20660278/> | | excluded by title and abstract | | |
|  | | Fenta EH et al., 2020 | Landscape analysis of nutrition services at Primary Health Care Units (PHCUs) in four districts of Ethiopia | | <https://pubmed.ncbi.nlm.gov/33270764/> | | excluded by title and abstract | | |
|  | | Fazio-Tirrozzo G et al., 1998 | A community based study of vitamin A and vitamin E status of adolescent girls living in the Shire Valley, Southern Malawi | | <https://pubmed.ncbi.nlm.gov/9756119/> | | excluded by title and abstract | | |
|  | | Fawzi WW et al., 2005 | Trial of zinc supplements in relation to pregnancy outcomes, hematologic indicators, and T cell counts among HIV-1-infected women in Tanzania | | <https://pubmed.ncbi.nlm.gov/15640476> | | excluded by title and abstract | | |
|  | | Fawzi WW et al., 2003 | Effect of providing vitamin supplements to human immunodeficiency virus-infected, lactating mothers on the child's morbidity and CD4+ cell counts | | <https://pubmed.ncbi.nlm.nih.gov/12684919/> | | excluded by title and abstract | | |
|  | | Fawzi WW et al., 2007 | Vitamins and perinatal outcomes among HIV-negative women in Tanzania | | <https://pubmed.ncbi.nlm.nih.gov/17409323/> | | excluded by title and abstract | | |
|  | | Fawzi WW et al., 2004 | A randomized trial of multivitamin supplements and HIV disease progression and mortality | | <https://pubmed.ncbi.nlm.nih.gov/15229304/> | | excluded by title and abstract | | |
|  | | Fawzi WW et al., 1998 | Randomised trial of effects of vitamin supplements on pregnancy outcomes and T cell counts in HIV-1-infected women in Tanzania | | <https://www.embase.com/search/results?subaction=viewrecord&id=L28227039&from=export> | | excluded by title and abstract | | |
|  | | Fawzi WW et al., 1999 | Rationale and design of the Tanzania Vitamin and HIV Infection Trial | | <https://pubmed.ncbi.nlm.nih.gov/10027501/> | | excluded by title and abstract | | |
|  | | Fawzi WW et al., 2007 | Multivitamin supplementation improves hematologic status in HIV-infected women and their children in Tanzania | | <https://www.embase.com/search/results?subaction=viewrecord&id=L46740477&from=export> | | excluded by title and abstract | | |
|  | | Fawzi WW et al., 2002 | Randomized trial of vitamin supplements in relation to transmission of HIV-1 through breastfeeding and early child mortality | | <https://pubmed.ncbi.nlm.nih.gov/12351954/> | | excluded by title and abstract | | |
|  | | Fawzi WW, Msamanga GI, 1999 | Epidemiological studies of vitamins among HIV-infected women and children | | <https://www.embase.com/search/results?subaction=viewrecord&id=L29587034&from=export> | | excluded by title and abstract | | |
|  | | Fawzi WW et al., 2000 | Randomized trial of vitamin supplements in relation to vertical transmission of HIV-1 in Tanzania | | <https://www.embase.com/search/results?subaction=viewrecord&id=L30331243&from=export> | | excluded by title and abstract | | |
|  | | Fawzi WW et al., 2002 | Transmission of HIV-1 through breastfeeding among women in Dar es Salaam, Tanzania | | <https://www.embase.com/search/results?subaction=viewrecord&id=L35278635&from=export> | | excluded by title and abstract | | |
|  | | Fawzi W et al., 2001 | Predictors of intrauterine and intrapartum transmission of HIV-1 among Tanzanian women | | https://pubmed.ncbi.nlm.nih.gov/11416718/ | | excluded by title and abstract | | |
|  | | Fall CHD et al., 2009 | Multiple micronutrient supplementation during pregnancy in low-income countries: A meta-analysis of effects on birth size and length of gestation | | <https://www.embase.com/search/results?subaction=viewrecord&id=L358301838&from=export> | | excluded by title and abstract | | |
|  | | Eze UI et al., 2007 | Assessment of prescription profile of pregnant women visiting antenatal clinics | | <https://www.scopus.com/inward/record.uri?eid=2-s2.0-34648819305&doi=10.4321%2fS1886-36552007000300007&partnerID=40&md5=167278330d01f92b04fc5a535f3d4478> | | excluded by title and abstract | | |
|  | | Eyeberu A et al., 2023 | Vitamin A deficiency among pregnant women in Ethiopia: a systematic review and meta-analysis | | <https://pubmed.ncbi.nlm.nih.gov/37264928/> | | excluded by title and abstract | | |
|  | | Ettyang GA et al., 2005 | Assessment of body composition and breast milk volume in lactating mothers in pastoral communities in Pokot, Kenya, using deuterium oxide | | <https://pubmed.ncbi.nlm.nih.gov/15660228/> | | excluded by title and abstract | | |
|  | | Eshete A et al., 2021 | Magnitude and risk of neonatal death in neonatal intensive care unit at referral hospital in Godeo Zone: a prospective cohort study | | <https://pubmed.ncbi.nlm.nih.gov/33995807/> | | excluded by title and abstract | | |
|  | | Eriksen KG et al., 2020 | Effects of an Iodine-Containing Prenatal Multiple Micronutrient on Maternal and Infant Iodine Status and Thyroid Function: A Randomized Trial in The Gambia | | <https://pubmed.ncbi.nlm.nih.gov/32183608/> | | excluded by title and abstract | | |
|  | | Erba N et al., 2022 | Pregnancy Outcomes in Women With Mechanical Valve Prostheses Using Vitamin K Antagonist Therapy: The Experience of the Salam Centre for Cardiac Surgery in Sudan | | <https://www.embase.com/search/results?subaction=viewrecord&id=L2018293019&from=export> | | excluded by title and abstract | | |
|  | | Erba N et al., 2021 | Pregnancy in patients with mechanical heart valves: Foeto-maternal outcome in the population monitored by anticoagulation Service in Salam Centre for Cardiac Surgery-Emergency NGO-Khartoum-Sudan | | <https://www.embase.com/search/results?subaction=viewrecord&id=L637209655&from=export> | | excluded by title and abstract | | |
|  | | Engle-Stone R et al., 2019 | Replacing iron-folic acid with multiple micronutrient supplements among pregnant women in Bangladesh and Burkina Faso: costs, impacts, and cost-effectiveness | | <https://pubmed.ncbi.nlm.nih.gov/31134641/> | | excluded by title and abstract | | |
|  | | Engle-Stone R et al., 2011 | Plasma retinol-binding protein predicts plasma retinol concentration in both infected and uninfected Cameroonian women and children | | <https://pubmed.ncbi.nlm.nih.gov/22049292/> | | excluded by title and abstract | | |
|  | | Engidaw MT et al., 2023 | Micronutrient intake status and associated factors in children aged 6-23 months in sub-Saharan Africa | | <https://www.embase.com/search/results?subaction=viewrecord&id=L641652888&from=export> | | excluded by title and abstract | | |
|  | | Endeshaw M et al., 2016 | Obesity in young age is a risk factor for preeclampsia: a facility-based case-control study, northwest Ethiopia | | <https://pubmed.ncbi.nlm.nih.gov/27543276/> | | excluded by title and abstract | | |
|  | | Endeshaw M et al., 2015 | Diet and Pre-eclampsia: A Prospective Multicentre Case-Control Study in Ethiopia | | <https://pubmed.ncbi.nlm.nih.gov/25862389/> | | excluded by title and abstract | | |
|  | | Emejulu JK et al., 2011 | Peculiarities in cases of spina bifida cystica managed recently in south-east Nigeria: could antimalarial drugs be a major but unrecognized etiologic factor? | | <https://pubmed.ncbi.nlm.nih.gov/22301488/> | | excluded by title and abstract | | |
|  | | Turk. Arch, 2013 | 10th International Congress on Adolescent Health | | https://www.embase.com/search/results?subaction=viewrecord&id=L75000411&from=export U2 - L75000411 | | excluded by title and abstract | | |
|  | | Reproductive Health | Reproductive Health 2012 Scientific Abstracts | | <https://www.embase.com/search/results?subaction=viewrecord&id=L70845320&from=expor> | | excluded by title and abstract | | |
|  | | Elzain MA et al., 2014 | Hydrocephalus in Sudan diagnosis and management | | <https://www.embase.com/search/results?subaction=viewrecord&id=L71816462&from=export> | | excluded by title and abstract | | |
|  | | Elsen C et al., 2012 | Vitamins E, A and B(2) as Possible Risk Factors for Preeclampsia - under Consideration of the PROPER Study ("Prevention of Preeclampsia by High-Dose Riboflavin Supplementation") | | <https://pubmed.ncbi.nlm.nih.gov/25308984/> | | excluded by title and abstract | | |
|  | | Elom MO et al., 2019 | Maternal vitamin A supplementation delays time to first episode of parasitaemia and reduces malaria parasite densities among infants in rural communities in Ebonyi State, Nigeria | | <https://pubmed.ncbi.nlm.nih.gov/33597439/> | | excluded by title and abstract | | |
|  | | Elom MO et al., 2017 | Improved infant hemoglobin (Hb) and blood glucose concentrations: The beneficial effect of maternal vitamin A supplementation of malaria-infected mothers in Ebonyi State, Nigeria | | <https://pubmed.ncbi.nlm.nih.gov/27908227/> | | excluded by title and abstract | | |
|  | | Elnaeim AK et al., 2018 | Knowledge of women issues and epilepsy among doctors in Sudan | | <https://www.embase.com/search/results?subaction=viewrecord&id=L2000746069&from=export> | | excluded by title and abstract | | |
|  | | Elegbe I et al., 1984 | Traditional treatment of pregnancy anaemia in Nigeria. An indication for modern therapeutics | | <https://pubmed.ncbi.nlm.nih.gov/6495378/> | | excluded by title and abstract | | |
|  | | Elechi HA et al., 2021 | Vitamin D and bone mineral status of newborn-maternal pair delivering at a tertiary hospital in Nigeria | | <https://pubmed.ncbi.nlm.nih.gov/33723108/> | | excluded by title and abstract | | |
|  | | Ekpe AC et al., 2022 | Anaemia among Pregnant Women: Prevalence and Pattern at Booking Clinic of a Tertiary Health Care Facility in North Central Nigeria | | <https://pubmed.ncbi.nlm.nih.gov/35366666/> | | excluded by title and abstract | | |
|  | | Eke CB et al., 2016 | Epidemiology of congenital anomalies of the central nervous system in children in Enugu, Nigeria: A retrospective study | | <https://pubmed.ncbi.nlm.nih.gov/27549417/> | | excluded by title and abstract | | |
|  | | Eigbefoh JO et al., 2005 | How useful is the Helen Keller food frequency chart in the determination of the vitamin A status in pregnancy? | | <https://pubmed.ncbi.nlm.nih.gov/15814387/> | | excluded by title and abstract | | |
|  | | Dzando G et al., 2022 | The magnitude of anemia and preventive practices in mothers with children under five years of age in Dodi Papase, Volta region of Ghana | | <https://pubmed.ncbi.nlm.nih.gov/35984853/> | | excluded by title and abstract | | |
|  | | Dun-Dery F et al., 2021 | Uptake challenges of intermittent preventive malaria therapy among pregnant women and their health care providers in the Upper West Region of Ghana: A mixed-methods study | | <https://pubmed.ncbi.nlm.nih.gov/34632123/> | | excluded by title and abstract | | |
|  | | Duggan C et al., 2012 | Multiple micronutrient supplementation in Tanzanian infants born to HIV-infected mothers: A randomized, double-blind, placebo-controlled clinical trial | | <https://www.embase.com/search/results?subaction=viewrecord&id=L366102524&from=export> | | excluded by title and abstract | | |
|  | | Duckett JR, 1996 | Guidelines for dietary supplementation of pregnant women in a Rwandan refugee camp | | <https://pubmed.ncbi.nlm.nih.gov/8667322/> | | excluded by title and abstract | | |
|  | | Fall CHD et al., 2001 | Determinants of low birth weight among HIV-infected pregnant women in Tanzania | | <https://www.embase.com/search/results?subaction=viewrecord&id=L33101804&from=export> | | excluded by title and abstract | | |
|  | | Douamba Z et al., 2012 | Asymptomatic malaria correlates with anaemia in pregnant women at Ouagadougou, Burkina Faso | | <https://pubmed.ncbi.nlm.nih.gov/2322693> | | excluded by title and abstract | | |
|  | | Dop MC et al., 1992 | Anemia at delivery in Lome (Togo): prevalence, risk factors and consequences in newborn infants | | <https://pubmed.ncbi.nlm.nih.gov/1462033/> | | excluded by title and abstract | | |
|  | | Dim CC, Onah HE, 2007 | The prevalence of anemia among pregnant women at booking in Enugu, South Eastern Nigeria | | <https://pubmed.ncbi.nlm.nih.gov/18092018/> | | excluded by title and abstract | | |
|  | | Dickinson N et al., 2009 | A framework to explore micronutrient deficiency in maternal and child health in Malawi, Southern Africa | | <https://pubmed.ncbi.nlm.nih.gov/20102580/> | | excluded by title and abstract | | |
|  | | Dewelle WK et al., 2023 | Polymorphisms in Maternal Selected Folate Metabolism-Related Genes in Neural Tube Defect-Affected Pregnancy | | <https://pubmed.ncbi.nlm.nih.gov/37564453/> | | excluded by title and abstract | | |
|  | | Desta SA et al., 2020 | Maternal factors associated with low birth weight in public hospitals of Mekelle city, Ethiopia: a case-control study | | <https://pubmed.ncbi.nlm.nih.gov/32894179/> | | excluded by title and abstract | | |
|  | | Desta M et al., 2019 | Adherence of iron and folic acid supplementation and determinants among pregnant women in Ethiopia: a systematic review and meta-analysis | | <https://pubmed.ncbi.nlm.nih.gov/31864397/> | | excluded by title and abstract | | |
|  | | Deriba BS, Jemal K, 2021 | Determinants of Low Birth Weight Among Women Who Gave Birth at Public Health Facilities in North Shewa Zone: Unmatched Case-Control Study | | <https://pubmed.ncbi.nlm.nih.gov/34619995/> | | excluded by title and abstract | | |
|  | | Deriba BS, 2021 | Nutritional-Related Predictors of Preterm Birth in North Shewa Hospitals, Central Ethiopia: A Case-Control Study | | <https://pubmed.ncbi.nlm.nih.gov/34262390/> | | excluded by title and abstract | | |
|  | | Dereje I et al., 2021 | Prevalence of Anemia and Associated Factors Among Term Newborns in Nekemte Specialized Hospital, Western Ethiopia | | <https://pubmed.ncbi.nlm.nih.gov/34556995/> | | excluded by title and abstract | | |
|  | | Demilew YM, Asres Nigussie A, 2017 | Knowledge of Health Professionals on Folic Acid Use and Their Prescribing Practice in Bahir Dar City Administration, Northwest Ethiopia: Cross-Sectional Study | | <https://pubmed.ncbi.nlm.nih.gov/28135302/> | | excluded by title and abstract | | |
|  | | Delva W et al., 2010 | A Safe Motherhood project in Kenya: assessment of antenatal attendance, service provision and implications for PMTCT | | <https://pubmed.ncbi.nlm.nih.gov/20230571/> | | excluded by title and abstract | | |
|  | | Delgado-Suárez C et al., 2021 | Identifying areas for improvement in epilepsy management in developing countries: An experience of neurocooperation in Cameroon | | <https://pubmed.ncbi.nlm.nih.gov/31064664/> | | excluded by title and abstract | | |
|  | | de Kok B et al., 2022 | Prenatal fortified balanced energy-Protein supplementation and birth outcomes in rural Burkina Faso: A randomized controlled efficacy trial | | <https://www.scopus.com/inward/record.uri?eid=2-s2.0-85130420259&doi=10.1371%2fjournal.pmed.1004002&partnerID=40&md5=a4e22fb3f31b78471bc0b1323e24cff9> | | excluded by title and abstract | | |
|  | | de Kok B et al., 2021 | Fortified Balanced Energy-Protein Supplements Increase Nutrient Adequacy without Displacing Food Intake in Pregnant Women in Rural Burkina Faso | | <https://pubmed.ncbi.nlm.nih.gov/34494113/> | | excluded by title and abstract | | |
|  | | Deitchler M et al., 2004 | Lessons from successful micronutrient programs. Part I: Program initiation | | <https://www.embase.com/search/results?subaction=viewrecord&id=L38502741&from=export> | | excluded by title and abstract | | |
|  | | Dei-Adomakoh Y et al., 2014 | Second Trimester Anaemia in Pregnant Ghanaians | | <https://pubmed.ncbi.nlm.nih.gov/26445064/> | | excluded by title and abstract | | |
|  | | De Groulard M, Le Bras J, 1991 | Nutritional-Related Predictors of Preterm Birth in North Shewa Hospitals, Central Ethiopia: A Case-Control Study | | <https://pubmed.ncbi.nlm.nih.gov/34262390/> | | excluded by title and abstract | | |
|  | | Das S, Powers HJ, 1998 | Comparison of micronutrient intervention strategies in Ghana and Benin to cover micronutrient needs: Simulation of benefits and risks in women of reproductive age | | <https://www.scopus.com/inward/record.uri?eid=2-s2.0-85108875957&doi=10.3390%2fnu13072286&partnerID=40&md5=bfba9332c869fe2c8b1ce6ea925ab3b6> | | excluded by title and abstract | | |
|  | | Das JK et al., 2018 | Lipid-based nutrient supplements for maternal, birth, and infant developmental outcomes | | <https://pubmed.ncbi.nlm.nih.gov/30168868/> | | excluded by title and abstract | | |
|  | | Das S, Powers HJ, 1998 | The effects of maternal intake and gestational age on materno-fetal transport of vitamin C in the guinea-pig | | <https://pubmed.ncbi.nlm.nih.gov/9924271/> | | excluded by title and abstract | | |
|  | | Darling AM et al., 2017 | Vitamin A and zinc supplementation among pregnant women to prevent placental malaria: A randomized, double-blind, placebo-controlled trial in Tanzania | | <https://www.embase.com/search/results?subaction=viewrecord&id=L615289809&from=export> | | excluded by title and abstract | | |
|  | | Dairo MD et al., 2005 | HIV as an additional risk factor for anaemia in pregnancy: evidence from primary care level in Ibadan, Southwestern Nigeria | | <https://pubmed.ncbi.nlm.nih.gov/16749361/> | | excluded by title and abstract | | |
|  | | Dabis F et al., 1996 | Zidovudineto decrease mother-to-child transmission of HIV-I: A phase II study in West Africa. 1995-1996 (anrs 049a) | | <https://www.scopus.com/inward/record.uri?eid=2-s2.0-33748197838&partnerID=40&md5=5af77f0039dfa0ff49bad27f1b6b2a16> | | excluded by title and abstract | | |
|  | | Crider K., et al 2022 | Folic acid supplementation and malaria susceptibility and severity among people taking antifolate antimalarial drugs in endemic areas. | | <https://pubmed.ncbi.nlm.nih.gov/36321557/> | | excluded by title and abstract | | |
|  | | Coward WA, Paul AA, Prentice AM, 1984 | The impact of malnutrition on human lactation: observations from community studies | | <https://pubmed.ncbi.nlm.nih.gov/6427014/> | | excluded by title and abstract | | |
|  | | Coutsoudis A et al., 1999 | Randomized trial testing the effect of vitamin A supplementation on pregnancy outcomes and early mother-to-child HIV-1 transmission in Durban, South Africa | | <https://go.exlibris.link/9kJhV4pT> | | excluded by title and abstract | | |
|  | | Coutsoudis A et al., 2001 | Method of feeding and transmission of HIV-1 from mothers to children by 15 months of age: prospective cohort study from Durban, South Africa | | <https://pubmed.ncbi.nlm.nih.gov/11273218/> | | excluded by title and abstract | | |
|  | | Coulibaly SO et al., 2006 | Therapeutic efficacy of sulphadoxine-pyrimethamine and chloroquine for the treatment of uncomplicated malaria in pregnancy in Burkina Faso | | <https://www.scopus.com/inward/record.uri?eid=2-s2.0-33747079293&doi=10.1186%2f1475-2875-5-49&partnerID=40&md5=607db2c98577c8ad8b8264d62112bc47> | | excluded by title and abstract | | |
|  | | Coulibaly M et al., 1987 | Modifications of hemato-biological parameters in pregnant women in a migrating population in northern Cameroon: prevalence of anemia, iron and folates deficiencies | | <https://pubmed.ncbi.nlm.nih.gov/3654112/> | | excluded by title and abstract | | |
|  | | Cormick G et al., 2018 | Are women with history of pre-eclampsia starting a new pregnancy in good nutritional status in South Africa and Zimbabwe? | | <https://pubmed.ncbi.nlm.nih.gov/29907146/> | | excluded by title and abstract | | |
|  | | Conrad M.D. et al., 2017 | Impact of different malaria chemoprevention regimens for pregnant Ugandan women on plasmodium falciparum drug resistance-mediating polymorphisms | | <https://www.embase.com/search/results?subaction=viewrecord&id=L620730692&from=export> | | excluded by title and abstract | | |
|  | | Conradie C et al., 2023 | Maternal Nutrient Patterns Are Associated With Fetal Head Circumference And Femur Length: The Nuped Study | | <https://www.embase.com/search/results?subaction=viewrecord&id=L2023438552&from=export> | | excluded by title and abstract | | |
|  | | Conradie C et al., 2021 | A priori and a posteriori dietary patterns among pregnant women in Johannesburg, South Africa: The NUPED study | | <https://www.embase.com/search/results?subaction=viewrecord&id=L2005972357&from=export> | | excluded by title and abstract | | |
|  | | Compaore A et al., 2014 | "There is iron and iron…": Burkinabè women's perceptions of iron supplementation: a qualitative study | | <https://pubmed.ncbi.nlm.nih.gov/25138626/> | | excluded by title and abstract | | |
|  | | Compaoré A et al., 2018 | Community approval required for periconceptional adolescent adherence to weekly iron and/or folic acid supplementation: a qualitative study in rural Burkina Faso | | <https://pubmed.ncbi.nlm.nih.gov/29540225/> | | excluded by title and abstract | | |
|  | | Collomb H et al., 1967 | So-called nutritional neuropathies in Senegal | | <https://www.embase.com/search/results?subaction=viewrecord&id=L288078093&from=export> | | excluded by title and abstract | | |
|  | | Coker SJ et al., 2023 | Effects of Low Vitamin C Intake on Fertility Parameters and Pregnancy Outcomes in Guinea Pigs | | <https://hinari.summon.serialssolutions.com/2.0.0/link/0/eLvHCXMwrV1Nb9QwELVQ1QMXRFsQgYKMkOC0NLGd2D4uVReQQKxE4YYif6VEqE61mwj1wm9nJk6X3XLgQg6JlDiSM2N73ksmbwjh7HU-u7UmyMKYPHCvVemE8bJx3DJvc2Zh41VSYeSLz2x5jjk1m8pfmCKW1IKTHU94EYqmDCowpURgQjU-SMThiCykDWkxFlvcKuFgCXydJ3lSDjT_JA4Q6rQosIzsVkAadfv_Xp1vZ0xuhaDFfXJvwo50njp5QO6EeEiO5hF48-U1fUnHbM7xNfkR-ZZkide0a-iH7if92vbmso30lL6PvfkRaBfpAnOqEYbTpcEcLRTapCZ6ulyFCxTiuKafhh6GZFhTuPXtAIjU0GV7sX5AvizOzk_fzaZSCjMHCKufVZjuYhstvFONEKWSzlrZNAJiZLCeqzx4oXnuKqYK7rR3ulIB4ndhOCBAyR-SvdjF8IjQUhtdeJb7yjJhXdC5g6Nl0mrWeFNl5MWNPeurpJhRA9NAq9d_rJ6RN2jqTQtUuR5PgLPrydn1v5ydkVfoqBrnIrjFmemXAugoqlrVc6AOhZaAuDJyfOPLepqk6xrIYiUAwXGWkeebyzC98JuJiaEbxjYS_00uoY3aGQM7Xd-9Etvvo1A31lAWQPke_4-HfULuYql7zFVh7Jjs9ashPCX7WGV91T4bxzvsP_46-w1AMgwC> | | excluded by title and abstract | | |
|  | | Clermont A et al., 2018 | Acceptability and Utilization of Three Nutritional Supplements during Pregnancy: Findings from a Longitudinal, Mixed-Methods Study in Niger | | <https://pubmed.ncbi.nlm.nih.gov/30103529/> | | excluded by title and abstract | | |
|  | | Chunda-Liyoka C et al., 2020 | Healthy pregnancies and essential fats: Focus group discussions with Zambian women on dietary need and acceptability of a novel RUSF containing fish oil DHA | | <https://www.embase.com/search/results?subaction=viewrecord&id=L630858657&from=export> | | excluded by title and abstract | | |
|  | | Chinyanga EA et al., 2005 | Vitamin A status of term and preterm infants delivered at Harare Central Hospital and fed exclusively on breast milk | | <https://pubmed.ncbi.nlm.nih.gov/16892858/> | | excluded by title and abstract | | |
|  | | Chimhashu TL et al., 2024 | Comparison of test performance of two commonly used multiplex assays to measure micronutrient and inflammatory markers in serum: results from a survey among pregnant women in South Africa | | <https://pubmed.ncbi.nlm.nih.gov/37560803/> | | excluded by title and abstract | | |
|  | | Chikwati RP et al., 2019 | Maternal plasma vitamin D levels and associated determinants in late pregnancy in Harare, Zimbabwe: A cross-sectional study | | <https://www.embase.com/search/results?subaction=viewrecord&id=L628284017&from=export> | | excluded by title and abstract | | |
|  | | Cherniak W et al., 2014 | Outreach and portable ultrasound - A novel method of improving antenatal turnout, maternal health, and preventing mother to child transmission of HIV in rural Uganda | | <https://www.embase.com/search/results?subaction=viewrecord&id=L71820489&from=export> | | excluded by title and abstract | | |
|  | | Chandiwana NC et al., 2019 | Serum folate and birth outcomes: DTG vs EFV trial evidence in South Africa | | <https://www.embase.com/search/results?subaction=viewrecord&id=L634220983&from=export> | | excluded by title and abstract | | |
|  | | Chadewa J et al., 2020 | Group antenatal care: A baseline initiative to improve malaria in pregnancy and antenatal care attendance indicators. A case from Geita, Tanzania | | <https://www.embase.com/search/results?subaction=viewrecord&id=L637502419&from=export> | | excluded by title and abstract | | |
|  | | Centeno Tablante E et al., 2019 | Fortification of wheat and maize flour with folic acid for population health outcomes | | <https://pubmed.ncbi.nlm.nih.gov/31257574/> | | excluded by title and abstract | | |
|  | | Cave C et al., 2018 | A Comparison of Vitamin E Status and Associated Pregnancy Outcomes in Maternal-Infant Dyads between a Nigerian and a United States Population | | <https://pubmed.ncbi.nlm.nih.gov/30223433/> | | excluded by title and abstract | | |
|  | | Callaghan-Gillespie M et al., 2017 | Trial of ready-to-use supplemental food and corn-soy blend in pregnant Malawian women with moderate malnutrition: a randomized controlled clinical trial | | <https://pubmed.ncbi.nlm.nih.gov/28793991/> | | excluded by title and abstract | | |
|  | | Callea F., 2018 | The origin of mankind | | <https://www.embase.com/search/results?subaction=viewrecord&id=L627350564&from=expor>t | | excluded by title and abstract | | |
|  | | Byamugisha J et al., 2022 | The effect of blister packaging Iron and Folate on adherence to medication and hemoglobin levels among pregnant women at National Referral Hospital antenatal clinics in a low to middle income country: a Randomised Controlled Trial (The IFAd Trial) | | <https://www.embase.com/search/results?subaction=viewrecord&id=L2015203283&from=export> | | excluded by title and abstract | | |
|  | | Bwibo NO, 1985 | Birthweights of infants of teenage mothers in Nairobi | | <https://pubmed.ncbi.nlm.nih.gov/3868931/> | | excluded by title and abstract | | |
|  | | Brindle E et al., | Simultaneous assessment of iodine, iron, vitamin A, malarial antigenemia, and inflammation status biomarkers via a multiplex immunoassay method on a population of pregnant women from Niger | | <https://pubmed.ncbi.nlm.nih.gov/28982133/> | | excluded by title and abstract | | |
|  | | Brian-D Adinma JI et al., 2022 | Vitamin D and associated factors, among pregnant women in southeastern Nigeria | | <https://pubmed.ncbi.nlm.nih.gov/34486910/> | | excluded by title and abstract | | |
|  | | Brenner S et al., 2020 | Effect Heterogeneity in Responding to Performance-Based Incentives: A Quasi-Experimental Comparison of Impacts on Health Service Indicators Between Hospitals and Health Centers in Malawi | | <https://pubmed.ncbi.nlm.nih.gov/32521206/> | | excluded by title and abstract | | |
|  | | Brabin L et al., 2017 | Effects of long-term weekly iron and folic acid supplementation on lower genital tract infection - a double blind, randomised controlled trial in Burkina Faso | | <https://pubmed.ncbi.nlm.nih.gov/29166928/> | | excluded by title and abstract | | |
|  | | Brabin L et al., 1995 | Protecting adolescent girls against tetanus | | <https://pubmed.ncbi.nlm.nih.gov/7613395/> | | excluded by title and abstract | | |
|  | | Brabin BJ, van den Berg H, Nijmeyer F, 1986 | Folacin, cobalamin, and hematological status during pregnancy in rural Kenya: the influence of parity, gestation, and Plasmodium falciparum malaria | | <https://pubmed.ncbi.nlm.nih.gov/3518395/> | | excluded by title and abstract | | |
|  | | Brabin BJ et al., 2004 | Haematological profiles of the people of rural southern Malawi: an overview | | <https://pubmed.ncbi.nlm.nih.gov/15000734/> | | excluded by title and abstract | | |
|  | | Brabin BJ, 1985 | A comparison of maternal and foetal folacin and cobalamin activities at parturition in relation to Plasmodium falciparum infection | | <https://pubmed.ncbi.nlm.nih.gov/4095747/> | | excluded by title and abstract | | |
|  | | Brabin B et al., 2019 | Excess risk of preterm birth with periconceptional iron supplementation in a malaria endemic area: analysis of secondary data on birth outcomes in a double blind randomized controlled safety trial in Burkina Faso | | <https://pubmed.ncbi.nlm.nih.gov/31060615/> | | excluded by title and abstract | | |
|  | | Boye A et al., 2016 | Assessment of an aqueous seed extract of Parkia clappertoniana on reproductive performance and toxicity in rodents | | <https://pubmed.ncbi.nlm.nih.gov/26993051/> | | excluded by title and abstract | | |
|  | | Boulet M, 1997 | Micronutrient deficiencies. Reports from the field -- Africa | | <https://pubmed.ncbi.nlm.nih.gov/12290327/> | | excluded by title and abstract | | |
|  | | Bot GM et al., 2020 | Giant Encephalocele in Sokoto, Nigeria: A 5-Year Review of Operated Cases | | <https://pubmed.ncbi.nlm.nih.gov/32201291/> | | excluded by title and abstract | | |
|  | | Blum LS, Pelto GH, Pelto PJ, 2004 | Coping with a nutrient deficiency: cultural models of vitamin A deficiency in northern Niger | | <https://pubmed.ncbi.nlm.nih.gov/15370198/> | | excluded by title and abstract | | |
|  | | Bliznashka L et al., 2022 | Prenatal supplementation with multiple micronutrient supplements or medium-quantity lipid-based nutrient supplements has limited effects on child growth up to 24 months in rural Niger: a secondary analysis of a cluster randomized trial | | <https://pubmed.ncbi.nlm.nih.gov/34871344/> | | excluded by title and abstract | | |
|  | | Bitew ZW et al., 2020 | Magnitude and Associated Factors of Neural Tube Defects in Ethiopia: A Systematic Review and Meta-Analysis | | <https://pubmed.ncbi.nlm.nih.gov/32743026/> | | excluded by title and abstract | | |
|  | | Birhanu K et al., 2021 | Congenital Anomalies in Neonates Admitted to a Tertiary Hospital in Southwest Ethiopia: A Cross Sectional Study | | <https://pubmed.ncbi.nlm.nih.gov/35392332/> | | excluded by title and abstract | | |
|  | | Biracyaza E et al., 2021 | Regular antenatal care visits were associated with low risk of low birth weight among newborns in Rwanda: Evidence from the 2014/2015 Rwanda Demographic Health Survey (RDHS) Data | | <https://pubmed.ncbi.nlm.nih.gov/36406958/> | | excluded by title and abstract | | |
|  | | Bintabara D et al., 2019 | Adherence to standards of first-visit antenatal care among providers: A stratified analysis of Tanzanian facility-based survey for improving quality of antenatal care | | <https://pubmed.ncbi.nlm.nih.gov/31083696/> | | excluded by title and abstract | | |
|  | | Bikila H et al., 2023 | Prevalence and factors associated with adequate dietary diversity among pregnant women in Nekemte town, Western Ethiopia, 2021 | | <https://pubmed.ncbi.nlm.nih.gov/38162525/> | | excluded by title and abstract | | |
|  | | Bhutta, Zulfiqar A, Lassi, Zohra S, 2015 | Preconception care and nutrition interventions in low-and middle-income countries | |  | | Excluded by title | | |
|  | | Berti PR et al., 2010 | An adequacy evaluation of a 10-year, four-country nutrition and health programme | | <https://pubmed.ncbi.nlm.nih.gov/20202929/> | | excluded by title and abstract | | |
|  | | Berry GT, 1993 | Classic Galactosemia and Clinical Variant Galactosemia | | <https://pubmed.ncbi.nlm.nih.gov/20301691/> | | excluded by title and abstract | | |
|  | | Berihu BA et al., 2018 | High burden of neural tube defects in Tigray, Northern Ethiopia: Hospital-based study | | <https://pubmed.ncbi.nlm.nih.gov/30427877/> | | excluded by title and abstract | | |
|  | | Berhanu Z et al., 2019 | Predictors of inappropriate complementary feeding practice among children aged 6 to 23 months in Wonago District, South Ethiopia, 2017; case control study | | <https://pubmed.ncbi.nlm.nih.gov/31077158/> | | excluded by title and abstract | | |
|  | | Berhane A et al., 2022 | Effect of Picture-based health education and counselling on knowledge and adherence to preconception Iron-folic acid supplementation among women planning to be pregnant in Eastern Ethiopia: a randomized controlled trial | | <https://go.exlibris.link/KkRy0XxD> | | excluded by title and abstract | | |
|  | | Berhane A et al., 2023 | Determinants of neural tube defects among women who gave birth in hospitals in Eastern Ethiopia: evidence from a matched case control study | | <https://pubmed.ncbi.nlm.nih.gov/38071290/> | | excluded by title and abstract | | |
|  | | Berhane A, Belachew T, 2022 | Trend and burden of neural tube defects among cohort of pregnant women in Ethiopia: Where are we in the prevention and what is the way forward? | | <https://pubmed.ncbi.nlm.nih.gov/35180245/> | | excluded by title and abstract | | |
|  | | Belay E et al., 2018 | Hematological responses to iron-folate supplementation and its determinants in pregnant women attending antenatal cares in Mekelle City, Ethiopia | | <https://pubmed.ncbi.nlm.nih.gov/30273373/> | | excluded by title and abstract | | |
|  | | Bekele A et al., 2012 | Conjunctival impression cytology and detection of vitamin A deficiency in pregnant women, Gondar, Northwest Ethiopia | | <https://pubmed.ncbi.nlm.nih.gov/22519159/> | | excluded by title and abstract | | |
|  | | Bekela MB et al., 2020 | Determinants of Low Birth Weight among Newborns Delivered at Public Hospitals in Sidama Zone, South Ethiopia: Unmatched Case-Control Study | | <https://pubmed.ncbi.nlm.nih.gov/32351737/> | | excluded by title and abstract | | |
|  | | Baytekus A et al., 2019 | Clinical vitamin-A deficiency and associated factors among pregnant and lactating women in Northwest Ethiopia: a community-based cross-sectional study | | <https://pubmed.ncbi.nlm.nih.gov/31852468/> | | excluded by title and abstract | | |
|  | | Baylin A et al., 2005 | Effect of vitamin supplementation to HIV-infected pregnant women on the micronutrient status of their infants | | <https://www.embase.com/search/results?subaction=viewrecord&id=L41195213&from=export> | | excluded by title and abstract | | |
|  | | BAUMSLAG N, METZ J, 1964 | RESPONSE TO LETTUCE IN A PATIENT WITH MEGALOBLASTIC ANAEMIA ASSOCIATED WITH PREGNANCY | | <https://pubmed.ncbi.nlm.nih.gov/14194735/> | | excluded by title and abstract | | |
|  | | Baumslag N, 1976 | Trace metal studies in bushman hair | | <https://www.scopus.com/inward/record.uri?eid=2-s2.0-0017105418&doi=10.1080%2f00039896.1976.10667229&partnerID=40&md5=eb0863b8934b68cdb52270c1c2cca321> | | excluded by title and abstract | | |
|  | | Bationo F et al., 2023 | Folates in various African foods: Contents, food processing and matrix effects | | <https://www.scopus.com/inward/record.uri?eid=2-s2.0-85133551792&doi=10.1024%2f0300-9831%2fa000759&partnerID=40&md5=eda3e5e3a1fb78f47e5882304459e1c2> | | excluded by title and abstract | | |
|  | | Bates CJ et al., 2002 | Plasma carotenoid and vitamin E concentrations in women living in a rural west African (Gambian) community | | <https://pubmed.ncbi.nlm.nih.gov/12098880/> | | excluded by title and abstract | | |
|  | | Basu TK, 1985 | The conditioning effect of large doses of ascorbic acid in guinea pigs | | <https://pubmed.ncbi.nlm.nih.gov/4041985/> | | excluded by title and abstract | | |
|  | | Bastos-Moreira Y et al., 2023 | A Multi-Omics and Human Biomonitoring Approach to Assessing the Effectiveness of Fortified Balanced Energy–Protein Supplementation on Maternal and Newborn Health in Burkina Faso: A Study Protocol | | <https://www.scopus.com/inward/record.uri?eid=2-s2.0-85172917381&doi=10.3390%2fnu15184056&partnerID=40&md5=295bec0c88734e2da49b9c7a3bd8a3f1> | | excluded by title and abstract | | |
|  | | Bastian H, 2008 | Lucy Wills (1888-1964): the life and research of an adventurous independent woman | | <https://www.embase.com/search/results?subaction=viewrecord&id=L550079884&from=export> | | excluded by title and abstract | | |
|  | | Barnea ER et al., 1993 | Effect of xenobiotics on quinone reductase activity in first trimester explants | | <https://pubmed.ncbi.nlm.nih.gov/8458909/> | | excluded by title and abstract | | |
|  | | Barlow-Mosha LN et al., 2023 | Effect of dolutegravir on folate, vitamin B12 and mean corpuscular volume levels among children and adolescents with HIV: a sub-study of the ODYSSEY randomized controlled trial | | <https://www.scopus.com/inward/record.uri?eid=2-s2.0-85173649106&doi=10.1002%2fjia2.26174&partnerID=40&md5=43d31a0092b4e9e940fce894b286ec7e> | | excluded by title and abstract | | |
|  | | Bantie B et al., 2023 | Deworming utilization among pregnant mothers with at least one antenatal care follow-up in Ethiopia, 2022: A multilevel analysis | | <https://pubmed.ncbi.nlm.nih.gov/36662678/> | | excluded by title and abstract | | |
|  | | Bancha B et al., 2022 | Time to non-adherence to iron and folic acid supplementation and associated factors among pregnant women in Hosanna town, South Ethiopia: Cox-proportional hazard model | | <https://pubmed.ncbi.nlm.nih.gov/36149913/> | | excluded by title and abstract | | |
|  | | Bambo GM et al., 2023 | Postpartum anemia and its determinant factors among postnatal women in two selected health institutes in Gondar, Northwest Ethiopia: A facility-based, cross-sectional study | | <https://pubmed.ncbi.nlm.nih.gov/37153091/> | | excluded by title and abstract | | |
|  | | Baloyi SM, Booi MA, 2022 | Prevalence of congenital birth defects (CBD) and associated risk-factors of babies delivered at Universitas Academic Hospital in Bloemfontein South Africa: 2013-2017 | | <https://www.embase.com/search/results?subaction=viewrecord&id=L2023394735&from=export> | | excluded by title and abstract | | |
|  | | Balogun H et al., 2019 | Association of Sunlight Exposure and Consumption of Vitamin D-Rich Foods During Pregnancy with Adverse Birth Outcomes in an African Population | | <https://pubmed.ncbi.nlm.nih.gov/30690592/> | | excluded by title and abstract | | |
|  | | BALESTRI F, 1960 | Rivista di ostetricia e ginecologia | | <https://pubmed.ncbi.nlm.nih.gov/13686318/> | | excluded by title and abstract | | |
|  | | Baker SJ, DeMaeyer EM, 1979 | Nutritional anemia: its understanding and control with special reference to the work of the World Health Organization | | <https://pubmed.ncbi.nlm.nih.gov/369352/> | | excluded by title and abstract | | |
|  | | Azupogo F et al., 2024 | Ten2Twenty-Ghana: a randomised controlled trial on the efficacy of multiple micronutrient-fortified biscuits on the micronutrient status of adolescent girls | | <https://pubmed.ncbi.nlm.nih.gov/37795629/> | | excluded by title and abstract | | |
|  | | Azaare J et al., 2022 | Evaluating the impact of maternal health care policy on stillbirth and perinatal mortality in Ghana; a mixed method approach using two rounds of Ghana demographic and health survey data sets and qualitative design technique | | <https://pubmed.ncbi.nlm.nih.gov/36174023/> | | excluded by title and abstract | | |
|  | | Ayala D et al., 2021 | Magnitude and factors associated with surgical site infection among mothers underwent cesarean delivery in Nekemte town public hospitals, western Ethiopia | | <https://pubmed.ncbi.nlm.nih.gov/33905428/> | | excluded by title and abstract | | |
|  | | Avidime O et al., 2022 | Physiological Changes in Serum Calcium, Phosphate, Vitamin D, Parathyroid Hormone and Calcitonin During Pregnancy and Lactation in Randomised Population of Zaria Women | | <https://pubmed.ncbi.nlm.nih.gov/35947844/> | | excluded by title and abstract | | |
|  | | Avidime O et al., 2023 | Influence of pregnancy and lactation on vitamin D serum levels and antioxidant status in randomized women in Zaria | | <https://pubmed.ncbi.nlm.nih.gov/38243359/> | | excluded by title and abstract | | |
|  | | Assefa N et al., 2021 | Reported barriers to healthcare access and service disruptions caused by COVID-19 in Burkina Faso, Ethiopia, and Nigeria: A telephone survey | | <https://www.scopus.com/inward/record.uri?eid=2-s2.0-85112005680&doi=10.4269%2fajtmh.20-1619&partnerID=40&md5=6658c764197aa1cdd035bc0e8cd65548> | | excluded by title and abstract | | |
|  | | Ashorn P et al., 2015 | Supplementation of Maternal Diets during Pregnancy and for 6 Months Postpartum and Infant Diets Thereafter with Small-Quantity Lipid-Based Nutrient Supplements Does Not Promote Child Growth by 18 Months of Age in Rural Malawi: A Randomized Controlled Trial | | <https://pubmed.ncbi.nlm.nih.gov/25926413/> | | excluded by title and abstract | | |
|  | | Ashorn P et al., 2015 | The impact of lipid-based nutrient supplement provision to pregnant women on newborn size in rural Malawi: a randomized controlled trial | | <https://pubmed.ncbi.nlm.nih.gov/25646337/> | | excluded by title and abstract | | |
|  | | Ashebir G et al., 2022 | Determinants of hyperemesis gravidarum among pregnant women attending health care service in public hospitals of Southern Ethiopia | | <https://pubmed.ncbi.nlm.nih.gov/35472152/> | | excluded by title and abstract | | |
|  | | Asayehu TT et al., 2017 | Dietary behaviour, food and nutrient intake of women do not change during pregnancy in Southern Ethiopia | | <https://pubmed.ncbi.nlm.nih.gov/27373896/> | | excluded by title and abstract | | |
|  | | Armstrong Schellenberg JR et al., 2008 | Health and survival of young children in southern Tanzania | | <https://pubmed.ncbi.nlm.nih.gov/18522737/> | | excluded by title and abstract | | |
|  | | Arimond M et al., 2015 | Considerations in developing lipid-based nutrient supplements for prevention of undernutrition: Experience from the International Lipid-Based Nutrient Supplements (iLiNS) Project | | <https://www.scopus.com/inward/record.uri?eid=2-s2.0-84953861498&doi=10.1111%2fmcn.12049&partnerID=40&md5=c98d4195cfb8c852bd2d3637c47ce2ab> | | excluded by title and abstract | | |
|  | | Argaw A et al., 2023 | Fortified balanced energy–protein supplementation during pregnancy and lactation and infant growth in rural Burkina Faso: A 2 × 2 factorial individually randomized controlled trial | | <https://www.scopus.com/inward/record.uri?eid=2-s2.0-85148773551&doi=10.1371%2fjournal.pmed.1004186&partnerID=40&md5=e5ae268ec0c498881893a68f695a0c17> | | excluded by title and abstract | | |
|  | | Apgar J et al., 1995 | Marginal vitamin A intake during pregnancy in Guinea pigs: effect on immune parameters in neonate | | <https://go.exlibris.link/Vq9XTktY> | | excluded by title and abstract | | |
|  | | Apgar J et al., 1991 | Reduced survival of neonates due to vitamin A deficiency during pregnancy in the guinea pig | | <https://pubmed.ncbi.nlm.nih.gov/2020671/> | | excluded by title and abstract | | |
|  | | Anyebuno M et al., 1993 | Neural tube defects at Korle Bu Teaching Hospital, Accra, Ghana | | <https://pubmed.ncbi.nlm.nih.gov/8181439/> | | excluded by title and abstract | | |
|  | | Anyanwu O et al. | The Role of Household Assets in Improving Women's Dietary Diversity in Ethiopia | | <https://pubmed.ncbi.nlm.nih.gov/35391901/> | | excluded by title and abstract | | |
|  | | Antelman G et al., 2000 | Nutritional factors and infectious disease contribute to anemia among pregnant women with human immunodeficiency virus in Tanzania | | <https://pubmed.ncbi.nlm.nih.gov/10917907/> | | excluded by title and abstract | | |
|  | | Anetor JI et al., 2010 | Depressed antioxidant status in pregnant women on iron supplements: pathologic and clinical correlates | | <https://pubmed.ncbi.nlm.nih.gov/19812902/> | | excluded by title and abstract | | |
|  | | Andersen GS et al., 2010 | Effects of maternal micronutrient supplementation on fetal loss and under-2-years child mortality: long-term follow-up of a randomised controlled trial from Guinea-Bissau | | <https://pubmed.ncbi.nlm.nih.gov/21243915/> | | excluded by title and abstract | | |
|  | | Anane-Fenin B et al., 2023 | Prevalence, Pattern, and Outcome of Congenital Anomalies Admitted to a Neonatal Unit in a Low-Income Country-a Ten-Year Retrospective Study | | <https://pubmed.ncbi.nlm.nih.gov/36853373/> | | excluded by title and abstract | | |
|  | | Amuna P et al., 2012 | Impact of locally made food multimix on maternal weight gain and outcome of pregnancy in Gauteng province, South Africa | | <https://www.embase.com/search/results?subaction=viewrecord&id=L71002288&from=export> | | excluded by title and abstract | | |
|  | | Ampofo GD et al., 2022 | Malaria in pregnancy control and pregnancy outcomes: a decade's overview using Ghana's DHIMS II data | | <https://pubmed.ncbi.nlm.nih.gov/36303165/> | | excluded by title and abstract | | |
|  | | Amouzou A et al., 2012 | Reduction in child mortality in Niger: a Countdown to 2015 country case study | | <https://pubmed.ncbi.nlm.nih.gov/22999428/> | | excluded by title and abstract | | |
|  | | Amegah AK et al., 2022 | Vitamin D intake modifies the association of household air pollution exposure with maternal disorders of pregnancy | | <https://pubmed.ncbi.nlm.nih.gov/348374> | | excluded by title and abstract | | |
|  | | Amegah AK et al., 2018 | What factors influence dietary and non-dietary vitamin D intake among pregnant women in an African population? | | <https://pubmed.ncbi.nlm.nih.gov/29522981/> | | excluded by title and abstract | | |
|  | | Amblard J, 1990 | [Iron deficiency and pregnancy. A survey in a Mother and Child Care Center in Niamey (Niger)] | | <https://pubmed.ncbi.nlm.nih.gov/2263184/> | | excluded by title and abstract | | |
|  | | Altigani M, 1992 | The role of the village midwives in antenatal care services in the Sudan | | <https://www.embase.com/search/results?subaction=viewrecord&id=L22072296&from=export> | | excluded by title and abstract | | |
|  | | Al-Shafei AI et al., 2021 | Maternal early pregnancy serum level of 25-Hydroxyvitamin D and risk of gestational diabetes mellitus | | <https://www.embase.com/search/results?subaction=viewrecord&id=L2007096807&from=export> | | excluded by title and abstract | | |
|  | | Almekinder J et al., 2000 | Evaluation of plasma retinol-binding protein as a surrogate measure for plasma retinol concentrations | | <https://pubmed.ncbi.nlm.nih.gov/10885491/> | | excluded by title and abstract | | |
|  | | Alemu B, Gashu D, 2020 | Association of maternal anthropometry, hemoglobin and serum zinc concentration during pregnancy with birth weight | | <https://pubmed.ncbi.nlm.nih.gov/31923646/> | | excluded by title and abstract | | |
|  | | Alaofè H et al., 2017 | Prevalence of anaemia, deficiencies of iron and vitamin A and their determinants in rural women and young children: a cross-sectional study in Kalalé district of northern Benin | | <https://pubmed.ncbi.nlm.nih.gov/28120735/> | | excluded by title and abstract | | |
|  | | Alamneh TS et al., 2023 | Determinants of anemia severity levels among children aged 6-59 months in Ethiopia: Multilevel Bayesian statistical approach | | <https://pubmed.ncbi.nlm.nih.gov/36914676/> | | excluded by title and abstract | | |
|  | | Akpan U et al., 2017 | Factors Influencing Antenatal Haematinics Prescription Behaviour of Physicians in Calabar, Nigeria | | <https://pubmed.ncbi.nlm.nih.gov/28507637/> | | excluded by title and abstract | | |
|  | | Akowuah JA et al., 2022 | Predictors of Anaemia Prevalence Among Ghanaian Pregnant Women: A Cross-Sectional Study | | <https://pubmed.ncbi.nlm.nih.gov/35510934/> | | excluded by title and abstract | | |
|  | | Akankwasa E et al., 2023 | Assessment of Safe Motherhood Health Service Coverage, Birth Defects Detection and Child Disability Prevention Using Lot Quality Assurance Sampling in Central Uganda | | <https://pubmed.ncbi.nlm.nih.gov/37529501> | | excluded by title and abstract | | |
|  | | Ajong AB et al., 2023 | Calcium supplementation in pregnancy: An analysis of potential determinants in an under-resourced setting | | <https://pubmed.ncbi.nlm.nih.gov/37796953/> | | excluded by title and abstract | | |
|  | | Ahmed SM et al., 2002 | Malaria parasitemia during delivery | | <https://pubmed.ncbi.nlm.nih.gov/12070548/> | | excluded by title and abstract | | |
|  | | Ahmed S et al., 2018 | A health facility based case-control study on determinants of low birth weight in Dassie town, Northeast Ethiopia: the role of nutritional factors | | <https://pubmed.ncbi.nlm.nih.gov/30400909/> | | excluded by title and abstract | | |
|  | | Ahmed SF et al., 2020 | Association of methylenetetrahydrofolate reductase C677T and reduced-f carrier-1 G80A gene polymorphism with preeclampsia in Sudanese women | | <https://pubmed.ncbi.nlm.nih.gov/32013623/> | | excluded by title and abstract | | |
|  | | Ahmed MA et al., 2023 | Maternal and neonatal umbilical cord serum levels of 25-hydroxy vitamin D among Sudanese women with normal pregnancy: a cross-sectional study | | <https://www.embase.com/search/results?subaction=viewrecord&id=L2028780493&from=export> | | excluded by title and abstract | | |
|  | | Agyei EA et al., 2021 | Income Level but Not Nutrition Knowledge Is Associated with Dietary Diversity of Rural Pregnant Women from Northern Ghana | | <https://pubmed.ncbi.nlm.nih.gov/34336275/> | | excluded by title and abstract | | |
|  | | Afisulahi AM et al., 2023 | HERBAL MEDICINE USE DURING PREGNANCY IN A FEDERAL TEACHING HOSPITAL IN NORTHERN NIGERIA- A CROSS-SECTIONAL STUDY | | <https://pubmed.ncbi.nlm.nih.gov/37978929/> | | excluded by title and abstract | | |
|  | | Adu-Afarwuah S et al., 2018 | Supplementation during pregnancy with small-quantity lipid-based nutrient supplements or multiple micronutrients, compared with iron and folic acid, increases women's urinary iodine concentration in semiurban Ghana: A randomized controlled trial | | <https://pubmed.ncbi.nlm.nih.gov/29210520/> | | excluded by title and abstract | | |
|  | | Adu-Afarwuah S et al., 2019 | Maternal and Infant Supplementation with Small-Quantity Lipid-Based Nutrient Supplements Increases Infants' Iron Status at 18 Months of Age in a Semiurban Setting in Ghana: A Secondary Outcome Analysis of the iLiNS-DYAD Randomized Controlled Trial | | <https://pubmed.ncbi.nlm.nih.gov/30624674/> | | excluded by title and abstract | | |
|  | | Adu-Afarwuah S et al., 2015 | impact of small-quantity lipid-based nutrient supplement on hemoglobin, iron status and biomarkers of inflammation in pregnant Ghanaian women. | | <https://pubmed.ncbi.nlm.nih.gov/26924599/> | | excluded by title and abstract | | |
|  | | Adu-Afarwuah S et al., 2017 | Impact of small-quantity lipid-based nutrient supplement on hemoglobin, iron status and biomarkers of inflammation in pregnant Ghanaian women | | <https://pubmed.ncbi.nlm.nih.gov/26924599/> | | excluded by title and abstract | | |
|  | | Adu-Afarwuah S et al., 2016 | Small-quantity, lipid-based nutrient supplements provided to women during pregnancy and 6 mo postpartum and to their infants from 6 mo of age increase the mean attained length of 18-mo-old children in semi-urban Ghana: a randomized controlled trial | | <https://pubmed.ncbi.nlm.nih.gov/2753463> | | excluded by title and abstract | | |
|  | | Adu-Afarwuah S et al., 2017 | Maternal Supplementation with Small-Quantity Lipid-Based Nutrient Supplements Compared with Multiple Micronutrients, but Not with Iron and Folic Acid, Reduces the Prevalence of Low Gestational Weight Gain in Semi-Urban Ghana: A Randomized Controlled Trial | | <https://pubmed.ncbi.nlm.nih.gov/28275100/> | | excluded by title and abstract | | |
|  | | Adu-Afarwuah S et al., 2021 | Consumption of multiple micronutrients or small-quantity lipid-based nutrient supplements containing iodine at the recommended dose during pregnancy, compared with iron and folic acid, does not affect women’s urinary iodine concentration in rural Malawi: a secondary outcome analysis of the iLiNS | | <https://go.exlibris.link/GkVHhrgX> | | excluded by title and abstract | | |
|  | | Adu-Afarwuah S et al., 2023 | Prevalence of morbidity symptoms among pregnant and postpartum women receiving different nutrient supplements in Ghana and Malawi: A secondary outcome analysis of two randomised controlled trials | | <https://pubmed.ncbi.nlm.nih.gov/37021807/> | | excluded by title and abstract | | |
|  | | Adu-Afarwuah S, 2020 | Impact of nutrient supplementation on maternal nutrition and child growth and development in Sub-Saharan Africa: the case of small-quantity lipid-based nutrient supplements | | <https://www.scopus.com/inward/record.uri?eid=2-s2.0-85097893325&doi=10.1111%2fmcn.12960&partnerID=40&md5=dffcd9d3b60231d53dc7ab320fc5f455> | | excluded by title and abstract | | |
|  | | Adu-Afarwuah S, 2018 | From the Field: Improving Fetal and Infant Growth in Vulnerable Populations | | <https://pubmed.ncbi.nlm.nih.gov/29734823/> | | excluded by title and abstract | | |
|  | | Adjei-Banuah NY et al., 2021 | Nutrition Knowledge is Associated With the Consumption of Iron Rich Foods: A Survey Among Pregnant Women From a Rural District in Northern Ghana | | <https://pubmed.ncbi.nlm.nih.gov/34483667/> | | excluded by title and abstract | | |
|  | | Adinma Joseph Ifeanyi Brian-D et al., 2020 | Vitamin D status and its influence on pregnancy outcomes amongst pregnant women in southeastern Nigeria | | <https://go.exlibris.link/n51l0VsM> | | excluded by title and abstract | | |
|  | | Adeleye AO et al., 2010 | Central nervous system congenital malformations in a developing country: issues and challenges against their prevention | | <https://pubmed.ncbi.nlm.nih.gov/20091041/> | | excluded by title and abstract | | |
|  | | Adams KP et al., 2018 | Ghanaian parents' perceptions of pre and postnatal nutrient supplements and their effects | | <https://pubmed.ncbi.nlm.nih.gov/29656569/> | | excluded by title and abstract | | |
|  | | Adams KP et al., 2020 | The impact of maternal supplementation during pregnancy and the first 6 months postpartum on the growth status of the next child born after the intervention period: Follow-up results from Bangladesh and Ghana | | <https://pubmed.ncbi.nlm.nih.gov/32026568/> | | excluded by title and abstract | | |
|  | | Adam, I. et al., 2011 | A perspective of the epidemiology of malaria and anaemia and their impact on maternal and perinatal outcomes in Sudan | | <https://www.embase.com/search/results?subaction=viewrecord&id=L361382672&from=export> | | excluded by title and abstract | | |
|  | | Abusharib, A.B., 2019 | Morphological patterns of anaemia among pregnant women from Sudan | | <https://www.embase.com/search/results?subaction=viewrecord&id=L630654226&from=export> | | excluded by title and abstract | | |
|  | | Abitew DB et al., 2020 | Predictors of relapse of acute malnutrition following exit from community-based management program in Amhara region, Northwest Ethiopia: An unmatched case-control study | | <https://pubmed.ncbi.nlm.nih.gov/32320426/> | | excluded by title and abstract | | |
|  | | Abitew DB et al., 2020 | Rural children remain more at risk of acute malnutrition following exit from community based management of acute malnutrition program in South Gondar Zone, Amhara Region, Ethiopia: a comparative cross-sectional study | | <https://pubmed.ncbi.nlm.nih.gov/32071802/> | | excluded by title and abstract | | |
|  | | Abioye AI et al., 2016 | Iron Supplementation Affects Hematologic Biomarker Concentrations and Pregnancy Outcomes among Iron-Deficient Tanzanian Women | | <https://pubmed.ncbi.nlm.nih.gov/27121530/> | | excluded by title and abstract | | |
|  | | Abebe S et al., 2021 | Risk factors associated with congenital anomalies among newborns in southwestern Ethiopia: A case-control study | | <https://pubmed.ncbi.nlm.nih.gov/33508017/> | | excluded by title and abstract | | |
|  | | Abebe MS et al., 2022 | Congenital Hydrocephalus and Associated Risk Factors: An Institution-Based Case-Control Study, Dessie Town, North East Ethiopia | | <https://pubmed.ncbi.nlm.nih.gov/35592592/> | | excluded by title and abstract | | |
|  | | Abebe H et al., 2014 | Consumption of vitamin A rich foods and dark adaptation threshold of pregnant women at Damot Sore District, Wolayita, Southern Ethiopia | | <https://pubmed.ncbi.nlm.nih.gov/25183928/> | | excluded by title and abstract | | |
|  | | Abebe GT et al., 2022 | Immediate postpartum anemia and associated factors among women admitted to maternity ward at public hospitals in Harari Regional State, Eastern Ethiopia: A facility-based cross-sectional study | | <https://pubmed.ncbi.nlm.nih.gov/36204254/> | | excluded by title and abstract | | |
|  | | Abdulkadir A et al., 2019 | Risk Factors for Rubella Transmission in Kuyu District, Ethiopia, 2018: A Case-Control Study | | <https://pubmed.ncbi.nlm.nih.gov/31636663/> | | excluded by title and abstract | | |
|  | | Abdul G et al., 2023 | Biomarkers of oxidative stress and its nexus with haemoglobin variants and adverse foeto-maternal outcome among women with preeclampsia in a Ghanaian population: A multi-centre prospective study | | <https://pubmed.ncbi.nlm.nih.gov/36996011/> | | excluded by title and abstract | | |
|  | | Abdisa DK et al., 2023 | Effect of community based nutritional education on knowledge, attitude and compliance to IFA supplementation among pregnant women in rural areas of southwest Ethiopia: a quasi experimental study | | <https://pubmed.ncbi.nlm.nih.gov/37794346/> | | excluded by title and abstract | | |
|  | | Abdelrahim II et al., 2009 | Anaemia, folate and vitamin B12 deficiency among pregnant women in an area of unstable malaria transmission in eastern Sudan | | <https://pubmed.ncbi.nlm.nih.gov/19027130/> | | excluded by title and abstract | | |
|  | | Abdelrahiem SK et al., 2022 | Association between maternal serum 25-hydroxyvitamin D concentrations and the risk of pre-eclampsia in central Sudan: A case-control study | | <https://www.embase.com/search/results?subaction=viewrecord&id=L2018201120&from=export> | | excluded by title and abstract | | |
|  | | Zelka MA et al., 2022 | Individual-level and community-level determinants of use of maternal health services in Northwest Ethiopia: a prospective follow-up study | | <https://pubmed.ncbi.nlm.nih.gov/36351731/> | | did not contain specific preconceptional folic acid intake data | | |
|  | | Yohannes Z et al., 2019 | Levels and correlates of knowledge and attitude on preconception care at public hospitals in Wolayita Zone South Ethiopia | |  | | did not contain specific preconceptional folic acid intake data | | |
|  | | Yismaw AE et al., 2022 | Iron-folic acid adherence and associated factors among pregnant women attending antenatal care at Metema District, Northwest Ethiopia | | <https://pubmed.ncbi.nlm.nih.gov/36466514/> | | did not contain specific preconceptional folic acid intake data | | |
|  | | Yesehak B et al., 2023 | Folic acid prescription practice for high-risk prevention of spina bifida at a tertiary care hospital in Addis Ababa, Ethiopia | |  | | Study: Mothers of children with spina bifida | | |
|  | | Yeboah A et al., 2022 | Maternal Dietary Iron and Folate Intake in the Third Trimester and Birth Outcomes: A Prospective Cohort Study at a Teaching Hospital in Accra, Ghana | | <https://pubmed.ncbi.nlm.nih.gov/35189588/> | | did not contain specific preconceptional folic acid intake data | | |
|  | | Yalew M et al., 2023 | Individual and contextual-level factors associated with iron-folic acid supplement intake during pregnancy in Ethiopia: a multi-level analysis | | <https://hinari.summon.serialssolutions.com/2.0.0/link/0/eLvHCXMwrV3JjtQwELXQCAEXxE6GAZkzhEm8hxtbCw5ICIazVV4CEa10qxeh-Qm-ecqJE9GABAduiZPYclW5lrj8ihDOnlXlLzqhgboKyjMAHYRjOlSualyUPkrt-ICt8_E1X3xiH85STs1c-SuliI1owSMdT4PTEFr8VOkEse1dRAvImMcwxKngchwkptgqbydoI6vpxIxRp1tUykaUaK7KCj1qXuoDqzSA92NLql8F3Sb-yfn8PYfy6r5fw_l3WC5_MlCLG-R69izpi3EKN8ml2N8iV97nvfPb5Me7-ewVhT7QlKSOmhlvy2XKHKK59A6FzLEYaPpLS9NBuLJN-MEUfBfoNhUCHf4q0q7fwbdIx7OOdL2JXxJ-xzm205RIv1p38JwCHbIW8yiQUVDukM-LN2ev3pa5GkPp0enblT54dAarqgUMPBvPMSSvMXz2DqLxTAYTFGvrZBJbieTVUqPnCbWvPerTqvb8LjnqV328Tyh6ObExkrXYiQh1Y5RyXIUGotARuynIk4kbdj2CbtghWDHKjryzyDs78M7qgrxMDJvfTIDZQwPKjc1yY_8mNwWRE7vnjtCoWCEDizgx3mBkFwQIB3ipRBPQ0QTFC3IyyYbNq39rU8WvpB2lLMjj-TGu27QZA31c7cd3OGt0jbO9N4rSPDDXFbqVtSiIORCygykePum7rwM2OFI6bcMjUZ7O8vgPNDz-HzR8QK6xYU2JsjYn5Gi32ceH5HKqA7_pHg0r8gKBTj97> | | did not contain specific preconceptional folic acid intake data | | |
|  | | Wilunda C et al., 2015 | Determinants of utilisation of antenatal care and skilled birth attendant at delivery in South West Shoa Zone, Ethiopia: a cross sectional study | |  | | reported data were insufficient to obtain a prevalence estimate | | |
|  | | Williams L et al., 2012 | Associations between preconception counseling and maternal behaviors before and during pregnancy | |  | | study area in USA | | |
|  | | Werler MM et al., 1993 | Periconceptional folic acid exposure and risk of occurrent neural tube defects | |  | | not observational | | |
|  | | Wegene MA et al., 2022 | Utilization of preconception care and associated factors in Hosanna Town, Southern Ethiopia | |  | |  | | |
|  | | Wana EW, 2020 | Predictors of prenatal iron folic acid supplement utilization in Wolaita, South Ethiopia: a community based cross-sectional study (quantitative and qualitative approach) | | <https://pubmed.ncbi.nlm.nih.gov/32334544/> | | reported data were insufficient to obtain a prevalence estimate | | |
|  | | van Eijk AM et al., 2006 | Use of antenatal services and delivery care among women in rural western Kenya: a community based survey | | <https://pubmed.ncbi.nlm.nih.gov/16597344/> | | did not contain specific preconceptional folic acid intake data | | |
|  | | Vallières F et al., 2013 | Where are the gaps in improving maternal and child health in Mauritania? the case for contextualised interventions: a cross sectional study | | <https://pubmed.ncbi.nlm.gov/23720704/> | | reported data were insufficient to obtain a prevalence estimate | | |
|  | | Umar AG et al., 2019 | Awareness and perception of preconception care among women in Usmanu Danfodiyo University Teaching Hospital Sokoto, North-Western Nigeria | |  | | did not contain specific preconceptional folic acid intake data | | |
|  | | Tsegai MB et al., 2023 | Knowledge, Attitude, and Practice Regarding Supplemental Iron and Folic Acid Amongst Women Delivering in Edaga-Hamus Community Hospital: A Cross-Sectional Study in Asmara, Eritrea | | <https://pubmed.ncbi.nlm.nih.gov/37881442/> | | did not contain specific preconceptional folic acid intake data | | |
|  | | Tinago CB et al., 2017 | Individual and structural environmental influences on utilization of iron and folic acid supplementation among pregnant women in Harare, Zimbabwe | | <https://www.embase.com/search/results?subaction=viewrecord&id=L611598102&from=export> | | did not contain specific preconceptional folic acid intake data | | |
|  | | Teshome F et al., 2022 | A survey on women’s awareness of iron and folic acid intake during preconception period and its associated factors in Manna District, Oromia region, Southwest Ethiopia | | <https://go.exlibris.link/TjVYLhd5> | | did not contain specific preconceptional folic acid intake data | | |
|  | | Tesema KF et al., 2021 | Knowledge of preconception healthcare and associated factors: a study among mothers in Jinka town, southern region, Ethiopia | |  | | did not contain specific preconceptional folic acid intake data | | |
|  | | Tegodan E et al., 2021 | Adherence to Iron and Folic Acid Supplements and Associated Factors Among Pregnant Mothers Attending ANC at Gulele Sub-City Government Health Centers in Addis Ababa, Ethiopia | | <https://pubmed.ncbi.nlm.nih.gov/34188456/> | | did not contain specific preconceptional folic acid intake data | | |
|  | | Tefera AA et al., 2023 | Adherence to iron and folate supplementation and associated factors among women attending antenatal care in public health facilities at Covid-19 pandemic in Ethiopia | | <https://pubmed.ncbi.nlm.nih.gov/36962981/> | | did not contain specific preconceptional folic acid intake data | | |
|  | | Taye B et al., 2015 | Factors associated with compliance of prenatal iron folate supplementation among women in Mecha district, Western Amhara: a cross-sectional study | | <https://pubmed.ncbi.nlm.nih.gov/26090001/> | | did not contain specific preconceptional folic acid intake data | | |
|  | | Tarekegn M et al., 2019 | Antenatal care and mothers' education improved iron-folic acid adherence at Denbiya district health centers, Northwest Ethiopia: using pills count method | | <https://pubmed.ncbi.nlm.nih.gov/31285822/> | | did not contain specific preconceptional folic acid intake data | | |
|  | | Tafere TE et al., 2018 | Providers adherence to essential contents of antenatal care services increases birth weight in Bahir Dar City Administration, north West Ethiopia: a prospective follow up study | | <https://pubmed.ncbi.nlm.nih.gov/30268132/> | | did not contain specific preconceptional folic acid intake data | | |
|  | | Tadesse AW et al., 2021 | Individual and community-level determinants of Iron-Folic Acid Intake for the recommended period among pregnant women in Ethiopia: A multilevel analysis | | <https://pubmed.ncbi.nlm.nih.gov/34296017/> | | did not contain specific preconceptional folic acid intake data | | |
|  | | Sori SA et al., 2021 | Knowledge of preconception care and associated factors among maternal health care providers working in urban public health institutions of Eastern Ethiopia | |  | | did not contain specific preconceptional folic acid intake data | | |
|  | | Solomon Y et al., 2021 | Adherence and associated factors to iron and folic acid supplementation among pregnant women attending antenatal care in public hospitals of Dire Dawa, Eastern Ethiopia | | <https://pubmed.ncbi.nlm.nih.gov/34514359/> | | did not contain specific preconceptional folic acid intake data | | |
|  | | Simuyemba MC et al., 2020 | A root cause analysis of sub-optimal uptake and compliance to iron and folic acid supplementation in pregnancy in 7 districts of Zambia | | <https://www.embase.com/search/results?subaction=viewrecord&id=L630520356&from=export> | | did not contain specific preconceptional folic acid intake data | | |
|  | | Shengrong F et al., 2022 | Influencing factors of intention to utilize preconception health care services among women with future pregnancy plan | |  | | did not contain specific preconceptional folic acid intake data | | |
|  | | Sharif ME et al., 2017 | Folic acid level and preterm birth among Sudanese women | | <https://pubmed.ncbi.nlm.nih.gov/29214043/> | | did not contain specific preconceptional folic acid intake data | | |
|  | | Setegn M, 2021 | Intention to use and its predictors towards preconception care utilization among reproductive age women in Mizan-Aman town, Bench-Sheko Zone, southwest Ethiopia, 2020: Based on Theory of planned behaviour | |  | | did not contain specific preconceptional folic acid intake data | | |
|  | | Setegn M, 2021 | What Women Do Before Pregnancy: A Preconception Care of Women in Mizan Aman town Southwest Ethiopia: A Mixed Study | |  | | did not contain specific preconceptional folic acid intake data | | |
|  | | Seifu CN et al., 2020 | Better-Educated, Older, or Unmarried Pregnant Women Comply Less with Iron-Folic Acid Supplementation in Southern Ethiopia | | <https://pubmed.ncbi.nlm.nih.gov/31230484/> | | did not contain specific preconceptional folic acid intake data | | |
|  | | Seck BC et al., 2008 | Determinants of compliance with iron supplementation among pregnant women in Senegal | | <https://www.embase.com/search/results?subaction=viewrecord&id=L351693801&from=export> | | did not contain specific preconceptional folic acid intake data | | |
|  | | Rosenberg KD et al., 2003 | Pregnancy intendedness and the use of periconceptional folic acid | |  | | out of SSA countries | | |
|  | | Ridwan N et al., 2021 | Adherence to iron folate supplementation and associated factors among pregnant women attending antenatal care at public hospitals in Jigjiga Town, Somali Region, Ethiopia 2020 | | <https://pubmed.ncbi.nlm.nih.gov/35096223/> | | did not contain specific preconceptional folic acid intake data | | |
|  | | Riang'a RM et al., 2020 | Implementation fidelity of nutritional counselling, iron and folic acid supplementation guidelines and associated challenges in rural Uasin Gishu County Kenya | | <https://pubmed.ncbi.nlm.nih.gov/33334353/> | | reported data were insufficient to obtain a prevalence estimate | | |
|  | | Regassa T et al., 2014 | Factors Affecting Prenatal Care Utilization in East Wollega Zone, Oromia Regional State, Ethiopia | |  | | reported data were insufficient to obtain a prevalence estimate | | |
|  | | Prentice AM, 1991 | Can maternal dietary supplements help in preventing infant malnutrition? | | <https://pubmed.ncbi.nlm.nih.gov/1957632/> | | did not contain specific preconceptional folic acid intake data | | |
|  | | Pobee RA et al., (Year not provided) | The prevalence of anemia and iron deficiency among pregnant Ghanaian women, a longitudinal study | |  | | reported data were insufficient to obtain a prevalence estimate | | |
|  | | Petraro P et al., 2013 | Determinants of anemia in postpartum HIV-negative women in Dar es Salaam, Tanzania | | <https://www.embase.com/search/results?subaction=viewrecord&id=L52552572&from=export> | | did not contain specific preconceptional folic acid intake data | | |
|  | | Owino W et al., 2021 | Socio-Cultural Factors Influencing Utilization of Preconception Care among Women Seeking Antenatal Care in Machakos Level 5 Hospital, Machakos County, Kenya | |  | | did not contain specific preconceptional folic acid intake data | | |
|  | | Osungbade KO et al., 2011 | Clinical audit of antenatal service provision in Nigeria | | <https://pubmed.ncbi.nlm.nih.gov/21476162/> | | Exclude by title and abstract | | |
|  | | Omer IM et al., 2016 | Research: Prevalence of neural tube defects Khartoum, Sudan August 2014-July 2015 | | <https://www.embase.com/search/results?subaction=viewrecord&id=L616658699&from=export> | | did not contain specific preconceptional folic acid intake data | | |
|  | | Okeyo DO et al., 2019 | Nutritional service needs of pregnant and lactating adolescent girls in Trans-Mara East Sub-County, Narok County: focus on access and utilization of nutritional advice and services | | <https://pubmed.ncbi.nlm.nih.gov/31277585/> | | wrong population | | |
|  | | Okemo J, 2018 | Difference in the level of utilization and determinants of preconception care among pregnant women in Aga Khan University Hospital and Maragua level four hospital | |  | | did not contain specific preconceptional folic acid intake data | | |
|  | | Oiye S et al., 2020 | The Influence of Antenatal Oral Iron and Folic Acid Side Effects on Supplementation Duration in Low-Resource Rural Kenya: A Cross-Sectional Study | | <https://pubmed.ncbi.nlm.nih.gov/32455019/> | | did not contain specific preconceptional folic acid intake data | | |
|  | | Ogundipe O et al., 2012 | Factors associated with prenatal folic acid and iron supplementation among 21,889 pregnant women in Northern Tanzania: a cross-sectional hospital-based study | | <https://pubmed.ncbi.nlm.nih.gov/22734580/> | | did not contain specific preconceptional folic acid intake data | | |
|  | | Odira CCH et al., 2020 | PRECONCEPTION CARE UTILIZATION AMONG CHILDBEARING WOMEN IN SOUTH-EAST NIGERIA | |  | | did not contain specific preconceptional folic acid intake data | | |
|  | | Obarisiagbon OE et al., 2020 | Folic acid usage and its associated factors among antenatal attendees in a tertiary health facility: Implications for child health | |  | | did not contain specific preconceptional folic acid intake data | | |
|  | | Nimwesiga C et al., 2021 | Adherence to Iron and Folic Acid Supplementation and Its Associated Factors among Pregnant Women Attending Antenatal Care at Bwindi Community Hospital, Western Uganda | | <https://pubmed.ncbi.nlm.nih.gov/34195259/> | |  | | |
|  | | Nepali G et al., 2017 | Knowledge and practice regarding preconception care among antenatal mothers | |  | | did not contain specific preconceptional folic acid intake data | | |
|  | | Ndungwa HN, 2021 | FACTORS ASSOCIATED WITH UTILIZATION OF PRECONCEPTION CARE AMONG WOMEN ATTENDING MOTHER AND CHILD HEALTH CARE IN KAPSABET REFERRAL HOSPITAL, NANDI COUNTY, KENYA | |  | | did not contain specific preconceptional folic acid intake data | | |
|  | | Ndou NP et al., 2023 | Factors Related to the Implementation of Preconception Care Recommendations in Selected Districts of Limpopo Province: A Qualitative Study | |  | | did not contain specific preconceptional folic acid intake data | | |
|  | | Ndiaye NF et al., 2018 | Folate Deficiency and Anemia Among Women of Reproductive Age (15-49 Years) in Senegal: Results of a National Cross-Sectional Survey | | <https://pubmed.ncbi.nlm.nih.gov/29129112/> | | did not contain specific preconceptional folic acid intake data | | |
|  | | Nasir BB et al., 2020 | Adherence to iron and folic acid supplementation and prevalence of anemia among pregnant women attending antenatal care clinic at Tikur Anbessa Specialized Hospital, Ethiopia | | <https://pubmed.ncbi.nlm.nih.gov/32365114/> | | did not contain specific preconceptional folic acid intake data | | |
|  | | Mutale P et al., 2017 | Knowledge and preconception care seeking practices among reproductive-age diabetic women in Zambia | |  | | did not contain specific preconceptional folic acid intake data | | |
|  | | Munyogwa M et al., 2023 | Prevalence of Oral Iron-Folic Acid Supplementation and Dietary Intakes Among Pregnant Women at Peri-Urban Areas of Dodoma City, Tanzania | | <https://www.embase.com/search/results?subaction=viewrecord&id=L2026059697&from=export> | | did not contain specific preconceptional folic acid intake data | | |
|  | | Msemo OA et al., 2018 | Prevalence and risk factors of preconception anemia: A community based cross sectional study of rural women of reproductive age in northeastern Tanzania | | <https://pubmed.ncbi.nlm.nih.gov/30562390/> | | did not contain specific preconceptional folic acid intake data | | |
|  | | Molla T et al., 2019 | Factors associated with adherence to iron folate supplementation among pregnant women in West Dembia district, northwest Ethiopia: a cross sectional study | | <https://pubmed.ncbi.nlm.nih.gov/30612583/> | | did not contain specific preconceptional folic acid intake data | | |
|  | | Mohammed MA et al., 2013 | Supplement Use Among Pregnant Women in Ethiopia: Prevalence and Predictors | | <https://pubmed.ncbi.nlm.nih.gov/30235523/> | |  | | |
|  | | MISAME Study Group et al., 2009 | Prenatal food supplementation fortified with multiple micronutrients increases birth length: a randomized controlled trial in rural Burkina Faso | |  | | wrong study design | | |
|  | | Mengistu T et al., 2023 | Compliance to iron folic acid supplementation and its associated factors among pregnant women attending Antenatal clinic in Wondo district: a cross-sectional study | | <https://pubmed.ncbi.nlm.nih.gov/37838766/> | | did not contain specific preconceptional folic acid intake data | | |
|  | | Mekonnen A et al., 2021 | Adherence to Iron with Folic Acid Supplementation Among Pregnant Women Attending Antenatal Care in Public Health Centers in Simada District, Northwest Ethiopia: Using Health Belief Model Perspective | | <https://pubmed.ncbi.nlm.nih.gov/33911855/> | | reported data were insufficient to obtain a prevalence estimate | | |
|  | | Mbule MA et al., 2013 | Determinants of anaemia among pregnant women in rural Uganda | | <https://www.embase.com/search/results?subaction=viewrecord&id=L563068568&from=export> | | Exclude by Abstract and title | | |
|  | | Mbhenyane X et al., 2017 | Compliance with the consumption of iron and folate supplements by pregnant women in Mafikeng local municipality, North West province, South Africa | | <https://pubmed.ncbi.nlm.nih.gov/29085393/> | | Exclude by Abstract and title | | |
|  | | Massawe SN et al., 1999 | Effectiveness of primary level antenatal care in decreasing anemia at term in Tanzania | | <https://pubmed.ncbi.nlm.nih.gov/10422902/> | | Exclude by Abstract and title | | |
|  | | Manniën J et al., 2014 | Factors associated with not using folic acid supplements preconceptionally | |  | | not done in SSA country | | |
|  | | Mamo TT et al., 2021 | Adherence to prenatal iron-folic acid supplementation and associated factors among pregnant women attending antenatal care services in Dilla town, South Ethiopia | | <https://pubmed.ncbi.nlm.nih.gov/36204490/> | | did not contain specific preconceptional folic acid intake data | | |
|  | | Maina-Gathigi L et al., 2013 | Utilization of folic acid and iron supplementation services by pregnant women attending an antenatal clinic at a regional referral hospital in Kenya | | <https://pubmed.ncbi.nlm.nih.gov/22907273/> | | did not contain specific preconceptional folic acid intake data | | |
|  | | Mabuza GN et al., 2021 | Prevalence of iron and folic acid supplements consumption and associated factors among pregnant women in Eswatini: a multicenter cross-sectional study | |  | | did not contain specific preconceptional folic acid intake data | | |
|  | | Lyoba WB et al., 2020 | Adherence to Iron-Folic Acid Supplementation and Associated Factors among Pregnant Women in Kasulu Communities in North-Western Tanzania | | <https://pubmed.ncbi.nlm.nih.gov/32566646/> | | did not contain specific preconceptional folic acid intake data | | |
|  | | Lunet N et al., 2008 | Adequacy of prenatal care as a major determinant of folic acid, iron, and vitamin intake during pregnancy | |  | | did not contain specific preconceptional folic acid intake data | | |
|  | | Levine NH et al., 2001 | Folic acid and preconceptional care | |  | | did not contain specific preconceptional folic acid intake data | | |
|  | | Lema EJ et al., 2023 | Prevalence of anemia and its associated factors among pregnant women in Ilala Municipality - Tanzania: Analytical cross-sectional study | | <https://pubmed.ncbi.nlm.nih.gov/37335672/> | | did not contain specific preconceptional folic acid intake data | | |
|  | | Kindie Z et al., 2022 | Prevalence of neural tube defects at Debre Berhan Referral Hospital, North Shewa, Ethiopia: A hospital based retrospective cross-section study | | <https://pubmed.ncbi.nlm.nih.gov/35108260/> | | wrong study design | | |
|  | | Kedir Obsa A et al., 2021 | Iron and Folic Acid Supplementation Compliance and Associated Factors among Pregnant Women Attending Antenatal Clinic in Shalla District, Southwest Ethiopia: A Cross-Sectional Study | | <https://pubmed.ncbi.nlm.nih.gov/33854798/> | | did not contain specific preconceptional folic acid intake data | | |
|  | | Kassa ZY et al., 2019 | Compliance with iron folic acid and associated factors among pregnant women through pill count in Hawassa city, South Ethiopia: a community based cross-sectional study | | <https://pubmed.ncbi.nlm.nih.gov/30736812/> | | reported data were insufficient to obtain a prevalence estimate | | |
|  | | Kassa A et al., 2018 | Women’s knowledge and associated factors on preconception care at Public Health Institution in Hawassa City, South Ethiopia | |  | | did not contain specific preconceptional folic acid intake data | | |
|  | | Kare AP et al., 2021 | Anemia among Pregnant Women Attending Ante Natal Care Clinic in Adare General Hospital, Southern Ethiopia: Prevalence and Associated Factors | | <https://pubmed.ncbi.nlm.nih.gov/34376992/> | | did not contain specific preconceptional folic acid intake data | | |
|  | | Kamau MW et al., 2018 | Compliance with Iron and folic acid supplementation (IFAS) and associated factors among pregnant women: results from a cross-sectional study in Kiambu County, Kenya | | <https://pubmed.ncbi.nlm.nih.gov/29720135/> | | reported data were insufficient to obtain a prevalence estimate | | |
|  | | Kamau M et al., 2018 | Counselling and knowledge on iron and folic acid supplementation (IFAS) among pregnant women in Kiambu County, Kenya: a cross-sectional study | | <https://pubmed.ncbi.nlm.nih.gov/32259021/> | | reported data were insufficient to obtain a prevalence estimate | | |
|  | | Kalipa Z et al., 2017 | Factors influencing adherence to folic acid and ferrous sulphate nutritional intake among pregnant teenagers in buffalo city municipality, South Africa | | <https://www.embase.com/search/results?subaction=viewrecord&id=L617579697&from=export> | | did not contain specific preconceptional folic acid intake data | | |
|  | | John SE et al., 2023 | The prevalence and risk factors associated with Iron, vitamin B12 and folate deficiencies in pregnant women: A cross-sectional study in Mbeya, Tanzania | | <https://pubmed.ncbi.nlm.nih.gov/37083656/> | | did not contain specific preconceptional folic acid intake data | | |
|  | | Ilboudo B et al., 2021 | Prevalence and factors associated with anaemia in pregnant women in cascades region of burkina faso in 2012 | | <https://www.scopus.com/inward/record.uri?eid=2-s2.0-85107806649&doi=10.11604%2fpamj.2021.38.361.26612&partnerID=40&md5=7112f3c0afa08590c87e4b328dafa78d> | | reported data were insufficient to obtain a prevalence estimate | | |
|  | | Idemili-Aronu N et al., 2022 | Uptake of iron supplements and anemia during pregnancy in Nigeria | | <https://pubmed.ncbi.nlm.nih.gov/32955084/> | | did not contain specific preconceptional folic acid intake data | | |
|  | | Hawks RM et al., 2018 | Exploring preconception care: insurance status, race/ethnicity, and health in the pre-pregnancy period | |  | | did not contain specific preconceptional folic acid intake data | | |
|  | | Hardido TG et al., 2023 | Adherence to Iron-Folic Acid Among Pregnant Women Attending Antenatal Care in Southern Ethiopia, 2022 | | <https://pubmed.ncbi.nlm.nih.gov/37638333/> | | reported data were insufficient to obtain a prevalence estimate | | |
|  | | Haile MT, 2017 | Compliance to Prenatal Iron and Folic Acid Supplement and Associated Factors among Women during Pregnancy in South East Ethiopia: A Cross-Sectional Study | |  | | no full text available | | |
|  | | Grundlingh H et al., 2013 | An assessment of the implementation of the National Therapeutic Programme for pregnant women within the City of Cape Town district | | <https://pubmed.ncbi.nlm.nih.gov/23885737/> | | reported data were insufficient to obtain a prevalence estimate | | |
|  | | Govender L et al., 2021 | Assessment of the Nutritional Status of Four Selected Rural Communities in KwaZulu-Natal, South Africa | | <https://pubmed.ncbi.nlm.nih.gov/34578797/> | |  | | |
|  | | Goshu YA et al., 2018 | Women’s Awareness and Associated Factors on Preconception Folic Acid Supplementation in Adet, Northwestern Ethiopia, 2016: Implication of Reproductive Health | | <https://go.exlibris.link/nZYpRdh0> | | did not contain specific preconceptional folic acid intake data | | |
|  | | Gomes S et al., 2016 | Folate and folic acid in the periconceptional period: recommendations from official health organizations in thirty-six countries worldwide and WHO | |  | | did not contain specific preconceptional folic acid intake data | | |
|  | | Gharoro EP et al., 2000 | Pattern of drug use amongst antenatal patients in Benin City, Nigeria | | <https://www.scopus.com/inward/record.uri?eid=2-s2.0-0034017630&partnerID=40&md5=692f100a9971d098895abf0112160854> | | reported data were insufficient to obtain a prevalence estimate | | |
|  | | Gezahegn A, 2016 | Assessment knowledge and experience of preconception care among pregnant mothers attending antenatal care in West Shoa Zone Public Health Centers, 2016 | |  | | did not contain specific preconceptional folic acid intake data | | |
|  | | Gedamu S et al., 2021 | Congenital Anomalies and Associated Factors among Newborns in Bishoftu General Hospital, Oromia, Ethiopia: A Retrospective Study | | <https://pubmed.ncbi.nlm.nih.gov/338597> | | wrong population | | |
|  | | Gebreweld A et al., 2018 | Prevalence and Factors Associated with Anemia among Pregnant Women Attending Antenatal Clinic at St. Paul's Hospital Millennium Medical College, Addis Ababa, Ethiopia | | <https://pubmed.ncbi.nlm.nih.gov/30245724/> | | reported data were insufficient to obtain a prevalence estimate | | |
|  | | Gebremichael TG et al., 2020 | Adherence to iron-folic acid supplement and associated factors among antenatal care attending pregnant mothers in governmental health institutions of Adwa town, Tigray, Ethiopia: Cross-sectional study | | <https://pubmed.ncbi.nlm.nih.gov/31910215/> | | reported data were insufficient to obtain a prevalence estimate | | |
|  | | Gebremichael TG et al., 2019 | Time to start and adherence to iron-folate supplement for pregnant women in antenatal care follow up; Northern Ethiopia | | <https://pubmed.ncbi.nlm.nih.gov/31308638/> | | reported data were insufficient to obtain a prevalence estimate | | |
|  | | Gebremariam AD et al., 2017 | Adherence to iron with folic acid supplementation and its associated factors among pregnant women attending antenatal care follow up at Debre Tabor General Hospital, Ethiopia, 2017 | |  | | did not contain specific preconceptional folic acid intake data | | |
|  | | Gebregzabiherher Y et al., 2017 | The Prevalence and Risk Factors for Low Birth Weight among Term Newborns in Adwa General Hospital, Northern Ethiopia | | <https://pubmed.ncbi.nlm.nih.gov/28744313/> | | did not contain specific preconceptional folic acid intake data | | |
|  | | Gebreamlak B et al., 2017 | High Adherence to Iron/Folic Acid Supplementation during Pregnancy Time among Antenatal and Postnatal Care Attendant Mothers in Governmental Health Centers in Akaki Kality Sub City, Addis Ababa, Ethiopia: Hierarchical Negative Binomial Poisson Regression | | <https://pubmed.ncbi.nlm.nih.gov/28129344/> | | reported data were insufficient to obtain a prevalence estimate | | |
|  | | Gashaw A et al., 2021 | Risk factors associated to neural tube defects among mothers who gave birth in North Shoa Zone Hospitals, Amhara Region, Ethiopia 2020: Case control study | | <https://pubmed.ncbi.nlm.nih.gov/33901231/> | | wrong study design | | |
|  | | Fite MB et al., 2023 | Co-occurrence of iron, folate, and vitamin A deficiency among pregnant women in eastern Ethiopia: a community-based study | | <https://pubmed.ncbi.nlm.nih.gov/37353841/> | | did not contain specific preconceptional folic acid intake data | | |
|  | | Fikadu K et al., 2022 | Knowledge of pre-conception health and planned pregnancy among married women in Jinka town, southern Ethiopia and factors influencing knowledge | |  | | did not contain specific preconceptional folic acid intake data | | |
|  | | Fekadu M et al., 2022 | Peri-conception folic acid supplementation knowledge and associated factors among women visiting Maternal and Child Health clinics in Addis Ababa, Ethiopia | | <https://pubmed.ncbi.nlm.nih.gov/36303927/> | | did not contain specific preconceptional folic acid intake data | | |
|  | | Fasola O et al., 2018 | Knowledge, attitude and practice of good nutrition among women of childbearing age in Somolu Local Government, Lagos State | | <https://pubmed.ncbi.nlm.nih.gov/30079171/> | | reported data were insufficient to obtain a prevalence estimate | | |
|  | | Elmugabil A et al., 2023 | Prevalence and Associated Risk Factors for Anemia in Pregnant Women in White Nile State, Sudan: A Cross-Sectional Study | | <https://pubmed.ncbi.nlm.nih.gov/37153491/> | | did not contain specific preconceptional folic acid intake data | | |
|  | | Ekpe AC et al., 2023 | Predictors of Anaemia among Pregnant Women Booking for Antenatal Care at Federal Medical Centre, Bida, Niger State, Nigeria | | <https://pubmed.ncbi.nlm.nih.gov/37639388/> | | reported data were insufficient to obtain a prevalence estimate | | |
|  | | Ejigu T et al., 2013 | Quality of antenatal care services at public health facilities of Bahir-Dar special zone, Northwest Ethiopia | | <https://pubmed.ncbi.nlm.nih.gov/24161007/> | | reported data were insufficient to obtain a prevalence estimate | | |
|  | | Ejidokun OO, 2000 | Community attitudes to pregnancy, anaemia, iron and folate supplementation in urban and rural Lagos, south-western Nigeria | | <https://pubmed.ncbi.nlm.nih.gov/11151555/> | | reported data were insufficient to obtain a prevalence estimate | | |
|  | | EDALIA MG, 2022 | Perception and uptake of preconception care among women at reproductive health clinic: case of kenyatta national hospital nairobi city county, kenya | |  | | did not contain specific preconceptional folic acid intake data | | |
|  | | Ebuy Y et al., 2017 | Determinants of severe anemia among laboring mothers in Mekelle city public hospitals, Tigray region, Ethiopia | | <https://pubmed.ncbi.nlm.nih.gov/29099850/> | | reported data were insufficient to obtain a prevalence estimate | | |
|  | | de Weerd S et al., 2002 | Preconception counseling improves folate status of women planning pregnancy | |  | | did not contain specific preconceptional folic acid intake data | | |
|  | | Demisse TL et al., 2019 | Utilization of preconception care and associated factors among reproductive age group women in Debre Birhan town, North Shewa, Ethiopia | |  | | did not contain specific preconceptional folic acid intake data | | |
|  | | Demisse B et al., 2021 | Adherence status to iron with folic acid supplementation and associated factors among pregnant women receiving antenatal care at public health facilities in Northwest Ethiopia | | <https://pubmed.ncbi.nlm.nih.gov/34676074/> | | reported data were insufficient to obtain a prevalence estimate | | |
|  | | Demis A et al., 2019 | Iron and folic acid supplementation adherence among pregnant women attending antenatal care in North Wollo Zone northern Ethiopia: institution based cross-sectional study | |  | | did not contain specific preconceptional folic acid intake data | | |
|  | | Demeke M et al., 2022 | Knowledge and Attitude on Preconception Care and Associated Factors Among Women of Reproductive Age With Chronic Disease Who Have Follow-up at Amhara Regional State Referral Hospitals, Ethiopia, 2022; Multicenter Cross-sectional Study | |  | | participants are women with NCD | | |
|  | | Daibu U et al., 2024 | Periconceptional folic acid intake: a one year survey among mothers of patients with myelomeningocele in a regional neurosurgical centre in Northwestern Nigeria | |  | | wrong publication type | | |
|  | | Clapp MA et al., 2018 | Preconception coverage before and after the Affordable Care Act Medicaid expansions | |  | | wrong study design | | |
|  | | Chikakuda AT et al., 2018 | Compliance to Prenatal Iron and Folic Acid Supplement Use in Relation to Low Birth Weight in Lilongwe, Malawi | | <https://pubmed.ncbi.nlm.nih.gov/30201880/> | | did not contain specific preconceptional folic acid intake data | | |
|  | | Chen, J.W. et al., 2023 | Local conceptions of the role of folate in neural tube defects in Zambia | |  | | did not contain specific preconceptional folic acid intake data | | |
|  | | Chamba C et al., 2021 | Anaemia in the Hospitalized Elderly in Tanzania: Prevalence, Severity, and Micronutrient Deficiency Status | |  | | did not contain specific preconceptional folic acid intake data | | |
|  | | Cawley, S. et al., 2017 | Duration of periconceptional folic acid supplementation in women booking for antenatal care | |  | | out of SSA countries | | |
|  | | Caniglia, E.C. et al., 2022 | Iron, folic acid, and multiple micronutrient supplementation strategies during pregnancy and adverse birth outcomes in Botswana | |  | | reported data were insufficient to obtain a prevalence estimate | | |
|  | | Boti N et al., 2018 | Adherence to Iron-Folate Supplementation and Associated Factors among Pastoralist's Pregnant Women in Burji Districts, Segen Area People's Zone, Southern Ethiopia: Community-Based Cross-Sectional Study | |  | | reported data were insufficient to obtain a prevalence estimate | | |
|  | | Bodeau-Livinec F et al., 2011 | Maternal anemia in Benin: prevalence, risk factors, and association with low birth weight | |  | | wrong study design | | |
|  | | Bizuneh AD et al., 2022 | Knowledge on anaemia and benefit of iron-folic acid supplementation among pregnant mothers attending antenatal care in Woldia town, Northeastern Ethiopia: a facility-based cross-sectional study | |  | | reported data were insufficient to obtain a prevalence estimate | | |
|  | | Birhanu Z et al., 2018 | Ethiopian women's perspectives on antenatal care and iron-folic acid supplementation: Insights for translating global antenatal calcium guidelines into practice | |  | | reported data were insufficient to obtain a prevalence estimate | | |
|  | | Birhanu, T.M. et al., 2018 | Compliance to iron and folic acid supplementation in pregnancy, Northwest Ethiopia | |  | | reported data were insufficient to obtain a prevalence estimate | | |
|  | | Besho, M. et al., 2023 | Knowledge of periconceptional folic acid supplementation and associated factors among pregnant women attending antenatal care at public health facilities in Hawassa, South Ethiopia | |  | | did not contain specific preconceptional folic acid intake data | | |
|  | | Berihu BA et al., 2019 | Maternal risk factors associated with neural tube defects in Tigray regional state of Ethiopia | | https://pubmed.ncbi.nlm.nih.gov/30075882/ | | wrong study design | | |
|  | | Berhe KK et al., 2014 | Assessment of antenatal care utilization and its associated factors among 15 to 49 years of age women in Ayder Kebelle, Mekelle City 2012/2013; a cross sectional study | | (Link not provided) | | reported data were insufficient to obtain a prevalence estimate | | |
|  | | Beressa G et al., 2022 | Utilization and compliance with iron supplementation and predictors among pregnant women in Southeast Ethiopia | | https://pubmed.ncbi.nlm.nih.gov/36171347/ | | did not contain specific preconceptional folic acid intake data | | |
|  | | Benedicto W, 2020 | Determinants associated with adherence to iron-folic acid supplementation among pregnant women in Kasulu communities, north-western, Tanzania | |  | | reported data were insufficient to obtain a prevalence estimate | | |
|  | | Bello JK et al., 2022 | Preconception health service provision among women with and without substance use disorders | |  | | not done in SSA country | | |
|  | | Bekele MM et al., 2020 | Knowledge of preconception care and associated factors among healthcare providers working in public health institutions in Awi Zone, North West Ethiopia, 2019: Institutional-based cross-sectional study | |  | | wrong population | | |
|  | | Begum K et al., 2018 | Prevalence of and factors associated with antenatal care seeking and adherence to recommended iron-folic acid supplementation among pregnant women in Zinder, Niger | |  | | reported data were insufficient to obtain a prevalence estimate | | |
|  | | Begashaw B et al., 2022 | Preconception of folic acid supplementation knowledge among Ethiopian women reproductive age group in areas with high burden of neural tube defects: a community based cross-sectional study | |  | | did not contain specific preconceptional folic acid intake data | | |
|  | | Bannink F et al., 2015 | Prevention of spina bifida: Folic acid intake during pregnancy in Gulu district, northern Uganda | | https://pubmed.ncbi.nlm.nih.gov/26090048/ | | Included | | |
|  | | Balcha WF et al., 2023 | Maternal Knowledge of Anemia and Adherence to its Prevention Strategies: A Health Facility-Based Cross-Sectional Study Design | | <https://pubmed.ncbi.nlm.nih.gov/37077150/> | | reported data were insufficient to obtain a prevalence estimate | | |
|  | | Bahati F et al., 2021 | Adherence to iron and folic acid supplementation during pregnancy among postnatal mothers seeking maternal and child healthcare at Kakamega level 5 hospital in Kenya: a cross-sectional study | | <https://go.exlibris.link/qwwDnWp1> | | did not contain specific preconceptional folic acid intake data | | |
|  | | Babughirana G et al., 2020 | Maternal and newborn healthcare practices: assessment of the uptake of lifesaving services in Hoima District, Uganda | | <https://pubmed.ncbi.nlm.nih.gov/33176734/> | | reported data were insufficient to obtain a prevalence estimate | | |
|  | | Ayensu J et al., 2020 | Prevalence of anaemia and low intake of dietary nutrients in pregnant women living in rural and urban areas in the Ashanti region of Ghana | | <https://pubmed.ncbi.nlm.nih.gov/31978048/> | | reported data were insufficient to obtain a prevalence estimate | | |
|  | | Ayalew Y et al., 2017 | Women’s knowledge and associated factors in preconception care in Adet, West Gojjam, Northwest Ethiopia: a community based cross sectional study | |  | | reported data were insufficient to obtain a prevalence estimate | | |
|  | | Assefa H et al., 2019 | Magnitude and factors associated with adherence to Iron and folic acid supplementation among pregnant women in Aykel town, Northwest Ethiopia | | <https://pubmed.ncbi.nlm.nih.gov/31412795/> | | reported data were insufficient to obtain a prevalence estimate | | |
|  | | Asres AW et al., 2023 | Association between iron-folic acid supplementation and pregnancy-induced hypertension among pregnant women in public hospitals, Wolaita Sodo, Ethiopia 2021: a case-control study | | <https://go.exlibris.link/3nk7mz4D> | | wrong study design | | |
|  | | Asmamaw DB et al., 2022 | Poor adherence to iron-folic acid supplementation and associated factors among pregnant women who had at least four antenatal care in Ethiopia: a community-based cross-sectional study | | <https://pubmed.ncbi.nlm.nih.gov/36570134/> | | reported data were insufficient to obtain a prevalence estimate | | |
|  | | Argaw A et al., 2023 | Effect of prenatal micronutrient-fortified balanced energy-protein supplementation on maternal and newborn body composition: A sub-study from the MISAME-III randomized controlled efficacy trial in rural Burkina Faso | | <https://www.scopus.com/inward/record.uri?eid=2-s2.0-85166735732&doi=10.1371%2fjournal.pmed.1004242&partnerID=40&md5=d5abfa932a356cb8c1599320a850cd6f> | | wrong study design | | |
|  | | Arficho TT et al., 2023 | Level and factors associated with compliance to iron-folic acid supplementation among pregnant women in rural Soro district, Hadiya Zone, Ethiopia: cross-sectional study | | <https://pubmed.ncbi.nlm.nih.gov/37726836/> | | did not contain specific preconceptional folic acid intake data | | |
|  | | Asres AW, Hunegnaw WA, Ferede AG, Azene TW,. 2022 | Compliance level and factors associated with iron-folic acid supplementation among pregnant women in Dangila, Northern Ethiopia: A cross-sectional study. | | <https://pubmed.ncbi.nlm.nih.gov/36003078/> | | did not contain specific preconceptional folic acid intake data | | |
|  | | Arega Sadore A et al., 2015 | Compliance with Iron-Folate Supplement and Associated Factors among Antenatal Care Attendant Mothers in Misha District, South Ethiopia: Community Based Cross-Sectional Study | | <https://pubmed.ncbi.nlm.nih.gov/26839573/> | | did not contain specific preconceptional folic acid intake data | | |
|  | | Alsharief A et al., 2021 | The association between maternal micronutrients supplementation and child birthweight in sa'ad abualela hospital and suba teaching hospital, Khartoum, Sudan in 2019 | | <https://www.embase.com/search/results?subaction=viewrecord&id=L634779791&from=export> | | no full text available | | |
|  | | Alemu AA et al., 2021 | Knowledge of preconception care and its association with family planning utilization among women in Ethiopia: meta-analysis | |  | | did not contain specific preconceptional folic acid intake data | | |
|  | | Alema NM et al., 2020 | Patterns and determinants of prescribed drug use among pregnant women in Adigrat general hospital, northern Ethiopia: a cross-sectional study | |  | | reported data were insufficient to obtain a prevalence estimate | | |
|  | | Alegbeleye J et al., 2022 | Folic acid Usage and Associated factors among pregnant women attending a tertiary health facility in Nigeria | |  | | did not contain specific preconceptional folic acid intake data | | |
|  | | Al Darzi W et al., 2014 | Knowledge of periconceptional folic acid use among pregnant women at Ain Shams University Hospital, Cairo, Egypt | |  | | not done in SSA country | | |
|  | | Ahmed K et al., 2015 | Knowledge, attitude and practice of preconception care among Sudanese women in reproductive age about rheumatic heart disease | |  | | study population is only mothers with rheumatic disease | | |
|  | | Aguayo VM et al., 2005 | Acceptability of multiple micronutrient supplements by pregnant and lactating women in Mali | | <https://pubmed.ncbi.nlm.nih.gov/15705243/> | | reported data were insufficient to obtain a prevalence estimate | | |
|  | | Agegnehu G et al., 2019 | Adherence to Iron and Folic Acid Supplement and Its Associated Factors among Antenatal Care Attendant Mothers in Lay Armachiho Health Centers, Northwest, Ethiopia, 2017 | | <https://pubmed.ncbi.nlm.nih.gov/31275954/> | | reported data were insufficient to obtain a prevalence estimate | | |
|  | | Agbozo F et al., 2020 | Maternal Dietary Intakes, Red Blood Cell Indices and Risk for Anemia in the First, Second and Third Trimesters of Pregnancy and at Predelivery | | <https://pubmed.ncbi.nlm.nih.gov/32183478/> | |  | | |
|  | | Adelo ES et al., 2023 | Dietary Supplements Intake During Pregnancy Among Pregnant Women in Ethiopia | |  | | reported data were insufficient to obtain a prevalence estimate | | |
|  | | Abeje S et al., 2023 | Assessment of Iron-Folate Adherence and Associated Factors Among Pregnant Women in Public Health Facilities of Durame Town, Southern Ethiopia | | <https://pubmed.ncbi.nlm.nih.gov/37528909/> | | did not contain specific preconceptional folic acid intake data | | |
|  | | Kalipa et al 2017 | Factors Influencing Adherence to Folic Acid and Ferrous Sulphate Nutritional Intake among Pregnant Teenagers in Buffalo City Municipality, South Africa | | <https://scialert.net/fulltext/?doi=pjn.2017.531.537> | | did not contain specific preconceptional folic acid intake data | | |
|  | | Abebaw A et al., 2020 | Proportion of Immediate Postpartum Anaemia and Associated Factors among Postnatal Mothers in Northwest Ethiopia: A Cross-Sectional Study | | <https://pubmed.ncbi.nlm.nih.gov/32607255/> | | did not contain specific preconceptional folic acid intake data | | |
|  | | Abdullahi H et al., 2014 | Antenatal iron and folic acid supplementation use by pregnant women in Khartoum, Sudan | | <https://www.embase.com/search/results?subaction=viewrecord&id=L603737833&from=export> | | did not contain specific preconceptional folic acid intake data | | |
|  | | Abayneh H et al., 2022 | Knowledge, attitude, and practice of preconception care and associated factors among obstetric care providers working in public health facilities of West Shoa Zone, Ethiopia: A cross-sectional study | |  | | study participants are health professionals | | |
|  | | Teshome F et al., 2021 | Practice of Preconception Care and Associated Factors among Pregnant Women in Manna District, Southwest Ethiopia: A Community-Based Cross-Sectional Study | | <https://www.longdom.org/open-access/practice-of-preconception-care-and-associated-factors-among-pregnant-women-in-manna-district-southwest-ethiopia-a-communitybased-c-64576.html> | | included | | |
|  | | Setegn Alie et al., 2022 | Preconception care utilization and associated factors among reproductive age women in Mizan-Aman town, Bench Sheko zone, Southwest Ethiopia, 2020. A content analysis | | <https://pubmed.ncbi.nlm.nih.gov/35984828/> | | included | | |
|  | | Olowokere AE et al., 2015 | Awareness, knowledge and uptake of preconception care among women in Ife Central Local Government Area of Osun State, Nigeria | | <https://www.ajol.info/index.php/jcmphc/article/view/139391> | | included | | |
|  | | Okon UA et al., 2020 | Awareness and use of folic acid among women of childbearing age in Benue State, Nigeria | | <https://pubmed.ncbi.nlm.nih.gov/33209187/> | | included | | |
|  | | Nwaolisa HI et al., 2021 | Perception of preconception care among women attending antenatal care clinic in a tertiary health care hospital in south east, Nigeria | | <https://magnascientiapub.com/journals/msarr/sites/default/files/MSARR-2021-0036.pdf> | | included | | |
|  | | Mukhalisi A et al., 2022 | UTILIZATION OF PRECONCEPTION CARE SERVICES AMONG COUPLES IN BUNGOMA COUNTY, KENYA | | <https://www.iprjb.org/journals/index.php/JHMN/article/view/1478?srsltid=AfmBOor2XY0jpN8HIuwQp2YUvjzxMR5LFJVgd_9HyF8gkbrc7rtwPM0c> | | included | | |
|  | | Mohammed BS et al., 2020 | Prevalence and determinants of uptake of folic acid in peri‐conceptional period in a rural lower‐middle‐income country, Ghana | | <https://pubmed.ncbi.nlm.nih.gov/31599082/> | | included | | |
|  | | Lawal TA et al., 2014 | Determinants of folic acid intake during preconception and in early pregnancy by mothers in Ibadan, Nigeria | | <https://pubmed.ncbi.nlm.nih.gov/25722786/> | | included | | |
|  | | Joyce C, 2018 | Utilization of Preconception Care Services among Women of Reproductive Age in Kiambu County, Kenya | | <https://ir-library.ku.ac.ke/server/api/core/bitstreams/3fda3cf1-bd41-4114-bcd4-24097fc67888/content> | | included | | |
|  | | Hassan AA et al., 2024 | Periconceptional folic acid usage and its associated factors in eastern Sudan: A cross-sectional study | | <https://pubmed.ncbi.nlm.nih.gov/38279794/> | | included | | |
|  | | Habte A et al., 2021 | Determinants of practice of preconception care among women of reproductive age group in southern Ethiopia, 2020: content analysis | | <https://pubmed.ncbi.nlm.nih.gov/34020669/> | | included | | |
|  | | Goshu YA et al., 2018 | Preconception care utilization and its associated factors among pregnant women in Adet, North-Western Ethiopia (Implication of Reproductive Health) | | <https://www.longdom.org/open-access/preconception-care-utilization-and-its-associated-factors-among-pregnant-women-in-adet-northwestern-ethiopia-implication-of-reprod-2167-0420-1000445.pdf> | | included | | |
|  | | Girma A et al., 2023 | Utilization of Preconception Care and Its Determinants Among Pregnant Women Attending ANC in Private MCH Hospitals in Addis Ababa, Ethiopia: A Cross Sectional Descriptive Study | | <https://pubmed.ncbi.nlm.nih.gov/37684575/> | | included | | |
|  | | Gelgalu T, 2021 | Preconception Care Utilization and Associated Factors Among Pregnant Women Attending Antenatal Care in Public Health Facilities of Shashemene south eastern Ethiopia: Institutional-Based CrossSectional Study | | <https://scholar.google.com/scholar?hl=en&as_sdt=0%2C5&q=+Gelgalu+T.+Preconception+Care+Utilization+and+Associated+Factors+Among+Pregnant+Women+Attending+Antenatal+Care+in+Public+Health+Facilities+of+Shashemene+south+eastern+Ethiopia%3A+Institutional-Based+CrossSectional+Study+2021.+&btnG=> | | included | | |
|  | | Gedefaw A et al., 2018 | Magnitude of Neural Tube Defects and Associated Risk Factors at Three Teaching Hospitals in Addis Ababa, Ethiopia | | <https://pubmed.ncbi.nlm.nih.gov/29713643/> | | included | | |
|  | | Gbemileke A et al., 2023 | Assessing women's knowledge on benefits of iron and folic acid and its consumption during pregnancy in northern Nigeria | | <https://go.exlibris.link/jbTykZ31> | | included | | |
| 1. 19 | | Gamshe EN, Demissie DB, 2022 | Perinatal Factors Affecting Knowledge and Utilization of Preconception Care among Pregnant Women at Selected Hospitals in Addis Ababa: A Cross-Sectional Study | | <https://mjh.sphmmc.edu.et/MJH-2021-0001/MJH-2021-0001.pdf> | | included | | |
|  | | Fetena N et al., 2023 | Utilization of preconception care and associated factors among pregnant mothers in Fiche Town, Central Ethiopia: a community-based cross-sectional study 2021 | | <https://pubmed.ncbi.nlm.nih.gov/37795507/> | | included | | |
|  | | Fekene DB et al., 2020 | Knowledge, uptake of preconception care and associated factors among reproductive age group women in West Shewa zone, Ethiopia, 2018 | | <https://pubmed.ncbi.nlm.nih.gov/32075638/> | | included | | |
|  | | Ekem NN et al., 2018 | Utilisation of preconception care services and determinants of poor uptake among a cohort of women in Abakaliki Southeast Nigeria | | <https://pubmed.ncbi.nlm.nih.gov/29526148/> | | included | | |
|  | | Dessie MA et al., 2017 | Folic acid usage and associated factors in the prevention of neural tube defects among pregnant women in Ethiopia: cross-sectional study | | <https://pubmed.ncbi.nlm.nih.gov/28934941/> | | included | | |
|  | | Degu Ayele et al., 2022 | Preconception Care Utilization and Its Associated Factors Among Women in Debre Tabor Town Northwest Ethiopia: Community Based Cross-Sectional Study | | <https://journals.sagepub.com/doi/full/10.1177/21582440221097392> | | included | | |
|  | | Boakye-Yiadom AK et al., 2020 | Preconception care: awareness, knowledge, attitude And practice of pregnant women, tamale west hospital | | <https://ajpojournals.org/journals/index.php/AJHMN/article/view/516/0?srsltid=AfmBOopZyu0N4JvDinWrDN6orw_byAUV4czGJVXvyBZ-_HwLQaiZtcBC> | | included | | |
|  | | Beyuo T et al., 2021 | Knowledge and Utilization of Preconception Care Services Among Pregnant Women Attending Antenatal Care at The Korle Bu Teaching Hospital | | <https://journal.gcps.edu.gh/index.php/pmjg/article/view/263> | | included | | |
|  | | Asumadu OKD et al., 2020 | PRECONCEPTION KNOWLEDGE AND PRACTICES AMONG WOMEN IN FERTILITY AGE IN THE TAMALE TEACHING HOSPITAL OF GHANA | | <https://ajpojournals.org/journals/index.php/EJHS/article/view/568?srsltid=AfmBOorg4zpUUtIQwlhKvvTTNAoSUYLmIhmqAaq7hInAKyhKO6w3R0n4> | | included | | |
|  | | Asresu TT et al., 2019 | Mothers’ utilization and associated factors in preconception care in northern Ethiopia: a community based cross sectional study | | <https://pubmed.ncbi.nlm.nih.gov/31601190/> | | included | | |
|  | | Anzaku AS, 2013 | Assessing folic acid awareness and its usage for the prevention of neural tube defects among pregnant women in Jos, Nigeria | | <https://www.ajol.info/index.php/jbcrs/article/view/115382> | | included | | |
|  | | Amaje E et al., 2022 | Utilization of Preconception Care and Its Associated Factors among Pregnant Women of West Guji Zone, Oromia, Ethiopia, 2021: A Community-Based Cross-Sectional Study | | <https://pubmed.ncbi.nlm.nih.gov/35342774/> | | included | | |
|  | | Alsammani MA et al., 2017 | Factors associated with folic acid knowledge and intake among pregnant women in Sudan | | <https://pubmed.ncbi.nlm.nih.gov/29270966/> | | included | | |
|  | | Alemajo CA et al., 2022 | Peri-conceptional folic acid supplementation: A Cross-Sectional study to Assess the Awareness, Knowledge, Use and Associated Factors Among Pregnant Women Attending Antenatal Care in a Secondary Health Care Facility, Southwest Region, Cameroon | | <https://www.fortunejournals.com/articles/periconceptional-folic-acid-supplementation-a-crosssectional-study-to-assess-the-awareness-knowledge-use-and-associated-factors-am.pdf> | | included | | |
|  | | Akinajo OR et al., 2019 | Preconception care: Assessing the level of awareness, knowledge and practice amongst pregnant women in a tertiary facility | | <https://journals.lww.com/jocs/fulltext/2019/16030/preconception_care__assessing_the_level_of.3.aspx> | | included | | |
