# Supplementary material for: Folic acid supplementation during preconception period in sub-Saharan African countries: A systematic review and meta-analysis
Source: PLoS One. 2025 Jan 31;20(1):e0318422. doi: 10.1371/journal.pone.0318422 (PMC11785287; doi:10.1371/journal.pone.0318422)
Supplement: Attachment — Submitted filename: Response to Reviewers.docx [file pone.0318422.s006.docx]

**Date: July 19, 2024**

To: Plos One, Editorial Office

Subject: **Submitting a revised version of manuscript and point by point response**

Manuscript ID: PONE-D-24-13717

Title: Folic acid supplementation during preconception period in sub-Saharan African countries: A systematic review and meta-analysis

Dear Editorial Office;

We are pleased to submit the revised version of our manuscript titled "Folic acid supplementation during preconception period in sub-Saharan African countries: A systematic review and meta-analysis" (Manuscript ID: PONE-D-24-13717). In response to the academic editor and reviewers' comments and suggestions, we have made substantial revisions to improve the clarity and quality of our manuscript. We believe these changes have strengthened our study, and we are grateful for the constructive feedback provided.

Enclosed with this letter, please find our detailed point-by-point response to the reviewers' comments. Each comment has been addressed meticulously, and the corresponding revisions are highlighted in the manuscript for your convenience.

We appreciate the opportunity to revise and resubmit our work to PLOS ONE. We look forward to your positive response.

Regards,

Authors

**Point by point response to the Academic editor and reviewer’s comments**

| 1. **Comments from the academic editor and responses** | |
| --- | --- |
| **Academic editor’s comments** | **Authors’ responses** |
| please ensure that your manuscript meets PLOS ONE's style requirements | **Dear editor,**  **Thank you for the opportunity to submit our manuscript.**  We have meticulously ensured that our manuscript meets all PLOS ONE style requirements. |
| We noticed you have some minor occurrence of overlapping text with the following previous publication(s), which needs to be addressed | **Dear Editor,**  We appreciate your attention to this matter and will promptly address and resolve the minor occurrences of overlapping text with the previous publication |
| Please complete your Competing Interests on the online submission form to state any Competing Interests | **Dear Editor,**  Thank you for the reminder. We have completed the Competing Interests section on the online submission form and we included it with cover letter. |
| We note that you have referenced (unpublished) on page 6. Please remove this from your References and amend this to state in the body of your manuscript: (ie “Bewick et al. [Unpublished]”) as detailed online in our guide for authors | **Dear Editor,**  We apologize for the confusion caused. Upon review, we can confirm that there is no unpublished citation on page six, and there is no reference named Bewick et al. in the manuscript.  Sorry for any confusion this may have caused. |
| Please include captions for your Supporting Information files at the end of your manuscript, | **Dear Editor,**  Thank you for your recommendation. We have included captions for all Supporting Information files at the end of our manuscript as requested**.** |
| **2. Comments from reviewer #1 and responses** | |
| **Reviewer’s comments** | **Authors’ responses** |
| **Abstract:** Add the number of studies and participants included in the review in the results section of the abstract. | **Dear reviewer,**  Thank you for your suggestion. We have revised the abstract to include the number of studies and participants included in the review as requested. |
| **Introduction:**  NTDs has been spelled out more than once. Spell out once when used for the first time and then use the acronym for the rest of the manuscript. | **Dear reviewer,**  Thank you for your feedback. We have revised this in the manuscript and we Spelled out "NTDs" in the introduction once and use acronyms thereafter throughout the manuscript unless the term comes at the beginning of the stetment. |
| Some of the references in the introduction are very old. I suggest replacing these with the more recent references. | **Dear reviewer,**  Thank you for your suggestion. We replaced the older references in the introduction with more recent ones to ensure the currency of the information.  Thank you once again for your valuable suggestion |
| I found the introduction very weak. There are quite a few recent relevant references assessing the existing evidence, guidelines, and policies around folic acid supplementation. I suggest that the authors look at these papers and add some of the relevant references in their introduction to make a stronger case for the review | **Dear reviewer,**  Thank you for your suggestion. We appreciate the feedback and we have reviewed recent relevant literature to strengthen the introduction by incorporating recent relevant references related to folic acid supplementation. The revised version of the manuscript incorporated the suggested updated articles and sources. This will enhance the robustness of our review. |
| . Add some references (if available) on country specific prevalance rates of NTDs in Africa. | **Dear reviewer,**  Thank you for the suggestion. We have incorporated recent relevant references on country-specific prevalence rates of neural tube defects in Africa to provide a more comprehensive introduction, aligning with existing evidence and guidelines on folic acid supplementation. |
| The acronym for sub-sahraan Africa (SSA), folic acid (FA) and low- middle- income countries (LMICs) have been used inconsistently throughout the manuscripot. I would suggest that the authors spell it out when used for first time in the darft and then consistently use the acronym throughout | **Dear reviewer,**  Thank you for your feedback.  We have corrected it and ensured consistency in the use of acronyms such as SSA (Sub-Saharan Africa), FA (folic acid), and LMICs (low- and middle-income countries) throughout the manuscript. |
| Not sure what this sentence under 'study selection' means: "articles examining the prevalence and determinants of folic acid supplementation during preconception period was gathered from various". From the objectives and results, it seems that only prevalance was assessed. I am not sure whether the authors ahve also looked at the determinants? and if they have, this has not be reported anywhere in the draft. | **Dear reviewer,**  We apologize for any confusion caused by the initial statement. The manuscript has been updated to accurately reflect our findings. The revised statement now reads:  *'A comprehensive collection of articles assessing the prevalence of folic acid FA supplementation during the preconception period was gathered from various sources’*  *Thank you again.* |
| Not sure what this sentence means: "A quantitative cross-sectional study design was employed to enhance clarity and significance." Do you mean that the eligibility for study selection was cross-sectional study design? If yes, then state this clearly. | **Dear reviewer,**  Thank you for your query regarding the statement 'A quantitative cross-sectional study design was employed to enhance clarity and significance.'  We apologize for any confusion caused by this sentence. The intention of this statement was to clarify that our review specifically included studies employing quantitative methodologies, while excluding those using qualitative approaches.  We revised the statement in the manuscript to explicitly state: “*Quantitative studies were included to enhance clarity and significance.”* |
| Suggest authors add a table or a figure depicting the regions and the respective countries included in sub-saharan Africa. It would add clarity for the readers as teh regions and respective countries have been disucssed and analysed in the resultss and discussion sections | **Dear reviewer,**  Thank you for your valuable suggestion to add a table or figure depicting the regions and respective countries included in Sub-Saharan Africa.  We have now included a table in the main manuscript under the characteristics of the studies section. This table outlines the regions of Sub-Saharan Africa and the respective countries within each region. |
| Study design is stated twice under data extraction | **Dear reviewer,**  Thank you for your observation. We have removed the duplicate mention of "study design" under the data extraction section*.* |
| . **Result**  I cant see the full characterictis of the inlcuded studies table (probably because it was in a landscape mode) so I am not sure if my comments are relevant. But I would suggest that the authors delete the columns on study period, center, sampling method, and response rate. I would also suggest that the authors add the columns on sample size and study results (reported prevalance) in the study characteristics table | **Dear Reviewer**,  Thank you for your feedback regarding the table of included studies. We apologize if the landscape format made it difficult to view the entire table.  Based on your suggestions, we made the following adjustments:  We have deleted the columns on study period, center, sampling method, and response rate.  We have Added columns for sample size and study results (reported prevalence). |
| I would suggest that the authors remove the study quality column from the study characteristics table and add a separate section on study quality. | **Dear Reviewer**,  Thank you for your feedback regarding the study characteristics table. We agree that separating the study quality information into its own section would enhance clarity. As such, we removed the study quality column from the study characteristics table and we have prepared a separate section dedicated to study quality assessment.  Thank You once again for your recommendation. |
| I would suggest that the authors re-organise the discussion section starting with summarising the reveiw findings, quality of the included studies, comparing the review findings with other studies/reviews, strengths and limitations of the review and research and practice implications. | **Dear Reviewer,**  Thank you for your valuable suggestion regarding the re-organization of the discussion section. We appreciate your feedback and agree that restructuring the section will enhance the clarity and coherence of our manuscript. In the discussion section, we summarized key findings, evaluated study quality, compared results with existing literature, and assessed methodological strengths and limitations. We concluded by discussing implications for future research and clinical practice. |
| I would suggest that the authors compare the study findings with studies conducted in similar context. For eaxmple, reference 59 is a study from Italy and hence widely differs in context. However, authors can broadly discuss the differences in the folic acid supplementation prevalance between high income and low- middle- income countries. | **Dear Reviewer,**  Thank you for your valuable feedback. We agree that Reference 59, a study from Italy, differs significantly in context compared to our study setting. To address this, we have broadened our discussion to compare folic acid supplementation prevalence between high-income and low- to middle-income countries. Although studies in this area are limited, we have made efforts to address this gap through a comprehensive search of existing literature.  Thank you once again for your insightful suggestion. |
| **3. Comments of reviewer #2 and responses** | |
| **Abstract:** **Introduction:** Seems very long for an abstract. Just a suggestion: instead of reporting global estimates of NTDs I would suggest writing the estimates from Sub Saharan Africa. I dont think daily dosage is required here. | **Dear Reviewer,**  Thank you for your feedback. We have focused on Sub-Saharan Africa estimates of NTDs and omitted daily dosage details to make the abstract more concise.  Thank you for your suggestions. |
| Methods:  Please add on what type of studies did you include? Last date of search? if GRADE was conducted and how was quality of included studies conducted (if you did any of these) | **Dear Reviewer,**  Thank you for your feedback. We have revised the Methods section to include more details about the types of studies included, the last date of the search, and the quality assessment process using newcastele-otwa scale  Thank you for your suggestions. |
| Results:  Line 44: not sure what do you mean by 'individual studies' here.  Line 46: subgroup analysis based on?  Line 49: remove double brackets from the end  Quality of included studies in missing | **Dear Reviewer,**  Thank you for your feedback.  Line 44: We apologize for any confusion caused. We have now clarified our statement.  Line 46: We conducted subgroup analysis based on corresponding countries.  Line 49: Thank you for your observation. We have removed the unnecessary information.  We employed the Newcastle-Ottawa Scale for quality assessment, as highlighted in the abstract and we have included it the main document.  Thank you once again for your careful review and for improving the abstract section. |
| Introduction  If you have introduced the abbreviation once, then you dont have to introduce it again and again. e.g., NTDs. | **Dear Reviewer,**  Thank you for your feedback.  Introduction: We have corrected it and ensured consistency in the use of abbreviations, such as NTDs, once introduced.  Thank you for your attention to detail and your suggestions for improving clarity in our manuscript. |
| Methods:  I would suggest to rearrange some headings. After registration, you should talk about the eligibility criteria and then search strategy and study selection and so on | **Dear Reviewer,**  Thank you for your suggestion.  Methods: We have rearranged the headings to discuss eligibility criteria immediately following registration, followed by the search strategy, study selection, and subsequent sections.  Thank you for guiding us in improving the organization of our methods section. |
| Line 117: you have stated that you search institutional repositories. Please state which were those? | **Dear Reviewer,**  **Thank you for your comment.**  We attempted to search the institutional repositories of several universities, namely Addis Ababa University, Jimma University, Hawassa University, and Bahir Dar University**.**  **Thank you for your attention to this detail and your valuable feedback.** |
| Line 120: Please check punctuation: "supplementation, and folic acid administration. folic acid adherence, folinic acid, and vitamin B9." | **Dear Reviewer, Thank you for your comment.**  Line 120: We have corrected the punctuation as follows: *“preconception care, preconception, pre-conception, periconception, peri-conception, before conception, folic acid, folate, vitamin, pteroylglutamic acid, folic acid supplementation, folic acid administration, folic acid adherence, folinic acid and vitamin B9”.*  **Thank you for bringing this to our attention and for your careful review.** |
| Line 127-129: This sentence is very vague "A quantitative cross-sectional study design was employed to enhance clarity and significance' | **Dear Reviewer,**  **Thank you for your feedback.**  Line 127-129: We acknowledge the vagueness of the sentence "A quantitative cross-sectional study design was employed to enhance clarity and significance." We revised this statement to provide more specific details about our study design.  **Thank you for your thoughtful review and suggestions for improving clarity in our manuscript.** |
| Line 127: 'omitted' is not an appropraite word to use here | **Dear Reviewer,**  Thank you for your feedback.  Line 127: We acknowledge that "omitted" is not appropriate in this context. We have revised the wording accordingly to accurately describe our methodology.  Thank you for bringing this to our attention and for your careful review. |
| I think you need to revisit this paragraph. It requires language editing  Linme 127-129: " A quantitative cross-sectional study design was employed to enhance clarity and significance. In contrast, studies utilizing qualitative methodologies were omitted due to the specific nature of the review and the analytical approach selected for this review" I think this should be discussed under section 2.4. | **Dear Reviewer,**  **Thank you for your comments.**  We have revised the paragraph under subsection” **Inclusion criteria and exclusion criteria** ” as follows:  *“Quantitative studies were included to enhance clarity and significance. In contrast, studies utilizing qualitative methodologies were excluded due to the specific nature of the review and the chosen analytical approach."*  Thank you for your feedback and for guiding us in improving the clarity of our manuscript. |
| Line 131: I understand you used RAYYAN then why did you exported data on Endnote? | **Dear Reviewer,**  Thank you for your question.  Line 131: We used RAYYAN for the initial screening because of its user-friendly interface and collaboration features. After that, we exported the data to Endnote for managing references and ensuring consistent citation formatting.  Thank you for helping us clarify this in our manuscript |
| Section 2.3 says study selection but it doesnt talks clearly about it. You have not written about title/abstract and full text screening. How conflicts were managed?  Lacks lot of important information | **Dear Reviewer,**  Thank you for your feedback.  Section 2.3: We have revised the section to clearly describe the study selection process. Here is the updated text:  "*All duplicate studies were removed, and full-text studies were downloaded. Titles and abstracts were initially scrutinized based on specific criteria to exclude irrelevant studies. Those that passed this stage underwent full-text screening, where their design, methodology, sample size, and key findings were assessed for relevance and suitability. The eligibility of each study was independently evaluated by two reviewers (BM & EA). Any significant differences in their assessments were resolved through discussion and consultation with other reviewers (MN & AK)."*  Thank you for pointing out these gaps and helping us improve the clarity and completeness of our manuscript. |
| Section 2.4: A lot of important information is missing. I am confused. Did you include cross sectional studies and grey literature or only cross sectional studies. | **Dear Reviewer,**  Thank you for your feedback.  Section 2.4: We acknowledge the need for clarification and additional detail in this section.  We included cross-sectional studies and also considered grey literature in our review.  We have revised this section to provide a clearer explanation of our inclusion criteria and search methods.  Thank you for guiding us in improving the clarity and completeness of our manuscript**.** |
| Line 136: better to use the word 'included' rather than incorporated. | **Dear Reviewer,**  **Thank you for your suggestions.**  We have revised the wording to use "included" instead of "incorporated" for clarity and readability. |
| Suggest adding a Table on PECO criteria | **Dear Reviewer,**  **Thank you for your suggestions.**  We appreciate the suggestion to include a Table outlining our PECO criteria. We have now incorporated a Table in the manuscript that concisely outlines our Population, Exposure, Comparator, and Outcome (PECO) criteria.  Thank you for suggesting us for better improvement of the document. |
| Line 146: suggest using the word 'resolved' instead of 'fixed' | **Dear Reviewer,**  Thank you for your suggestion. We have revised the manuscript to use the word 'resolved' instead of 'fixed'. |
| Line 153 and 155: please avoild using such vague words/phrases: 'the required information' 'important parameters' | **Dear Reviewer,** Thank you for your feedback.  We have revised the paragraph to enhance clarity in the paragraph as per your suggestion.  Thank you for your valuable input. |
| Line 157: study setting in repeated twice | **Dear Reviewer,**  Thank you for your feedback.  We have revised the stetment to address the repetition of 'study setting' |
| Line 156: If you are only including cross-sectional studies then why are you collecting data on study designs. | **Dear Reviewer,** Thank you for your request.  Initially, we reviewed all observational studies, excluding only experimental, case report, or case series studies. Eventually, we found that all identified studies meeting our inclusion criteria were cross-sectional in nature. |
| Line 162: Add reference of Stata | **Dear Reviewer,**  **Thank you for your suggestion.**  We have incorporated the reference to Stata software as the following  *“The extracted data were transferred to STATA/MP version 17.0 software(StataCorp LLC, College Station, TX, USA) for analysis.”* |
| Line 166-168: The sentences are repetitive. | **Dear Reviewer,**  Thank you for pointing out the repetition in lines 166-168. we have revised the sentences to ensure clarity and eliminate redundancy. Here is the improved version:  ***“****Statistical assessment of significant heterogeneity was conducted using the Cochrane Q-test and I^2^ statistics.****”*** |
| A lot of missing information in methods sections: did you conduct cross-referencing. did you felt need of contacting authors for missing data. You stated that you searched institutional website there is no mention of inclusion of reports in the eligibility criteria. | **Dear Reviewer,**  Thank you for your detailed feedback. We have revised the Methods section to include the missing information you highlighted.  .When necessary, we contacted study authors to obtain missing data to ensure comprehensive analysis. and we conducted cross referencing.  We conducted a quality assessment of the included studies using the Newcastle-Ottawa Scale (NOS) and we revised the manuscript according to your suggestions.  In addition to peer-reviewed articles, we included relevant reports from institutional websites in our eligibility criteria and we mention the institutions in the main document. |
| Why did you not assess the quality of evidence using GRADE? | **Dear Reviewer,**  Thank you for your feedback. In our study, we used the Newcastle-Ottawa Scale (NOS) to assess the quality of included cross-sectional studies. This scale was chosen for its suitability in evaluating study quality based on criteria specific to cross-sectional designs.  We acknowledge the importance of the GRADE approach in systematic reviews, however, for our study, which primarily focused on assessing the quality of cross-sectional studies, we chose to use the Newcastle-Ottawa Scale (NOS).  Thank you once again for your valuable feedback. |
| On what basis did you conduct the sensitivity analysis? high heterogeneity or low quality of studies? How did you "assess the influence of individual studies on pooled estimates"? | **Dear Reviewer,**  Thank you for your inquiry regarding the basis and methodology of our sensitivity analysis.  We conducted sensitivity analyses to assess the impact of individual studies on the overall prevalence estimate in our study. Despite a thorough evaluation, excluding any single study did not result in a statistically significant alteration in the pooled prevalence estimate.  Furthermore, we performed additional sensitivity analyses by excluding studies identified as having poor quality. Even after excluding these lower-quality studies, the estimated prevalence remained stable without significant changes.  In addition to address heterogeneity, we used regression by factors and identified that sample size had a statistically significant relation to the presence of heterogeneity.  Thank you for your valuable feedback and attention to these methodological aspects. |
| Results:  Please Format your headings: "3.1 selection and Identification of studies" | **Dear reviewer**  Thank you for your comment.  We revised based on your comment and it is now formatted ***"3.1 selection and Identification of studies"*** |
| Why did you exclude study based on poor quality? You could have done a sensitivity analysis for it | **Dear reviewer**  Thank you for your suggestions for better improvement of the review.  We included both high quality and low quality studies and sensetivity analysis was done based on the quality of the studies to evaluate how the quality of studies influenced our findings. These details are outlined in the revised "Sensitivity Analysis" subsection.  However, during our rigorous evaluation process, one study was excluded from our final meta-analysis due to its exceptionally poor quality which may introduce biases that could affect the reliability of our review's findings. This decision was pivotal in safeguarding the reliability and validity of our results.  Thank you once again for your valuable suggestions |
| Line 198: spell out 2. Suggest spelling out numbers <10 | **Dear reviewer,**  Thank you for your carful review and valuable suggesions  we have revised the text accordingly, ensuring that all numbers less than ten are spelled out to enhance clarity and readability.  We appreciate your attention to detail and your contribution to improving the quality of our manuscript. |
| Please dont use the word 'individual studies'.. you can say 'included studies' | **Dear reviewer,**  Thank you for your suggestion. We have revised the paragraphs to spell out all phrases of “individual study” to “included studies” for clarity and readability.  We appreciate your attention to detail. |
| Table 1 doesn't fit the page and is not clear. what does NS mean? what is R. rate? response rate? | **Dear reviewer,**  Thank you for your feedback. We apologize for the formatting issue with Table 1 and any confusion it may have caused. We have adjusted the table format to ensure it fits within the page and is more legible. Regarding the abbreviations we have corrected it to full words and we revised all the table heading by including only relevant data based on the suggesions.  Thank you again for your careful review. |
| Not sure why did you do subgroup analysis based on study period? What would you get from it? | **Dear reviewer,**  Thank you for your question.  We conducted a subgroup analysis based on the study period to evaluate whether there have been any changes in the prevalence of folic acid supplementation over time in Sub-Saharan African countries.  However, our results showed that there was no significant difference in the prevalence of folic acid supplementation across the different time periods analyzed.  We appreciate your attention to this aspect of our methodology and are happy to provide further clarification if needed. |
| Quality of included studies MISSING! in text. | **Dear reviewer,**  Thank you for your suggestion  We have revised and we included the quality of the included studies in the result section.  Thank you for your concern. |
| Discussion:  I would suggest that you should summarise your study findings in the first para and then discuss about other studies. | **Dear reviewer,**  Thank you for your suggestion. We have revised our discussion section accordingly. In the first paragraph, we have summarized the main findings of the review. |
| Lines 270-274: Can be moved later in the discussion under strengths. | **Dear reviewer,**  Thank you for the reccomandation. According to the reccomendation we revised it and we moved the stetments to the strength section for better clarity and consistency of the review.  Thank you again for your comments. |
| Lines 289-291: not sure why are you comparing prevalence of Italy with your results? There is no link. These are two different regions/countries. You can compare with LMICs buy why with Italy? | **Dear reviewer,**  Thank you for your valuable feedback.  We have carefully revised the manuscript as per your suggestion to remove the comparison with Italy, as it wasn't directly relevant to our study's focus. Instead, we have emphasized comparisons with other low- and middle-income countries (LMICs), which better align with the socio-economic and healthcare contexts pertinent to our study area.  We appreciate you bringing this to our attention. |
| You have not discussed on how quality of studies can affect the findings of your study? | **Dear reviewer,**  Thank you for your recommendation. Based on your suggestion, we have revised the discussion section to include the quality of the studies and the interpretation of the results accordingly.  Thank you for your valuable comment. |
| Conclusion would come after limitations and strengths | **Dear reviewer,**  **Thank you for your valuable suggestion.**  We have reorder it according to the suggestion and the conclusion section comes after limitation and strength of the study.  Thank you again for this sugession. |
| Figure 1: Why were studies excluded based on title and abstract twice? | **Dear reviewer,**  We apologize for the confusion caused by the typographical error in Figure 1 regarding the exclusion criteria based on title and abstract. The studies were indeed excluded based on title and abstract only once. We have corrected this error in the revised figure.  Thank you for your careful review and valuable comments**.** |
| General Comments:  Did you find studies on peri-conception? if yes, then I would suggest doing a subgroup analysis based on preconception and periconception supplementation. | **Dear Reviewer,**  Thank you for your inquiry.  Our systematic review focused exclusively on preconceptional folic acid supplementation, and therefore, all included studies focused on FA supplementation during preconceptional period.  **We appreciate your feedback and guidance throughout this process.** |
| Why did you included studies on pregnant women when you intention was preconception? Did they collect reterospective data? | **Dear Reviewer,**  Thank you for your inquiry.  While our primary focus was on preconceptional folic acid supplementation, some studies included pregnant women due to their retrospective data collection methods. These studies gathered information on folic acid intake both before conception and during pregnancy, allowing us to capture a broader perspective on supplementation practices in the population.  **Thank you for your insightful question and valuable feedback.** |
| What were the ages of the included participants? suggest doing a subgroup analysis bases of age and see the prevalence in young people | **Dear Reviewer,**  Thank you for your query regarding the ages of included participants in our systematic review.  The included studies encompassed most studies included age groups some studies include participants aged greater than 15,but specific prevalence data for younger age groups were not consistently reported across all studies. As a result, subgroup analysis based on age, particularly focusing on young adults, was not feasible.  Thank you for your valuable suggestion |
| Did you do sensitivity analysis based on low quality of studies? if not then suggest doing that too | **Dear Reviewer,**  Thank you for your question.  We did conduct a sensitivity analysis to assess the impact of study quality on our findings.  Based on your suggestion, we have included a detailed description of the sensitivity analysis in the revised manuscript. |
| **We appreciate the reviewer's and editor’s thoughtful comments and suggestions, which have significantly contributed to enhancing the clarity and robustness of our manuscript.** | |
