# Supplementary material for: Folic acid supplementation during preconception period in sub-Saharan African countries: A systematic review and meta-analysis
Source: PLoS One. 2025 Jan 31;20(1):e0318422. doi: 10.1371/journal.pone.0318422 (PMC11785287; doi:10.1371/journal.pone.0318422)
Supplement: Attachment — Submitted filename: Response to Reviewers.docx [file pone.0318422.s007.docx]

**Date: September 20, 2024**

To: Plos One, Editorial Office

Subject: **Submitting a revised version of manuscript and point by point response**

Manuscript ID: PONE-D-24-13717R1

I am pleased to resubmit our revised manuscript titled, "Folic acid supplementation during preconception period in sub-Saharan African countries: A systematic review and meta-analysis" (ID: PONE-D-24-13717R1). We have carefully considered and addressed the reviewers' valuable comments, making revisions that we believe have strengthened the clarity and quality of the manuscript.

This work highlights an important public health issue, and we are grateful for the opportunity to improve it based on the feedback provided. We hope it now meets the high standards of PLOS ONE and look forward to your assessment. Thank you for your time and consideration.

Regards,

Authors

**Point by point response to the Academic editor and reviewer’s comments**

| 1. **Comments from the academic editor and responses** | |
| --- | --- |
| **Academic editor’s comments** | **Authors’ responses** |
| Submit a revised version of the manuscript that addresses the points raised during the review process. | **Dear editor,**  **Thank you for the opportunity to submit our manuscript.**  we have thoroughly addressed and incorporated all of the reviewers' comments and suggestions into the revised version. We greatly appreciate the time and effort the editorial team have invested in reviewing our work, and we believe that these revisions have strengthened the manuscript. |
| **2. Comments from reviewer #1 and responses** | |
| **Reviewer’s comments** | **Authors’ responses** |
| The review title and objectives suggest that the authors aimed to review the prevalance of folic acid supplementation during preconception period which is defined as the period of time before pregnancy occurs. However, the authors have used terms for 'periconception' as well in their search strategy. Moreover, the included studies had a variety of target population "eight studies (28.57%) were women of childbearing age, 16 studies 257 (57.1%) centered on pregnant mothers, and four studies (14.3%) included postnatal mothers." Majority of these studies (16 studies) included pregnant women and does not follow the eligibiltiy crirteria for inclusion which is preconception supplementation. The only studies eligible for inlcusion as per the authors eligibility criteria are studies that targeted either women of reproductive age or postnatal women. I would suggets that the authors clarify the definition of "preconception" used for this review to reflect the correct eligibility and inclusion. | **Dear reviewer,**  Thank you for your valuable feedback We understand your concern regarding the search terms we used and the target populations of the included studies.  We used the term "periconception" in our search strategy because some studies report folic acid supplementation during the preconception period separately, even though the studies were conducted during the periconception phase. By including "periconception," we aimed to ensure that we captured all relevant studies, particularly those that might have reported preconceptional folic acid intake under the broader term "periconception.” So, we specifically selected only those that reported on folic acid supplementation during the preconceptional(before pregenency) period.  Regarding the inclusion of studies involving pregnant, we included studies with pregnant participants because they assessed folic acid (FA) intake during the preconception period by asking women about their supplementation practices before the pregnancy based on recall. Even though these studies involve pregnant women at the time of the survey, they provided valuable information about preconceptional FA intake. However, we did not include studies that reported FA supplementation during pregnancy. Our aim was to focus specifically on preconceptional FA supplementation to ensure the relevance of the data.  Thank you for your detailed review and for highlighting these points. We appreciate your insights and hope this clarifies our approach. |
| **3. Comments of reviewer #2 and responses** | |
| Manuscript require thorugh read anf formatting edits. There are places were there is no space between text, references, and brackets e.g., Line 108: individuals(7).The ; Line 82: In sub-Saharan Africa(SSA). Punctuation is not used appropriately e.g. like 127: .(28). - Remove extra full stops where evere required.  Please read your manuscript thoroughly and make the required edits where necessary. | **Dear reviewer,**  Thank you for your feedback. We have reviewed and corrected the formatting issues, including spacing between text, references, and brackets, and adjust punctuation errors such as extra full stops.  We appreciate your assistance and will make the necessary edits. |
| Abstract:  Introduction is still very long. Please reduce some text  - Line 31: Remove extra brackets and full stop  - Line 40: Add space between of and FA | **Dear reviewer,**  Thank you for your carful review and your relevant comments. According to the suggestions we reduced the texts from the introduction of abstract section and we have corrected the errors of and we removed in appropriate punctuations. Thanks again for your suggestions. |
| Line 152: This line is very confusing where you say that you are including ALL journal articles. However you are excluding qualitative studies. I get what you are trying to say here but its still very confusing. "We included all types of studies published as journal articles, theses, and dissertations without imposing restrictions based on publication date or age criteria." | Dear reviewer,  Thank you for requesting the clarity of the confusing stetment. We have rewrite the statement as the following and we hope it is clear.  “We included journal articles, theses, and dissertations without imposing restrictions based on publication date or age criteria.” |
| Section 2.2 (Line 150): First write what was included and then write what was excluded. This brings more clarity.  line 149: Which quantitative studies did you include? please state. List all the study designs which you intended to included and in results you can state that you only found cross-sectional studies. | **Dear reviewer.**  Thank you for your valuable comments and suggestions. We have corrected the order of the statements, first specifying the included studies and then the excluded ones. We included cross-sectional, cohort, and case-control studies, but ultimately found only cross-sectional studies in our review.  We mentioned the included study designs in the main document.  Thank you again for your efforts to improve the manuscript. |
| Section 2.3:  - Line 164: "were taken" is not an appropriate work to use here  - In this section first talk about making the search strategy by using Mesh terms and key words. Then talk about databases searched. | **Dear reviewer,**  Thank you for your comment. We have wrote the stetment according to the comment as the following.  “Studies published in the English language up to January 2024 were retrieved from EMBASE, MEDLINE, Scopus, CINAHL, and manually on Google and Google Scholar.”  In addition in this section first we mentioned about mesh terms and key words then we explained about data base search.  Thank you for again for your important comments. |
| Section 2.4:  Line 176: Unclear | **Dear Reviewer,**  Thank you for your comment.  We apologize for the confusion caused by the unclear statement. We have corrected it in the main document as follows:  “We systematically reviewed and evaluated studies from various sources to ensure a comprehensive analysis.” |
| Line 177: "The search was not constrained by the publication year; hence, articles published until January 2024, were considered for review eligibility." You have already mentioned the last publication date in section 2.3. The remaining details on restrictions should be moved to section 2.3. | **Dear reviewer,**  Thank you for your comment. We agree that we have already explained this under section 2.3 and we have removed the statement from Line 177, as the details about publication year constraints are already explained in Section 2.3. |
| Line 180: The information is not making any sense to me. As 'systematic review" everything should be done systematically. Even contacting authors comes after data extraction. Not before screening. Please read some good published systematic reviews and suggest rewritting your methodology. | **Dear reviewer,**  Thank you for your feedback. We acknowledge that contacting authors should occur after data extraction in a systematic review. We have corrected the arrangement of stetments and we mentioned contacting Authors for missed data were under the data extraction section. |
| Line 186: This should come before title abstract screening. | Dear reviewer,  Thank you for your valuable suggestion. To maintain the sequence of the review process, we moved the statement from Line 86 to precede the abstract and title screening stetments. |
| Section 2.6 will come before 2.5 | **Dear reviewer,**  Thank you for your feedback. We have revised the manuscript to present the section 2.6 before section 2.5.  Thank you again for your relevant suggestions. |
| Section 2.6:  Did you summarise data in Excel or you conducted data extraction in excel? Please clarify | **Dear reviewer,**  Thank you for your question. We apologize for the confusion that cuase in the writing this process. We conducted the data extraction using Microsoft Office Excel and we clarify it in the main document this process. |
| Did you cross-reference studies for missing studies? | **Dear reviewer.**  Thank you for the feedback. We have cross-referenced the included studies to ensure no relevant studies were missed. |
| Through NewCastle you assess the quality of included studies. Through GRADE you assess quality of evidence. both are two different things. However, I would suggest to use GRADE. Let us know if you dont have the capacity to do GRADE. | **Dear reviewer,**  Thank you for your valuable feedback. We have carefully assessed the certainty of evidence using the GRADE approach, as requested. The evaluation was conducted independently by two authors (E.A. and M.C.), with any disagreements resolved by consensus. This process has been clearly documented in the revised manuscript. We appreciate your suggestion, which has helped improve the clarity and transparency of our study. |
| Results:  Line 231: "We reviewed the full texts of the remaining 195 articles for eligibility, 163 studies were not eligible, and 32 studies were identified." what do you mean by 32 were identified? | **Dear Reviewer,**  Thank You for your request regarding to the clarity of the stetment. We corrected the stetment in the main document as the following;  “*We reviewed the full texts of the remaining 195 articles for eligibility. Of these, 163 studies were not eligible, and 32 studies were eligible for inclusion in our review”* |
| Rephrase line 238 | **Dear reviewer,**  Thank You for your suggestion. We rephrased the statement as the following;  “*We excluded three studies from the analysis: two conducted in Ethiopia(36, 37) and one in Uganda(38), because they reported a prevalence of folic acid supplementation during the preconception period as zero percent (0%).”* |
| - Line 259: I think it should be table 2?  Please place all the tables appropriately.  Table 2: Suggest showing this in a graph and move table 2 to supplementary file. | **Dear Reviewer,**  Thank you for your suggestion. We have moved Table 2, which covers the quality of the study, to the supplementary file and placed the other tables in their appropriate locations as needed. We appreciate your guidance. |
| Table 3: You said that you included pregnant women because they reterospectively collected data then how are those cross-secrional studies. wont those be reterospective cohort studies? Or these were cross-sectional who collected data based on recall. I just want to have an understanding on what type of studies were these. | **Dear reviewer,**  Thank you for your clarification request. The studies were cross-sectional whose target population were pregnant women. However, this study collected data by asking pregnant women to recall and report their folic acid (FA) supplementation history from before they became pregnant. |
| You are talking about sensitivity analysis in Section 3.5 and then the actual analysis comes in Section 3.7. I would suggest moving Section 3.7 to Section 3.5 | **Dear reviewer,**  Thank you for pointing that out. To improve the flow, we have moved Section 3.7 to Section 3.5, aligning the sensitivity analysis with its results for improved coherence**.** |
| - Line 357-360: There is no connection of discussing quality of studies of your review after discussing the intervention from existing evidence. Suggest moving it afetr line 341 and try to be more cohesive. | **Dear reviewer,**  Thank you for the suggestion. We moved the discussion on study quality to follow after line 341 to enhance coherence and ensure a better connection with the preceding content on the intervention. |
| - Line 361: I think you discussion need more working. First, Talk about the evidence coming from you study, including the subgroup, sentivity and quality of studies. then compare with other studies conducted in Africa followed by studies conducted in LMIC and HIC countries. Then talk about strengths and limitations. | **Dear reviewers,**  Thank you for your valuable suggestions. We have thoroughly revised the discussion section. We have incorporated evidence from our study, including subgroup analysis, sensitivity, and study quality, followed by comparisons with African studies and research from LMICs and HICs. We have also addressed the strengths and limitations of our work. We appreciate your helpful comments and feedback. |
| **We appreciate the reviewer's and editor’s thoughtful comments and suggestions, which have significantly contributed to enhancing the clarity and robustness of our manuscript.** | |
