# Supplementary material for: Folic acid supplementation during preconception period in sub-Saharan African countries: A systematic review and meta-analysis
Source: PLoS One. 2025 Jan 31;20(1):e0318422. doi: 10.1371/journal.pone.0318422 (PMC11785287; doi:10.1371/journal.pone.0318422)
Supplement: Attachment — Submitted filename: Response to Reviewers.docx [file pone.0318422.s008.docx]

**Date: Nov 13, 2024**

To: Plos One, Editorial Office

Subject: **Submitting a revised version of manuscript and point by point response**

Manuscript ID: PONE-D-24-13717R1

I am pleased to resubmit our revised manuscript titled, "Folic acid supplementation during preconception period in sub-Saharan African countries: A systematic review and meta-analysis" (ID: PONE-D-24-13717R1). We have carefully considered and addressed the reviewers' valuable comments, making revisions that we believe have strengthened the clarity and quality of the manuscript.

This work highlights an important public health issue, and we are grateful for the opportunity to improve it based on the feedback provided. We hope it now meets the high standards of PLOS ONE and look forward to your assessment. Thank you for your time and consideration.

Regards,

Authors

**Point by point response to the Academic editor and reviewer’s comments**

| 1. **Comments from the academic editor and responses** | |
| --- | --- |
| **Academic editor’s comments** | **Authors’ responses** |
| Please review your reference list to ensure that it is complete and correct. If you have cited papers that have been retracted, please include the rationale for doing so in the manuscript text, or remove these references and replace them with relevant current references. Any changes to the reference list should be mentioned in the rebuttal letter that accompanies your revised manuscript. If you need to cite a retracted article, indicate the article’s retracted status in the References list and also include a citation and full reference for the retraction notice.. | **Dear editor,**  Thank you for your helpful feedback. Upon carefully reviewing the reference list, we found that two issues needed to be address the following citation was mistakenly cited in the manuscript:  Gbemileke A, Atta AO, Abolade T, Adegoke F, Arogundade E, Kung'u J, Beckworth C. Assessing women's knowledge on benefits of iron and folic acid and its consumption during pregnancy in northern Nigeria. Annals of Nutrition and Metabolism. 2023;79:636.  we have correct reference that should have been included is:  *‘Adebo OO, Dairo DM, Ndikom CM, Adejumo PO. Knowledge and uptake of folic acid among pregnant women attending a secondary health facility in Nigeria. British Journal of Midwifery. 2017 Jun 2;25(6):358-64.’*  This reference is accurate and has now been included in the updated reference list. Thank you for your understanding and continued guidance. |
| Please specify that in the method results studies done one pregenent women were only included if they had taken folate during preconception and clarify that the studies only included these women or were there a mix | **Dear Editor,**  Thank you for your clarification question and recommendation to clarify this clearly in the manuscript. We have only included studies with pregenent women participants when they were take folate before pregnency/during preconception period/. To make clearer we have mentioned it in the updated manuscript as the following. “*Studies were included if the women had taken folate before pregnancy.”* |
| I would suggest not to use ‘prevalence’ and rather use ‘proportion’ as there were studies included which were conducted in facilities and did not have a sampling frame to assess prevalence and even few community studies would have done so. The authors need to define weather the studies covered few geographic locations or were national level. | **Dear editor,**  Thank you for your suggestion to use proportion rather than prevalence due to the nature of the included studies. we have accepted and using proportion is more convenient than proportion. So we have corrected it accordingly. Thank you again for your valuable suggestion**.** |
| In the result and prisma diagram specify how many studies were included from grey litrature | **Dear Editor,**  Thank you for your request to specify the number of grey literatures in PRISMA flow diagram.  We have specified the number of grey literatures included to this SRMA study in the PRISMA flow diagram.  Thank you again for your valuable comments.  . |
| The introduction is lengthy and to be shortened | **Dear Editor,**  Thank you for your recommendation to shorten the introduction section. We have made it shorten with removing unnecessary words and phrases to make stronger and more informative introduction. Thank you again for your suggestion |
| Why two studies zero prevalence were excluded from the analysis as they have reported the proportion and this can add to the meta-analysis. if there is specific reason the authors should state that. | **Dear Editor**  Thank you for your concern and request regarding to exclusion zero prevalence studies.  We excluded studies reporting zero prevalence from the analysis because, after closer reviewing them, we found that folic acid (FA) intake wasn’t the main focus of these studies. While they reported zero prevalence for FA intake, this wasn’t a central finding for their research, which made us concerned about how relevant and reliable that data would be for our meta-analysis. Additionally, the sample size and the methods used in these studies could have led to inaccurate reporting of zero prevalence. Including them could have introduced bias and affected the overall results, so we decided to exclude them to ensure the accuracy and reliability of our findings. |
| In the table of included studies – please also specify the scale of the included studies – whether from one village, district, province or national and if from facilities – then how many facilities were covered | **Dear Editor,**  Thank you for your insightful suggestion to specify the scale/level of the included studies. We have now clarified the scale/level for each study, and for those conducted in health facilities, we have included the number of facilities in the table.  Once again, we appreciate your valuable feedback. |
| Can the authors add a sub-group by urban/rural | **Dear Editor,**  Thank you for your suggestion to conduct a sub-group analysis by urban/rural. However, since most of the studies were conducted in health facilities with participants from both urban and rural areas, we were unable to perform the sub-group analysis. |
| Please thoroughly check the manuscript for language and grammar | **Dear Editor,**  Thank you for your suggestion to review the manuscript for language and grammar errors. We have carefully reviewed and corrected the manuscript and remain open to any additional suggestions or comments regarding any missed errors. |
| **2. Comments from reviewer #1 and responses** | |
| **Reviewer’s comments** | **Authors’ responses** |
| Reviewer #1: The authors have responded to the concerns raised over the last two rounds of review. I am happy with the editors recommendations if authors can make the edits to the eligibility criteria specifying that only data from studies where outcomes were specific to preconception supplementation were included. As the readers might get a bit confused looking at the 'participants' column in the characteristics studies. | Dear Reviewer,  Thank you for your crucial and helpful comments throughout the review process. Regarding the study participants, we have clarified that all the included studies focused on women were included only if women took folate before pregnancy. Thank you again for your valuable feedback. |
| **2. Comments from reviewer #2 and responses** | |
| Table 1: add brackets around C after Comparison  Line 188: remove extra full stops.  Line 187: 'data' should be in lowercase  Check spacing: sampling method(random/non-random) | Dear Reviewer,  Thank you for your comments and suggestions throughout the review process. Based on your feedback, we have corrected the typographical errors by removing extra full stops, adding brackets, changing the "D" in "data" to lowercase, and removing double spacing. We appreciate your valuable comments and suggestions. |
| Table 5: I did inquire initially about using Gradepro for GRADE. I would suggest giving low/moderate/serious etc ranking for each domain and providing the explanation of each ranking as footnotes. | **Dear reviewer,**  Thank you for your inquiry. Based on your recommendation and the GRADE assessment criteria, we have revised the table and added footnotes with explanation of each ranking. |
| **We appreciate the reviewer's and editor’s thoughtful comments and suggestions, which have significantly contributed to enhancing the clarity and robustness of our manuscript.** | |
