# Supplementary material for: Folic acid supplementation during preconception period in sub-Saharan African countries: A systematic review and meta-analysis
Source: PLoS One. 2025 Jan 31;20(1):e0318422. doi: 10.1371/journal.pone.0318422 (PMC11785287; doi:10.1371/journal.pone.0318422)
Supplement: Attachment — Submitted filename: Response to Reviewers.docx [file pone.0318422.s009.docx]

**Date: December 24, 2024**

**To: PLOS ONE Editorial Office**

**Subject: Resubmission of Revised Manuscript (ID: PONE-D-24-13717R1)**

**Dear Editors,**

We are pleased to resubmit our revised manuscript, "Folic Acid Supplementation During the Preconception Period in Sub-Saharan African Countries: A Systematic Review and Meta-Analysis" (ID: PONE-D-24-13717R1). We have carefully addressed the editor's comments and made revisions to enhance the manuscript's clarity and quality. Thank you for the opportunity to improve our work, and we look forward to your assessment.

Sincerely,

The Authors

**Point by point response to the Academic editor and reviewer’s comments**

| 1. **Comments from the academic editor and responses** | |
| --- | --- |
| **Academic editor’s comments** | **Authors’ responses** |
| Please review your reference list to ensure that it is complete and correct. If you have cited papers that have been retracted, please include the rationale for doing so in the manuscript text, or remove these references and replace them with relevant current references. Any changes to the reference list should be mentioned in the rebuttal letter that accompanies your revised manuscript. If you need to cite a retracted article, indicate the article’s retracted status in the References list and also include a citation and full reference for the retraction notice. | Thank you for your feedback. We have reviewed the references and replaced the incomplete citation, *"Organization WH. Global health estimates (GHE)–Cause-specific mortality. 2015. 2015*," with the complete and relevant reference:  *Flores A, Vellozzi C, Valencia D, Sniezek J. Global burden of neural tube defects, risk factors, and prevention. Indian Journal of Community Health. 2014;26(Suppl 1):3.*  We also replaced  *World Health Organization. Prevention of neural tube defects. Department of Making Pregnancy Safer Switzerland: World Health Organization. 2006*  with the more relevant and complete citation:  *Gomes S, Lopes C, Pinto E. Folate and folic acid in the periconceptional period: recommendations from official health organizations in thirty-six countries worldwide and WHO. Public Health Nutrition. 2016 Jan;19(1):176-89.*  We have replaced the incomplete reference:  The GRADE handbook H. Schünemann, J. Brożek, G. Guyatt and A. Oxman Cochrane Collaboration London, UK 2013  with the complete and updated citation:  *Guyatt GH, Thorlund K, Oxman AD, Walter SD, Patrick D, Furukawa TA, Johnston BC, Karanicolas P, Akl EA, Vist G, Kunz R. GRADE guidelines: 13. Preparing summary of findings tables and evidence profiles—continuous outcomes. Journal of Clinical Epidemiology. 2013 Feb 1;66(2):173-83.*  While we have ensured accuracy, we will accept if any incomplete references remain that may make tracking difficult. |
| i would like to thank the authors for the revisions. I have one remaining comment about the three studies reporting zero prevalence being excluded from the analysis.  The authors should either exclude these studies altogether from the review or give the reason in the manuscript for not including them in the meta-analysis and if folic acid (FA) intake wasn’t the main focus of these studies, then why were they included. Also I am not clear how the authors assessed if the sample size and the methods used in these studies were not accurate (as the Newcastle-Ottawa Scale ratings do not suggest so) and how was this ascertained for all the studies that were included. i did have a look at it and did not see any compelling issues as preconception care was assessed in these studies and IFA was one component assessed. Please elaborate on this. | Dear Editor,  Thank you for your insightful feedback and for pointing out this issue. We excluded the three studies with zero prevalence. These studies reporting zero prevalence were excluded from the review because their primary focus was not on folic acid (FA) intake during the preconception period. While these studies report zero prevalnce during preconception, FA supplementation was only a minor component, which could significantly influence the results and potentially compromise the reliability of the review findings.  Excluding these studies from review strengthens the specificity and reliability of our systematic review and meta-analysis in estimating FA supplementation during the preconception period. We have updated the manuscript accordingly and appreciate your guidance in helping us improve the quality of our work.  Thank you once again for your support to enhance the manuscript. |
