# Supplementary material for: Folic acid supplementation during preconception period in sub-Saharan African countries: A systematic review and meta-analysis
Source: PLoS One. 2025 Jan 31;20(1):e0318422. doi: 10.1371/journal.pone.0318422 (PMC11785287; doi:10.1371/journal.pone.0318422)
Supplement: Attachment — Submitted filename: Response to Reviewers.docx [file pone.0318422.s010.docx]

**Date: Jan 1, 2025**

**To: PLOS ONE Editorial Office**

**Subject: Resubmission of Revised Manuscript (ID: PONE-D-24-13717R1)**

**Dear Editors,**

We are pleased to resubmit our revised manuscript, "Folic Acid Supplementation During the Preconception Period in Sub-Saharan African Countries: A Systematic Review and Meta-Analysis" (ID: PONE-D-24-13717R1). We have carefully addressed the editor's comments and made revisions to enhance the manuscript's clarity and quality. Thank you for the opportunity to improve our work, and we look forward to your assessment.

Sincerely,

The Authors

**Point by point response to the Academic editor and reviewer’s comments**

| 1. **Comments from the academic editor and responses** | |
| --- | --- |
| **Academic editor’s comments** | **Authors’ responses** |
| I would like to thank the authors for all the hard work and making the revisions and addressing most comments but I taking am still not clear on why the three studies with zero-prevalence are being excluded as the reasons that 'primary focus was not on folic acid (FA)' and a 'minor component' are not strong enough reasons as previously they were included in the review though not in meta-analysis. Also the exclusion criteria of your review does not specify exclusion on these reasons. The quality assessment done by the authors for the three studies is also not very different from the other studies and especially the domain of 'Assessment of outcome'. So I would suggest to include these three or provide a strong reason (within the pre-set exclusion criteria) and how it varies from all the other included studies. | Dear Editors We appreciate your thoughtful comments and suggestion. The decision to exclude these three studies was not only 'primary focus was not on folic acid (FA)' and a 'minor component but also based on the fact that their results were extremely low, reporting zero values, which are considered outliers in this context.  Including these studies in the meta-analysis would not allow for a standard pooled estimate, as zero values lead to a negative standard deviation, which is not acceptable. To resolve this, a continuity correction formula would need to be applied, but doing so would still introduce bias by modifying the numbers inappropriately. Therefore, to maintain the robustness and integrity of the study, we decided to exclude these studies. We have added the exclusion criteria to the Methods section for greater clarity and transparency. However, we remain open to your suggestions and are happy to reconsider the approach if necessary. Thank you again for your insightful comments and suggestions. |
| **Journal Requirements:** | **Authors’ responses** |
| Please review your reference list to ensure that it is complete and correct. If you have cited papers that have been retracted, please include the rationale for doing so in the manuscript text, or remove these references and replace them with relevant current references. Any changes to the reference list should be mentioned in the rebuttal letter that accompanies your revised manuscript. If you need to cite a retracted article, indicate the article’s retracted status in the References list and also include a citation and full reference for the retraction notice. | . We have reviewed the reference list, and none of the cited papers have been retracted. All references are complete and correct |
